# Exploring Multi‐Subsite Binding Pockets in Proteins: DEEP‐STD NMR Fingerprinting and Molecular Dynamics Unveil a Cryptic Subsite at the GM1 Binding Pocket of Cholera Toxin B†

**DOI:** 10.1002/chem.202001723

**Published:** 2020-07-20

**Authors:** Serena Monaco, Samuel Walpole, Hassan Doukani, Ridvan Nepravishta, Macarena Martínez‐Bailén, Ana T. Carmona, Javier Ramos‐Soriano, Maria Bergström, Inmaculada Robina, Jesus Angulo

**Affiliations:** ^1^ School of Pharmacy University of East Anglia Norwich Research Park NR4 7TJ Norwich UK; ^2^ Department of Biochemistry & Molecular Biology Sealy Center for Structural Biology & Molecular Biophysics University of Texas Medical Branch 301 University Blvd Galveston TX 77555-1068 USA; ^3^ Department of Organic Chemistry Faculty of Chemistry University of Seville 41012 Seville Spain; ^4^ Department of Chemistry and Biomedical Sciences Linnaeus University 391 82 Kalmar Sweden; ^5^ Instituto de Investigaciones Químicas (CSIC-US) Avda. Américo Vespucio, 49 41092 Sevilla Spain

**Keywords:** cholera toxin inhibitors, DEEP-STD NMR, ligand-based NMR spectroscopy, multi-subsite binding pockets, protein–ligand interactions

## Abstract

Ligand‐based NMR techniques to study protein–ligand interactions are potent tools in drug design. Saturation transfer difference (STD) NMR spectroscopy stands out as one of the most versatile techniques, allowing screening of fragments libraries and providing structural information on binding modes. Recently, it has been shown that a multi‐frequency STD NMR approach, differential epitope mapping (DEEP)‐STD NMR, can provide additional information on the orientation of small ligands within the binding pocket. Here, the approach is extended to a so‐called DEEP‐STD NMR fingerprinting technique to explore the binding subsites of cholera toxin subunit B (CTB). To that aim, the synthesis of a set of new ligands is presented, which have been subject to a thorough study of their interactions with CTB by weak affinity chromatography (WAC) and NMR spectroscopy. Remarkably, the combination of DEEP‐STD NMR fingerprinting and Hamiltonian replica exchange molecular dynamics has proved to be an excellent approach to explore the geometry, flexibility, and ligand occupancy of multi‐subsite binding pockets. In the particular case of CTB, it allowed the existence of a hitherto unknown binding subsite adjacent to the GM1 binding pocket to be revealed, paving the way to the design of novel leads for inhibition of this relevant toxin.

## Graphical abstract

**Fingerprinting**: A powerful protocol by using NMR spectroscopy and molecular dynamics to study multi‐subsite binding pockets has been developed. For cholera toxin subunit B (CTB), the combination of differential epitope mapping saturation transfer difference (DEEP‐STD) NMR, STD competition experiments, transfer‐NOESY, and Hamiltonian replica exchange molecular dynamics (HREMD) unveiled the presence of a novel subsite adjacent to the known subsites of GM1 on CTB.

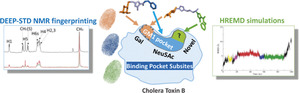

## Introduction

In the context of drug discovery, fragment‐based drug discovery (FBDD) has gained momentum over the last decade as a method for lead generation.^1^ The technique relies on screening small compounds against a pharmacologically relevant target to find fragments with low affinity towards some of the adjacent binding *subsites* of which the target's binding pocket can be formally thought as composed of. The binding fragments are then combined together to obtain potent leads. In the FBDD approach, detailed information on the target structural features is in fact necessary.^2^

On these premises, it is not surprising that NMR spectroscopy is one of the elected methods to rationally design novel drugs by using FBDD, particularly for low affinity binders, which tend to generate protein–ligand complexes that are difficult to crystallize. Back in 1997, the first NMR methodology for efficient ligand screening and lead design was proposed: “SAR by NMR” (structure–activity relationship by NMR).^3^ In SAR by NMR, isotopically labeled proteins are studied with each fragment of a particular library, and chemical shift perturbations allow, upon protein assignment, identification of different subsite binders. Analysis of further NOESY experiments gives rational directions to connect fragments, to obtain very high affinity leads.^3^ A complementary approach, based on observation of NMR signals of the binder, is the use of ligand‐based NMR techniques.^4^ Within this approach, one decade later, Pellecchia and co‐workers proposed a method based on transfer‐NOESY experiments: “SAR by Inter‐Ligand NOE” (ILOE). Without the need of labeling and spectroscopic assignment of the targets, it is possible to study a small pool of fragments, accommodated in different adjacent binding subsites within a binding pocket, and provide points of contact between them to obtain inhibitors of nanomolar potency.^5^

Among ligand‐based NMR approaches, Saturation Transfer Difference NMR (STD NMR)^6^ spectroscopy has proved itself to be an efficient and reliable tool in FBDD, allowing us to discriminate between specific and unspecific binders, even when they are analyzed as a pool.^7^ We have recently proposed an expansion of the methodology termed Differential Epitope Mapping STD NMR (DEEP‐STD NMR) based on the new concept of multi‐frequency STD NMR experiments.^8^ In DEEP‐STD NMR spectroscopy, two binding epitope mappings are determined, following the use of two different frequencies for protein saturation, in contrast to a single one as in standard STD NMR. By changing the on‐resonance frequency, different types of side chains in the binding pocket will reach slightly different levels of saturation and those differences can be picked up by building a differential epitope mapping, where the differences between the two mappings are revealed. Those differences indicate proximity to the type of protein side chain directly irradiated in each experiment, providing information on the type of amino acid side chain surrounding a proton of the ligand, as well as hinting about the orientation of the ligand relative to the side chains lining the binding pocket. This way, further to identifying selective binders, the technique allows us to determine the orientation of the fragments in the binding subsites. This is extremely valuable information when it comes to drug design, and previous results from our laboratory have shown DEEP‐STD NMR to be a very informative tool in the discovery of WWP2 ubiquitin ligase inhibitors.^9^

Here, we have applied DEEP‐STD NMR spectroscopy, combined with advanced molecular dynamics simulations to explore the plasticity and dynamics of the multi‐subsite binding pocket of cholera toxin B (CTB). The interaction between CTB and its natural substrate, the glycolipid ganglioside GM1, is one of the strongest and best studied protein–carbohydrate interactions known, and is biologically highly relevant, as it mediates the onset of the cholera infection.^10^ Currently, still, cholera treatment mainly resorts to antibiotics, but the appearance of antibiotic resistant *V. cholera* strains^11^ have triggered the development of alternative cures for this endemic disease.^12^ Large efforts have been reported towards the design of inhibitors of the GM1/CTB interaction. Structural and thermodynamic studies on this complex showed that high specificity is given by the presence of two adjacent binding subsites, accommodating the two “arms” of the GM1 ligand, that is, the galactose and sialic acid non‐reducing ends.^13^, ^14^, ^15^ Grounded in this knowledge, many carbohydrate‐based scaffolds have been designed that aim to target both binding subsites.^16^, ^17^, ^18^, ^19^, ^20^

**Figure 1:**
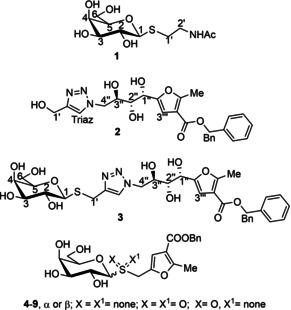
Structure of ligands **1**, **2**, and **3** 21 and the novel set of thio‐galactoside CTB ligands **4**–**9** described in this work.

Despite these glycomimetics showing very good affinity levels, the challenge has remained to overcome the problem of hydrolyzability. In our labs, we have reported that CTB ligands based on thio‐galactosides bearing a polyhydroxyalkylfuroate moiety (PHF), showed promise as non‐hydrolyzable inhibitors.^21^ The PHF moiety was demonstrated to contribute effectively to the affinity for CTB, and STD NMR studies on three representatives of this family of CTB ligands (Figure 1, compounds **1**–**3**) supported a bidentate binding mode, so that both the galactose ring and the PHF moiety are the main contacts with the protein in the bound state of ligand **3**.

In this work, we present the synthesis of a new set of CTB ligands to explore the GM1 binding pocket of CTB and propose the DEEP‐STD NMR fingerprinting protocol, to gain orientational information on the ligands in the subsites by comparison to DEEP‐STD NMR data of a binder with a known 3D structure of its complex to CTB. The combination of STD NMR competition experiments,^22^ ILOEs,^23^ docking,^24^, ^25^, ^26^ advanced molecular dynamics,^27^ and STD NMR intensity prediction from 3D molecular models of the complexes using CORCEMA‐ST,^28^ has allowed us to answer the question of how large and flexible ligands like **3** can be accommodated in the GM1 binding pocket of CTB, demonstrating the existence of a hitherto unknown binding subsite adjacent to the GM1 binding pocket, paving the way to the design of novel non‐carbohydrate leads for CTB inhibition.

## Results and Discussion

A novel set of promising small‐molecule CTB inhibitors are developed to explore the binding subsites of the GM1 binding pocket of CTB by DEEP‐STD NMR fingerprinting. The STD NMR results previously obtained for CTB ligand **3** 21 indicated that both (galactose and benzyl) ends of the binder established the closest contacts with CTB in the bound state, whereas the polyhydroxyalkyl spacer made less contact with the protein. As no 3D structure of the **3**/CTB complex is available, this result could be interpreted as a simultaneous binding of both ends to the galactose and sialic acid subsites within the GM1 binding pocket of CTB (bidentate bound mode). Based on these results, we first wanted to experimentally probe the role in CTB binding of the polyhydroxyalkyl spacer linking both ends in **3**, to inform the size optimization of novel CTB ligands. To that aim, we designed a novel set of shorter thio‐galactoside ligands (compounds **4**–**9**, Figure 1) lacking the triazole‐polyhydroxyalkyl linker present in **2** and **3** and that instead have the thio‐galactose residue directly connected to the benzyl furoate moiety. The variability across the compound set is given by the anomeric configuration (α or β) and the oxidation state of the glycosidic sulfur (present as a thioether, sulfone, or sulfoxide functional group), for a total of six new compounds (Figure 1).

**Scheme 1:**
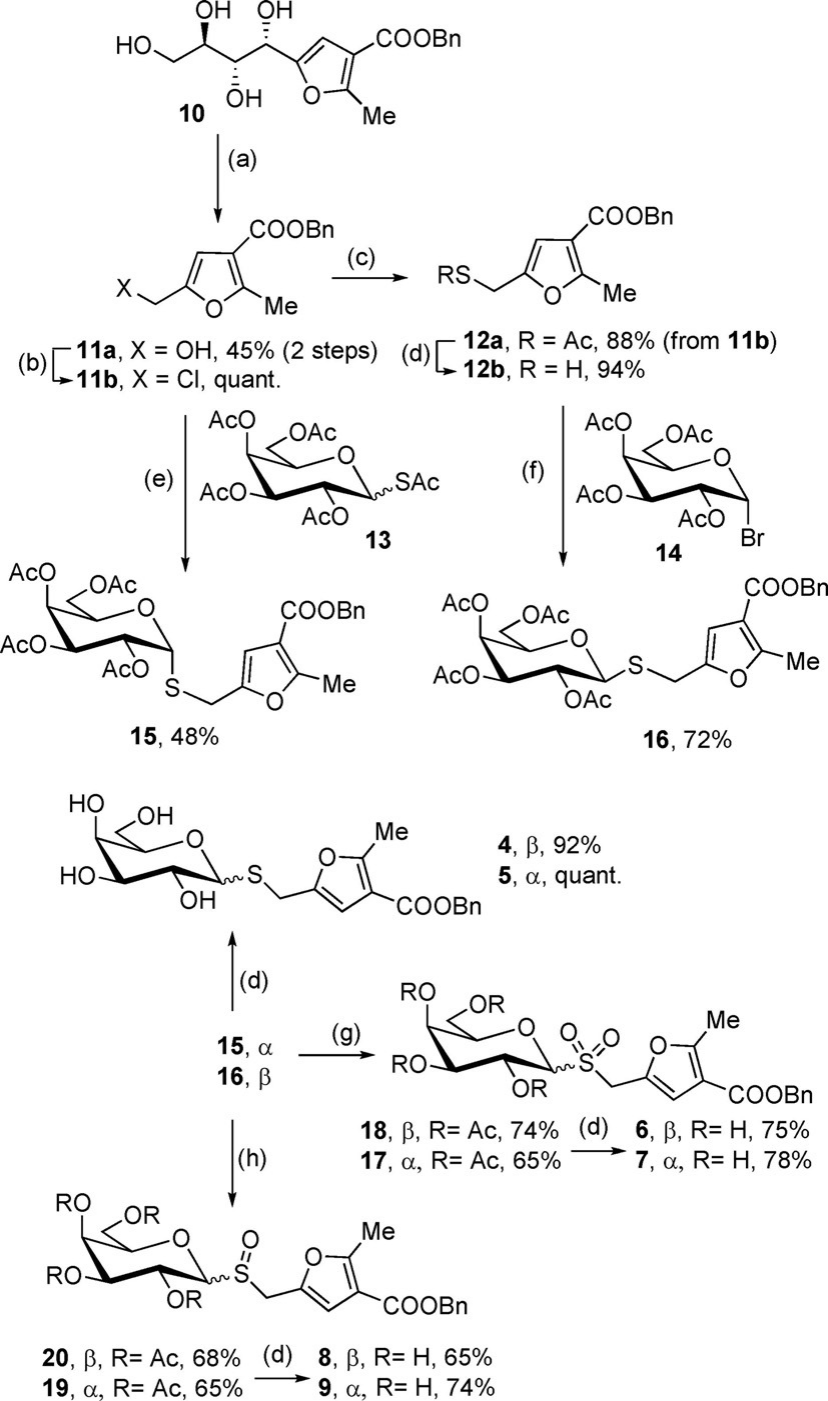
Reagents and conditions: a) 1) NaIO_4_, H_2_O/MeOH, 0 °C→ RT; 2) NaBH_4_, MeOH, RT; b) NCS, Me_2_S, CH_2_Cl_2_, −20→ 0 °C; c) KSAc, DMF, 50 °C; d) NaOMe, MeOH, 0 °C; e) EtNH_2_, DMF, RT; f) aq. Na_2_CO_3_, EtOAc, TBAHS, RT; g) MCPBA, CH_2_Cl_2_, RT; h) MCPBA, CH_2_Cl_2_, −78→ −30 °C.

The synthesis of α‐galactosides **5**, **7**, and **9** (Scheme 1) started from peracetylated thioglycosides **13**.^29^ Selective S‐deacetylation followed by reaction with chloro derivative **11 b** afforded α‐thioglycoside **15** in 48 % yield. Compound **11 b** was prepared from polyhydroxyalkylfuroate **10**,^21^ after oxidation with NaIO_4_ followed by reduction with NaBH_4_ and chlorination. Subsequent nucleophilic displacement with KSAc and deacetylation afforded thiol **12 b** in good overall yield, which after reaction with penta‐*O*‐acetyl‐α‐d‐galactopyranosyl bromide **14** exclusively gave β‐thioglycoside **16**. Oxidation of **15** and **16** with MCPBA (*meta*‐chloroperoxybenzoic acid) under different reaction conditions afforded sulfone derivatives **17** and **18** and sulfoxides **19** and **20**. Final deacetylation of compounds **15**–**20** under Zemplén conditions gave compounds **4**–**9** in 65–99 % yield.

The interactions of **4**–**9** with CTB were first studied by weak affinity chromatography (WAC),^30^ and the *K*_D_ values are shown in Table 1. The new ligands showed *K*_D_ values ranging from 0.35 mm to 0.48 mm, coming across as very promising CTB binders compared with **1**–**3**, for which the *K*_D_ values were in the low mm range.^21^

**Table 1:** *K*_D_ values for the binding of ligands **4**–**9** to CTB, as determined by WAC.

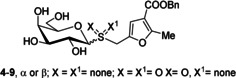			
Compound	Glycosidic bond	X, X^1^	*K*_D_ [mm]
**4**	β	none, none	0.35
**5**	α	none, none	0.26
**6**	β	O, O	0.34
**7**	α	O, O	0.34
**8**	β	O, none	0.45
**9**	α	O, none	0.48

STD NMR experiments were carried out to confirm the binding to CTB for all the members of the set **4**–**9**, and to get structural information on their binding modes.

**Figure 2:**
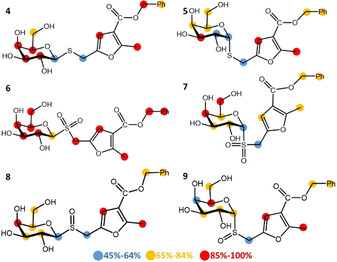
STD NMR binding epitope mapping of ligands **4**–**9**, based on the normalized STD values from the initial slope of each proton, relative to the most intense one (for which 100 % is arbitrarily assigned). Legend indicates weak (blue), medium (yellow), and strong (red) intensities (closer contacts with CTB). Proton assignment of the ligands, raw build‐up curves data, and normalized STD values are reported in the Supporting Information.

The determined binding group epitope mappings of their interactions with CTB showed a significant degree of variability among the six ligands (Figure 2), even at the common galactose ring. This was surprising, as the GM1 binding pocket of CTB provides a specific galactose subsite, which called for further investigation. To that aim, we resorted to our recently developed DEEP‐STD NMR method^8^ to elucidate the ligand orientation in the bound state.

### *“DEEP‐STD NMR fingerprinting”* characterizes the bound orientations of 4–7 in the GM1 binding pocket of CTB

DEEP‐STD NMR spectroscopy is a novel tool for revealing the bioactive orientation of ligands in a protein binding pocket, if the 3D geometry of the binding pocket is known.^8^ The availability of a crystal structure of the complex of CTB with a small galactoside derivative, 3‐nitrophenyl‐α‐d‐galactopyranoside, 3NPG (PDB ID: 1EEI),^31^ encouraged us to propose a novel method that we call “*DEEP‐STD NMR fingerprinting*”. First, the DEEP‐STD NMR pattern of 3NPG in complex with CTB is determined, providing DEEP‐STD factors, ΔSTD, which can be directly correlated with the orientation of 3NPG in the crystal structure (i.e., which ligand protons are close to a given type of amino acid side chain^8^). Then, the patterns of DEEP‐STD factors of the “unknown” ligands (**4**–**9**) are determined and compared with that of 3NPG. This approach, first, allows us to confirm the binding of unknown ligands to a known binding pocket and, second, it sheds light on their relative orientation, in comparison to the orientation of known ligands.

We carried out the DEEP‐STD NMR fingerprinting of the GM1 binding pocket of CTB with 3NPG^8^ and ligands **4**–**7** (ligands **8** and **9** were excluded owing to degradation over the time of the experiments). We irradiated the different samples containing the CTB/ligand complexes at 0.60 ppm and 2.25 ppm (on‐resonance frequencies), as already reported for the 3NPG/CTB complex.^8^ At 2.25 ppm, the glutamate and glutamine side chains in the galactose subsite are directly irradiated, and at 0.60 ppm the irradiation is on the aliphatic side chains.

**Figure 3:**
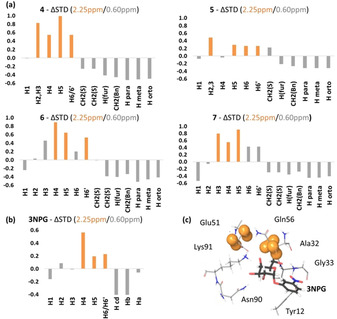
Differential epitope mapping ΔSTD (2.25 ppm/0.60 ppm) of a) ligands **4**–**7** and b) 3NPG in complex with CTB (reference sample), obtained at 2 s saturation time. For each ligand, the three largest positive ΔSTD values are shown as orange bars. c) Crystal structure of the complex 3NPG/CTB (PDB ID: 1EEI).^31^ Protein protons directly irradiated at 2.25 ppm are highlighted with an orange surface.

The determined ΔSTD (2.25 ppm/0.60 ppm) for each complex are shown in Figure 3 (see also the Supporting Information, Figure S2). The overall positive ΔSTD values at the galactose protons for all the ligands **4**–**7** (Figure 3 a) indicate that the thio‐galactose ring is allocated in the galactose binding subsite of the GM1 pocket, by comparison with the positive ΔSTDs observed for the 3NPG/CTB complex (Figure 3 b). Still, differences in ΔSTDs were noticeable for some galactose protons of **4**–**7**, suggesting some reorientation of the galactose moiety in the galactose subsite, which results from the chemical differences at the benzyl furoate side of the molecules, in good agreement with the experimental binding epitope data (Figure 2).

### Molecular docking: ligands 4–7 bind CTB by occupying the GM1 galactose and sialic acid subsites

**Figure 4:**
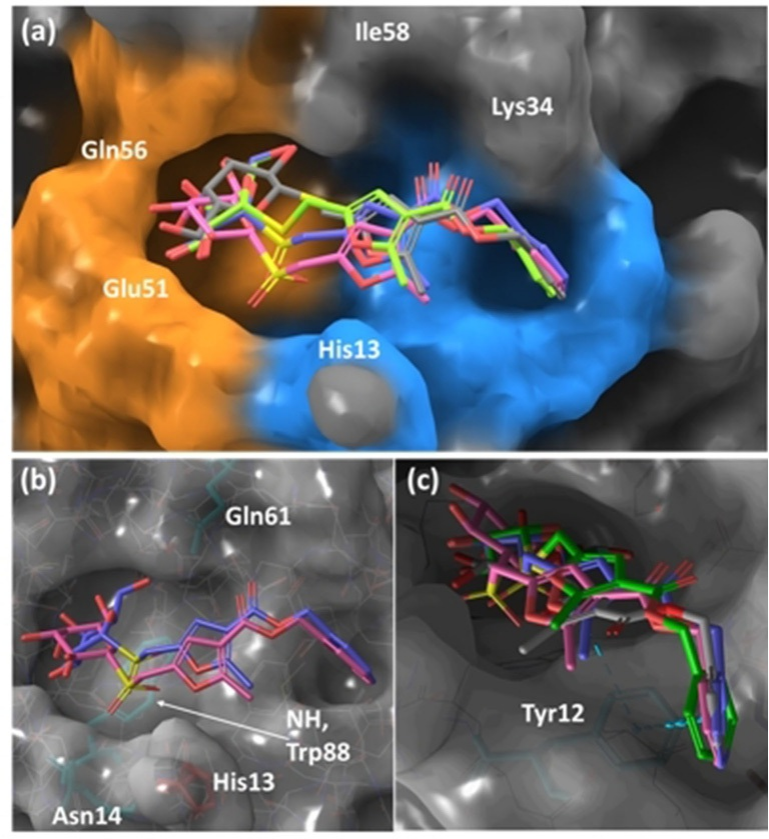
a) Lowest‐energy docking solutions for ligand **4** (gray), ligand **6** (pink), ligand **5** (green), and ligand **7** (violet) in complex with CTB. The residues lining the galactose and sialic acid binding subsites are highlighted with orange and blue surfaces, respectively. b) On a semi‐transparent surface, the sulfone oxygen atoms of **6** and **7** are shown pointing towards Asn14 and Trp88 (in turquoise sticks), and the methyl group on the furoate pointing parallel to His13 (red sticks), rather than towards Gln61 (turquoise sticks). c) Zoom on the phenyl moieties of ligands **4**–**7**, showing the interaction with Tyr12 (turquoise sticks).

We performed rigid molecular docking simulations of ligands **4**–**7** by using the crystal structure of CTB in the GM1/CTB complex (PDB ID: 3CHB).^32^ For each ligand, the lowest energy solutions converged to a binding mode in which the thio‐galactose moiety sits in the galactose binding subsite and the furoate‐benzyl tail lies in the sialic acid binding subsite within the GM1 binding pocket of CTB (Figure 4).

Remarkably, the galactose ring of the four ligands showed different orientations within the subsite, in good agreement with the experimental NMR observations (Figures 2 and 3). On the contrary, the benzyl groups of all the four ligands converged into a single orientation in the adjacent sialic acid subsite (blue surface in Figure 4 a), where a stabilizing π‐stacking with Tyr12 can take place (Figure 4 c).

These results agree with previous studies showing that CTB ligands with aromatic rings can efficiently π‐stack with Tyr12 in the sialic acid binding subsite.^31^, ^33^ On the other hand, ligands **4**–**7** fine‐tune the orientation of their thio‐galactose moieties within the binding subsite by favorable contacts with the amide group of Asn14 and/or the NH group of the side chain of Trp88 present at the protein surface (Figure 4 b). The impact on galactose orientation is substantial for the sulfone epimers, which can form hydrogen‐bonds with Asn14 and Trp88 through their two oxygens (Figure 4 b), disrupting the hydrogen‐bond network of the galactose ring in the natural ligand GM1 and altering its orientation.

Globally, the 3D molecular models of the complexes of CTB with ligands **4**–**7**, in agreement with the DEEP‐STD NMR fingerprinting approach, demonstrate that small galactoside‐based ligands armed with a benzyl furoate tail bind the GM1 pocket in CTB in a bidentate mode, simultaneously occupying the galactose and sialic acid subsites. Decorations at the glycosidic linkage have a sensitive impact on the binding mode of the galactose ring. This is relevant for the design of CTB inhibitors based on mimicking the GM1 galactose, as the sulfone affects the mimicry of the galactose orientation in the GM1 natural ligand.

How do larger ligands **2** and **3** occupy the GM1 binding pocket? STD NMR competition experiments suggest the presence of an unknown subsite. In the complexes of CTB with ligands **4**–**7**, the whole GM1 binding pocket is very efficiently occupied (Figure 4) with all parts of the relatively small ligand being intimately recognized by the protein (Figures 2 and 4). This result prompted the question of how a larger ligand, like **3**, is able to be accommodated in the pocket. Binding group epitope mappings of ligands **2** and **3** for their interactions with CTB have already been reported,^21^ supporting a bidentate mode of binding and showing that the spacer linking both ligand ends also establishes contacts with the protein (Supporting Information, Figure S1).^21^ Interestingly, ligand **2** and the PHF tail of ligand **3** showed similar binding epitope mappings, strongly suggesting that both bind into the same CTB subsite (Supporting Information, Figure S1).

Owing to the non‐carbohydrate nature of the PHF end present in ligands **2** and **3**, and their larger size, in comparison to ligands **4**–**7**, the question arises as to whether these ligands might be interacting in a binding site other than the sialic acid subsite of the GM1 pocket. Here, we tested this hypothesis and aimed to provide 3D models of the complexes of **2** and **3** with CTB, validated by NMR spectroscopy.

**Figure 5:**
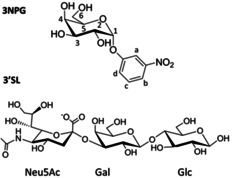
Chemical formulae for 3‐nitrophenyl α‐d‐galactopyranoside (3NPG; reporter for the Gal subsite) and 3′‐sialyllactose (3′SL; reporter Neu5Ac subsite).

We started by exploring the occupancy of the two (galactose/sialic acid) binding subsites of CTB by STD NMR competition experiments. To that aim, we selected STD‐active probe ligands known to occupy either subsite in the GM1 binding pocket of CTB (we call them subsite “reporters”) and probed whether any of the ligands **2** or **3** were able to displace any of the reporters in competition experiments. 3NPG was used as the galactose subsite reporter and 3′‐sialyllactose (3′SL), the Neu5Ac‐containing branch of GM1, as the reporter for the sialic acid subsite (Figure 5).

First (Table 2, experiment a), STD NMR signals of a sample containing ligand **3** and CTB were observed and its competition with the galactose subsite reporter 3NPG was monitored. In this experiment, 3NPG displaced ligand **3** when added to the solution in an equimolar amount (Supporting Information, Figure S3 a). Upon addition of 3NPG, the STD signals of ligand **3** were halved, in agreement with their similar *K*_D_ (3NPG 1.10 mm vs. ligand **3** 1.05 mm),^21^ confirming that the thio‐galactose moiety of ligand **3** binds to the galactose subsite within the GM1 binding pocket.

**Table 2:** Table of results of the competition experiments (for the STD spectra, see the Supporting Information, Figure S3).

Experiment	Ligand	Competitor	Displacement
a)	**3**	3NPG	Π
b)	**3**	**2**	Π
c)	**2**	3NPG	Ο
d)	3′SL	**2**	Ο

After that, we probed the competition between ligands **2** and **3**, so ligand **2** was added at an equimolar amount to a sample of ligand **3** in complex with CTB (Table 2, experiment b). In this case, the STD signals of ligand **3** dropped upon addition of ligand **2** (Supporting Information, Figure S3 b), which has a *K*_D_=1.35 mm, 21 proving that both ligands **3** and **2** occupy a common subsite, most likely through their common PHF tail.

Then, ligand **2** was tested in competition with 3NPG (Table 2, experiment c). No competition was observed in this case, showing that the binding of ligand **2** to CTB does not involve the galactose subsite (Supporting Information, Figure S3 c).

Finally, to investigate if the PHF tail binds to CTB in the sialic acid subsite, the competition experiment between 3′SL and ligand **2** was carried out (Table 2, experiment d). Binding of 3′SL was detected (Supporting Information, Figure S5), but the STD signals of 3′SL were not affected by the addition of ligand **2** (Supporting Information, Figure S3 d). As a control, adding 3′SL to a sample containing CTB and ligand **2**, did not affect the signals of **2** either (Supporting Information, Figure S3 e), confirming that binding of ligands **3** and **2** does not involve the sialic acid subsite. Globally, the STD NMR competition experiments strongly support the existence of an unknown subsite on CTB, which is able to accommodate the PHF tails in ligands **2** and **3**.

### ILOE experiments exploring subsites proximity in the GM1 binding pocket of CTB

**Figure 6:**
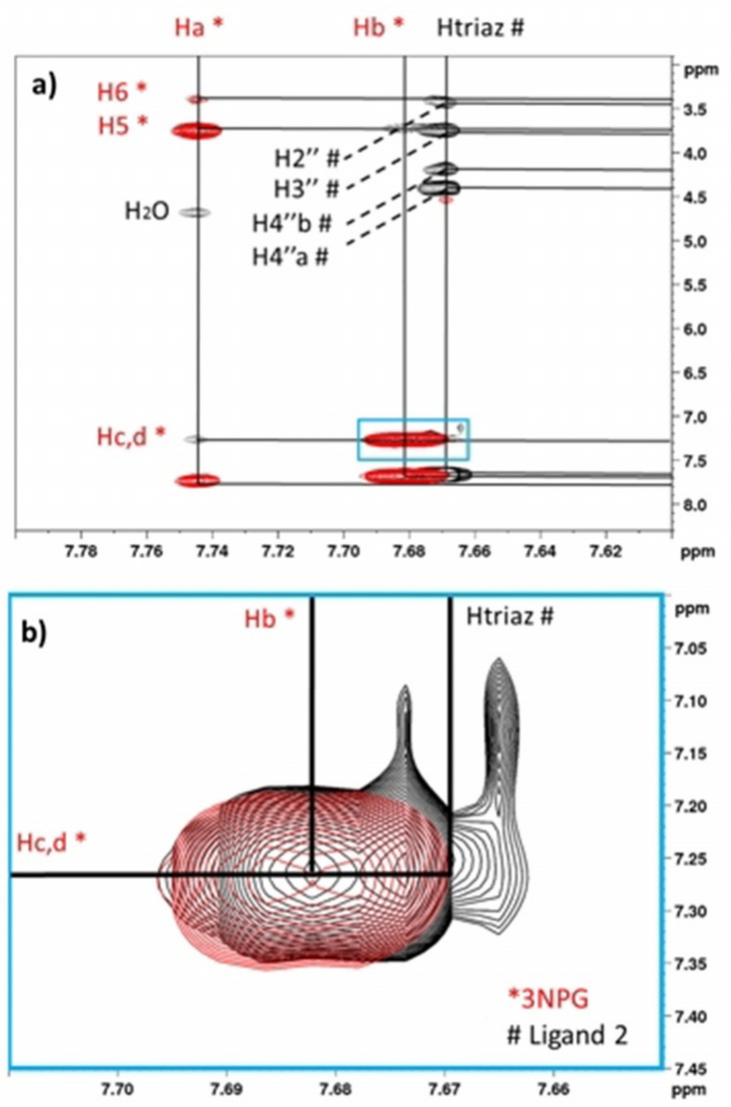
Top) Expansion of the superposition of tr‐NOESY experiments of the **2**/3NPG/CTB ternary complex (in black) and of the 3NPG/CTB control complex (in red). Diagonal and cross peaks are assigned and the ILOE peak correlating H_c,d_ of 3NPG and H_triaz_ of ligand **2** are highlighted in blue. Bottom) Magnification of the ILOE cross peak. Signals belonging to ligand **2** and 3NPG are marked with a red asterisk and a black hashtag, respectively.

The scenario depicted by the STD NMR competition experiments did not exclude that ligands **3** and **2** could compete for a subsite outside the GM1 binding pocket. On the other hand, as previously observed (experiment c, Table 2), 3NPG and ligand **2** can bind simultaneously to CTB. On this basis, we decided to test experimentally if they bind in proximity to each other (adjacent binding subsites) by generating the **2**/CTB/3NPG ternary complex in solution and resorting to an ILOE (tr‐NOESY) experiment^23^ (Figure 6).

In the tr‐NOESY spectrum of the ternary complex, we did observe an *inter*‐ligand **2**–3NPG cross peak between the triazole proton (H_triaz_) of ligand **2** (7.67 ppm) and the H_c,d_ protons of 3NPG (positions *meta* and *para* to the nitro group, both at 7.27 ppm), along with the expected intra‐ligand cross peaks for both ligands. As a control, to confirm the inter‐ligand nature of that NOE, the same tr‐NOESY experiment was performed on the binary 3NPG/CTB and ligand **2**/CTB complexes as well as on CTB alone (see the Supporting Information, Figures S5 and S6 for control and full spectral width spectra of the ternary complex). The 7.67 ppm/7.27 ppm cross peak was absent in the three control spectra, confirming that the cross peak is not due to any protein–ligand or protein–protein NOE, but it genuinely reports a close contact (ILOE) between the triazole moiety of ligand **2** and the nitrophenyl group of 3NPG in the bound state.

The observation of this *inter*‐ligand **2**–3NPG NOE in the state bound to CTB is of paramount importance in our study, as it proves the existence of a subsite accommodating the furoate‐benzyl chain, adjacent to the galactose subsite, but different to the sialic acid subsite. To generate 3D models of the complexes with **2** and **3**, we then focused our structural search on the proximities of the GM1 binding pocket. Furthermore, the observed ILOE gave us information on the orientation of ligand **2** in this novel subsite, which is such that its triazole is in proximity of the galactose subsite.

Hamiltonian replica exchange molecular dynamics (HREMD) reveals a novel subsite in the GM1 binding pocket that accommodates the polyhydroxyalkylfuroate tail of **2** and **3**. In contrast to the smaller ligands **4**–**7**, docking calculations of ligands **2** and **3** into the GM1 binding pocket of CTB failed to generate 3D models that could agree with the experimental data (see the Supporting Information). STD predictions from the docking models were obtained by using CORCEMA‐ST.^28^ Predicted STD intensities are compared with experimental NMR data through the so‐called NOE *R*‐factor, with a low value indicating a good matching. In our experience, values ≤0.3 report good agreement between the 3D model and the experimental data. The PHF tail of **3** was predicted to occupy the sialic acid subsite, in disagreement with STD NMR competition experiments and binding epitopes, as quantitated by the high NOE *R*‐factor (Supporting Information, Figure S11). Similarly, docking solutions for ligand **2** also showed high NOE *R*‐factors (Supporting Information, Figure S12).

Taking into account the ILOE results, we then decided to investigate the dynamics of the GM1 binding pocket in CTB to probe the flexibility of side chains in the vicinity of the galactose and sialic acid subsites. To that aim, a 100 ns molecular dynamics (MD) simulation of free CTB was performed. To enhance sampling of the free state, an HREMD‐based method was chosen in which the non‐bonding interaction between the solvent and hydrophobic regions of the protein were modified in each replica, which has previously been shown to be effective in identifying druggable transient binding sites.^27^

**Figure 7:**
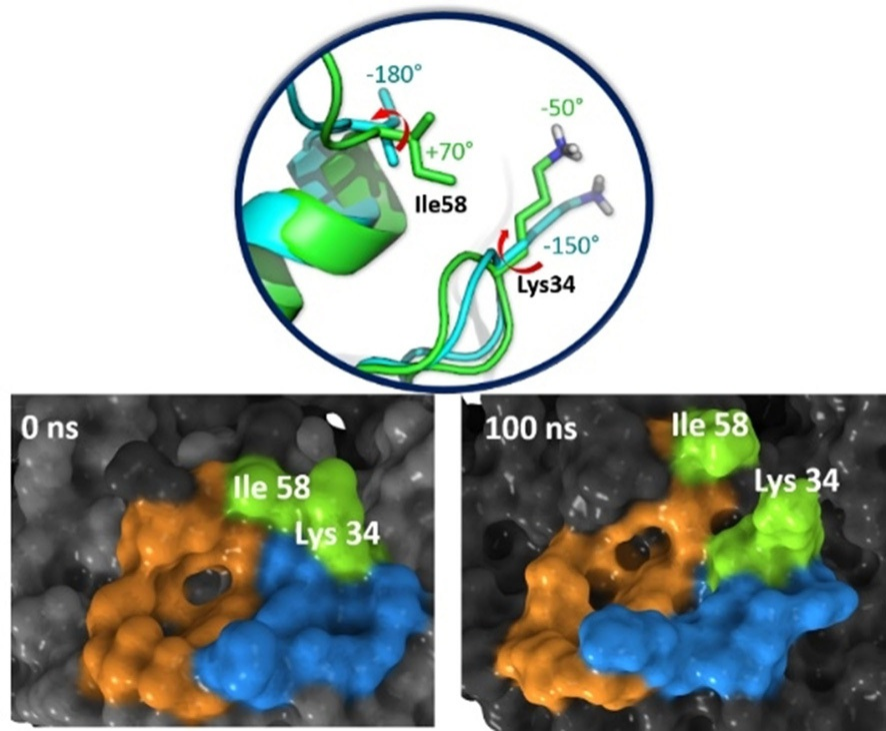
Opening of a novel subsite in the GM1 binding pocket of CTB. Top) In green, the conformations of the side chains of Ile58 and Lys34 in the crystal structure of CTB (PDB ID:3CHB); in turquoise, overlaid, the open state of the novel subsite from HREMD simulations. The red arrows indicate the χ1 torsional rotation from closed to open states for the two residues involved (χ1 values reported for both). Bottom) Surface representation of the closed (left) and open conformations (right), corresponding to initial and final frames of the HRMED apo CTB simulation. Galactose binding subsite in orange; sialic acid binding subsite in blue; Ile58 and Lys34 in green and labeled.

Analysis of the trajectory over all five subunits of the CTB pentamer revealed the existence of a transient subsite close to the galactose and sialic acid subsites (Figure 7 and Supporting Information, Figure S13). Although this subsite is closed in the crystal structure, opening is facilitated by rotation of the Ile59 and Lys138 side chains (Figure 7). As no main chain rearrangement is observed, the energy barrier between the ‘closed’ and ‘open’ conformations is expected to be minimal such that both states are readily sampled in the unbound protein.

Furthermore, repeating the simulation with CTB bound to GM1 revealed that opening of this cryptic subsite still takes place in the presence of the native ligand (Supporting Information, Figures S14 and S15). This shows that this subsite is accessible even when the galactose and sialic acid subsites are engaged in binding, opening the door to further CTB inhibitor design that could exploit all three subsites.

**Figure 8:**
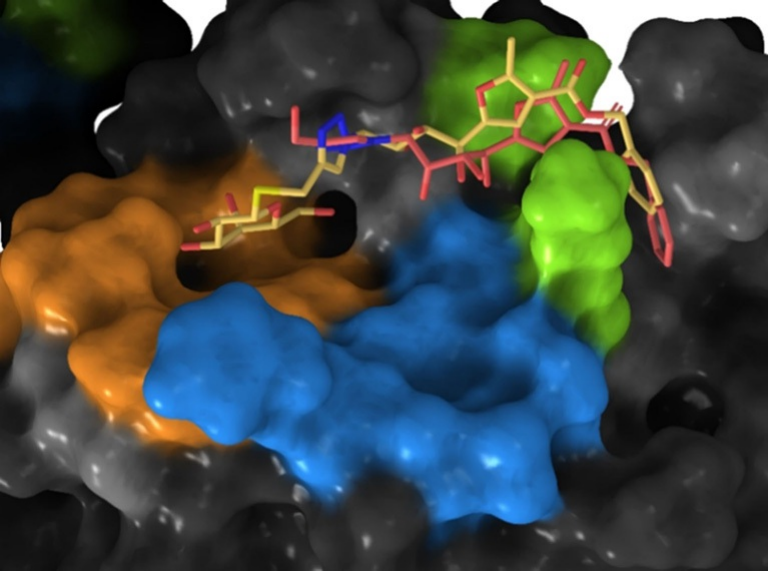
Superposition of the representative MD frames of the most populated clusters of the complexes of CTB with ligand **3** (pale yellow) and ligand **2** (light red) bound to CTB in the “open” conformation. The galactose subsite is in orange and the sialic acid subsite is in blue; Lys34 and Ile58, defining the novel subsite, in green.

We then carried out docking calculations of ligand **3** to this ‘open’ conformation. The best docking solutions showed indeed that the PHF moiety occupies the novel subsite, in addition to good convergence at the galactose end towards the galactose subsite (Figure 8, ligand **3** in yellow). Furthermore, a long MD simulation of this complex indicated that this conformation is dynamically stable, and, most importantly, the novel subsite is occupied for most of the simulation (Supporting Information, Figures S16 and S17).

The same approach was followed for ligand **2** to generate a 3D model of ligand **2** bound in the open conformation of CTB (Figure 8, ligand **2** in light red). Long MD simulation revealed even greater dynamic stability than the **3**/CTB complex, with the furoate‐benzyl chain exclusively occupying the novel subsite throughout the simulation. Without a galactose moiety to act as an anchor, ligand **2** buries itself further into the hydrophobic binding subsite (Figure 8). Moreover, the basic side chain of Lys138 is able to form several favorable hydrogen‐bonding interactions with the furoate‐benzyl moiety, which are not possible for ligand **3**.

Finally, the 3D models of the complexes were validated against the experimental NMR data by using CORCEMA‐ST. Instead of considering a single docking solution, STD NMR intensities were predicted running CORCEMA‐ST on a set of 100 frames from each of the MD simulations of the complexes of ligands **2** and **3** with CTB, to reflect the flexibility of the systems. The agreement with the experimental data was very good, with NOE *R*‐factors of 0.25 and 0.31, respectively (Supporting Information, Figures S18 and S19). These results confirm that, in solution, the novel binding subsite in the GM1 binding pocket of CTB is populating the open state and can accommodate the PHF tail in the bound state to ligands **2** and **3**.

## Conclusion

In this work, we have shown that our novel DEEP‐STD NMR fingerprinting approach, combined with MD simulations, is a very powerful tool to explore the multi‐subsite architecture of protein binding pockets. Here, we have applied it to an important drug target, that is, cholera toxin subunit B (CTB), but the approach is expected to be of broad applicability in the investigation of ligand binding to other multi‐subsite relevant targets.

To explore the binding subsites of CTB, six novel thio‐galactosidic CTB inhibitors (**4**–**9**) were synthesized and studied. These showed a much higher affinity for CTB, relative to their precursors.^21^ Structural analysis revealed that, for the six ligands, the aromatic moiety sits in the sialic acid binding subsite of GM1, π‐stacking with the underlying side chain of Tyr12. On the other hand, the decoration at the thio‐glycosidic linkage and the variability of the anomeric configuration disrupt the mimicry of the galactose ring in the binding subsite of GM1.

For the larger ligands **2** and **3**,^21^ a combination of STD NMR competition and ILOE experiments, as well as docking and Hamiltonian replica exchange molecular dynamics (HREMD) allowed us to unveil the existence of a novel cryptic binding subsite on the GM1 binding pocket of CTB. This subsite was not present in the rigid crystal structure of CTB bound to GM1, but molecular dynamics revealed that it can be readily formed by rearrangements in the side chains of Ile59 and Lys138, which are adjacent to the known galactose and sialic acid binding subsites at the GM1 binding pocket. 3D models of the complexes, generated by MD simulations combined with STD NMR spectroscopy and validated by using CORCEMA‐ST, demonstrated that ligands **2** and **3** occupy this subsite with their polyhydroxyalkylfuroate tails. The existence of this new non‐carbohydrate binding subsite in CTB, accessible even when the two known subsites are occupied, paves the way for future design of three‐finger thio‐galactose mimetics as potentials CTB inhibitors.

## Experimental Section

### Materials

3‐Nitrophenyl α‐d‐galactopyranoside (3NPG), cholera toxin subunit B, deuterium oxide (99.9 % ^2^H), dimethyl sulfoxide‐d6 ([D_6_]DMSO), disodium hydrogen phosphate (Na_2_HPO_4_), potassium dihydrogen phosphate (KH_2_PO_4_), sodium chloride (NaCl), and potassium chloride (KCl) were purchased from Sigma–Aldrich. Ligands were prepared in stock solutions 3 % [D_6_]DMSO.

### Synthesis and characterization. General

Optical rotations were measured in a Jasco P‐2000 spectropolarimeter in a 1.0 cm or 1.0 dm tube (Na, λ 598 nm). Infrared spectra were recorded with a Jasco FTIR‐410 spectrophotometer.  δ are given in ppm and J in Hz. J are assigned and not repeated. All the assignments were confirmed by 2D spectra (COSY and HSCQ). High resolution mass spectra were recorded on a Q‐Exactive spectrometer. TLC was performed on silica gel 60 F_254_ (Merck), with detection by UV light charring with H_2_SO_4_ or with reagent [(NH_4_)_6_MoO_4_, Ce(SO_4_)_2_, H_2_SO_4_, H_2_O]. Silica gel 60 (Merck, 40‐60 and 63‐200 μm) was used for preparative chromatography.

### Synthesis of ligands 4–9

**((4‐Benzyloxycarbonyl‐5‐methylfuran‐2‐yl)methyl)‐1‐thio‐β‐d‐galactopyranoside (4)**: To a solution of **16** (40 mg, 0.066 mmol) in anhydrous MeOH (2 mL), NaOMe (13 μL, 0.5 m in MeOH) was added and the mixture stirred at 0 °C for 2 h. Then, the mixture was neutralized with Amberlite IR‐120H^+^, filtered, and washed with MeOH. The filtered solution was concentrated to give pure **4** (26 mg, 0.061 mmol, 92 %) as a pale‐yellow oil. ${\left[\alpha \right]}_{D}^{23}$ =−104.8 (*c*=0.64, MeOH); IR: ῡ=3381 (OH), 2907, 1705 (C=O), 1072 cm^−1^; ^1^H NMR (300 MHz, CD_3_OD): *δ*=7.43–7.31 (m, 5 H, H‐Ar), 6.52 (s, 1 H, H‐3′), 5.26 (s, 2 H, C*H*_2_Ph), 4.28 (d, 1 H, *J*_1,2_=9.6 Hz, H‐1), 4.03 (d, 1 H, ^2^ *J*_H,H_=14.5 Hz, C*H*_2_S), 3.87 (dd, 1 H, *J*_4,3_=3.2, *J*_4,5_=0.6 Hz, H‐4), 3.78 (d, 1 H, ^2^ *J*_H,H_=14.5 Hz, C*H*_2_S), 3.76 (dd, 1 H, ^2^ *J*_6a,6b_=11.5, *J*_6a,5_=7.1 Hz, H‐6a), 3.68 (dd, 1 H, *J*_6b,5_=5.1 Hz, H‐6b), 3.57 (t, 1 H, *J*_2,3_=9.5 Hz, H‐2), 3.51–3.47 (m, 1 H, H‐5), 3.41 (dd, 1 H, H‐3), 2.52 ppm (s, 3 H, Me); ^13^C NMR (75.4 MHz, CD_3_OD): *δ*=165.2 (*C*OOBn), 160.4, 151.4 (C‐2′, C‐5′), 137.7 (C_q_‐Ar), 129.6, 129.2, 129.1 (C‐Ar), 115.0 (C‐4′), 109.3 (C‐3′), 86.1 (C‐1), 80.8 (C‐5), 76.3 (C‐3), 71.4 (C‐2), 70.5 (C‐4), 67.0 (*C*H_2_Ph), 62.7 (C‐6), 26.2 (*C*H_2_S), 13.9 ppm (Me); HRESIMS: *m*/*z* found: 447.1079; calcd For C_20_H_24_O_8_NaS (*M*+Na)^+^: 447.1084.

**((4‐Benzyloxycarbonyl‐5‐methylfuran‐2‐yl)methyl)‐1‐thio‐α‐d‐galactopyranoside (5)**: To a solution of **15** (47 mg, 0.079 mmol) in anhydrous MeOH (1.5 mL), NaOMe (16 μL, 0.5 m in MeOH) was added and the mixture stirred at 0 °C for 1.5 h. Then, the mixture was neutralized with Amberlite IR‐120H^+^, filtered, and washed with MeOH. The filtered solution was concentrated to give pure **5** (34 mg, 0.079, quantitative) as a white solid. ${\left[\alpha \right]}_{D}^{25}$ =249.8 (*c*=0.68, MeOH); IR: ῡ=3382 (OH), 2927, 1708 (C=O), 1069 cm^−1^; ^1^H NMR (300 MHz, CD_3_OD): *δ*=7.43–7.29 (m, 5 H, H‐Ar), 6.50 (s, 1 H, H‐3′), 5.35 (d, 1 H, *J*_1,2_=5.7 Hz, H‐1), 5.25 (s, 2 H, C*H*_2_Ph), 4.16 (t, 1 H, *J*_5,6_=6.2 Hz, H‐5), 4.09 (dd, 1 H, *J*_2,3_=10.0 Hz, H‐2), 3.89 (d, 1 H, *J*_4,3_=2.4 Hz, H‐4), 3.82 (d, 1 H, ^2^ *J*_H,H_=14.9 Hz, C*H*_2_S), 3.76–3.68 (m, 2 H, H‐6), 3.65–3.59 (m, 2 H, H‐3, C*H*_2_S), 2.52 ppm (s, 3 H, Me); ^13^C NMR (75.4 MHz, CD_3_OD): *δ*=165.2 (*C*OOBn), 160.4, 151.4 (C‐2′, C‐5′), 137.7 (C_q_‐Ar), 129.6, 129.21, 129.19 (C‐Ar), 115.0 (C‐4′), 109.1 (C‐3′), 85.8 (C‐1), 73.0 (C‐5), 72.3 (C‐3), 70.9 (C‐4), 69.5 (C‐2), 67.0 (*C*H_2_Ph), 62.7 (C‐6), 25.2 (*C*H_2_S), 13.8 ppm (Me); HRESIMS: *m*/*z* found: 447.1072; calcd For C_20_H_24_O_8_NaS (*M*+Na)^+^: 447.1084.

**((4‐Benzyloxycarbonyl‐5‐methylfuran‐2‐yl)methyl)‐1‐sulfonyl‐β‐d‐galactopyranoside (6)**: To a solution of **18** (60 mg, 0.096 mmol) in anhydrous MeOH (2 mL), NaOMe (20 μL, 0.5 m in MeOH) was added and the mixture stirred at 0 °C for 1 h. Then, the mixture was neutralized with Amberlite IR‐120H^+^, filtered, and washed with MeOH. The filtered solution was concentrated and the residue was purified by column chromatography on silica gel (CH_2_Cl_2_/MeOH 10:1→8:1) to give **6** (33 mg, 0.072 mmol, 75 %) as a white solid. ${\left[\alpha \right]}_{D}^{23}$ =−25.0 (*c*=0.84, MeOH); IR: ῡ=3382 (OH), 2920, 1711 (C=O), 1082 cm^−1^; ^1^H NMR (300 MHz, CD_3_OD): *δ*=7.44–7.29 (m, 5 H, H‐Ar), 6.86 (s, 1 H, H‐3′), 5.28 (s, 2 H, C*H*_2_Ph), 4.76 (d, 1 H, ^2^ *J*_H,H_=15.0 Hz, C*H*_2_S), 4.49 (d, 1 H, C*H*_2_S), 4.33 (d, 1 H, *J*_1,2_=9.5 Hz, H‐1), 4.12 (t, 1 H, *J*_2,3_=*J*_2,1_=9.4 Hz, H‐2), 3.89–3.81 (m, 2 H, H‐4, H‐6a), 3.75–3.64 (m, 2 H, H‐5, H‐6b), 3.55 (dd, 1 H, *J*_3,4_=3.3 Hz, H‐3), 2.56 ppm (s, 3 H, Me); ^13^C NMR (75.4 MHz, CD_3_OD): *δ*=164.8 (*C*OOBn), 162.0, 142.3 (C‐2′, C‐5′), 137.7 (C_q_‐Ar), 129.6, 129.3, 129.2 (C‐Ar), 115.9 (C‐4′), 114.2 (C‐3′), 91.2 (C‐1), 82.2 (C‐5), 75.7 (C‐3), 70.2 (C‐4), 67.5 (C‐2), 67.1 (*C*H_2_Ph), 62.8 (C‐6), 50.9 (*C*H_2_S), 13.9 ppm (Me); HRESIMS: *m*/*z* found 479.0976; calcd For C_20_H_24_O_10_NaS (*M*+Na)^+^: 479.0982.

**((4‐Benzyloxycarbonyl‐5‐methylfuran‐2‐yl)methyl)‐1‐sulfonyl‐α‐d‐galactopyranoside (7)**: To a solution of **17** (56 mg, 0.090 mmol) in anhydrous MeOH (1.5 mL), NaOMe (18 μL, 0.5 m in MeOH) was added and the mixture stirred at 0 °C for 2 h. Then, the mixture was neutralized with Amberlite IR‐120H^+^, filtered, and washed with MeOH. The filtered solution was concentrated to give pure **7** (32 mg, 0.070 mmol, 78 %) as a white solid. ${\left[\alpha \right]}_{D}^{26}$ =91.8 (*c*=0.98, MeOH); IR: ῡ=3382 (OH), 2925, 1713 (C=O), 1080 cm^−1^; ^1^H NMR (300 MHz, CD_3_OD): *δ*=7.43–7.29 (m, 5 H, H‐Ar), 6.80 (s, 1 H, H‐3′), 5.27 (s, 2 H, C*H*_2_Ph), 5.09 (d, 1 H, *J*_1,2_=6.0 Hz, H‐1), 4.70 (d, 1 H, ^2^ *J*_H,H_=14.9 Hz, C*H*_2_S), 4.50 (d, 1 H, C*H*_2_S), 4.37–4.24 (m, 3 H, H‐2, H‐3, H‐4), 4.00–3.99 (m, 1 H, H‐5), 3.82 (dd, 1 H, ^2^ *J*_6a,6b_=11.8, *J*_6a,5_=7.3 Hz, H‐6a), 3.71 (dd, 1 H, *J*_6b,5_=4.4 Hz, H‐6b), 2.55 ppm (s, 3 H, Me); ^13^C NMR (75.4 MHz, CD_3_OD): *δ*=164.7 (*C*OOBn), 161.9, 142.6 (C‐2′, C‐5′), 137.6 (C_q_‐Ar), 129.6, 129.3, 129.2 (C‐Ar), 115.8 (C‐4′), 114.0 (C‐3′), 90.9 (C‐1), 78.7, 70.7, 69.2 (C‐2, C‐3, C‐4), 70.0 (C‐5), 67.1 (*C*H_2_Ph), 62.6 (C‐6), 52.8 (*C*H_2_S), 13.9 ppm (Me); HRESIMS: *m*/*z* found: 479.0971; calcd For C_20_H_24_O_10_NaS (*M*+Na)^+^: 479.0982.

**((4‐Benzyloxycarbonyl‐5‐methylfuran‐2‐yl)methyl)‐1‐sulfinyl‐β‐d‐galactopyranoside (8)**: To a −78 °C solution of **16** (142 mg, 0.24 mmol) in CH_2_Cl_2_ (5 mL), MCPBA (59 mg, 0.24 mmol) was added and the mixture stirred at −78 °C→−30 °C for 3 h. Then, a sat. aq. solution of NaHCO_3_ was added and the mixture was extracted with CH_2_Cl_2_. The combined organic phases were washed with a sat. aq. solution of NaHCO_3_ and brine, dried over Na_2_SO_4_, filtered, and evaporated. The resulting residue was purified by column chromatography on silica gel (EtOAc/cyclohexane 1:1→1:1) to give **20** (100 mg, 0.164 mmol, 68 %) as a white solid. To a solution of this compound (60 mg, 0.099 mmol) in anhydrous MeOH (2 mL), NaOMe (20 μL, 0.5 m in MeOH) was added and the mixture stirred at 0 °C for 1 h. Then, the mixture was neutralized with Amberlite IR‐120H^+^, filtered, and washed with MeOH. The filtered solution was concentrated and the residue was purified by column chromatography on silica gel (CH_2_Cl_2_/MeOH 10:1→8:1) to give **8** (28 mg, 0.064 mmol, 65 %) as a white solid. ${\left[\alpha \right]}_{D}^{27}$ =−73.5 (*c*=0.82, MeOH); IR: ῡ=3341 (OH), 2929, 1713 (C=O), 1072 cm^−1^; ^1^H NMR (300 MHz, CD_3_OD, 1.5:1 mixture of diastereoisomers A/B): *δ*=7.43–7.28 (m, H‐Ar(A), H‐Ar(B)), 6.76, (s, H‐3′(B)), 6.75 (s, H‐3′(A)), 5.31–5.23 (m, C*H*_2_Ph(A), C*H*_2_Ph(B)), 4.42–4.33 (m, H‐1(A), C*H*_2_S(A), C*H*_2_S(B)), 4.03–3.55 (m, H‐1(B), H‐2(A), H‐2(B), H‐3(A), H‐3(B), H‐4(A), H‐4(B), H‐5(A), H‐5(B), H‐6(A), H‐6(B)), 2.56 ppm (s, Me(A)) 2.55 (s, Me(B)); ^13^C NMR (75.4 MHz, CD_3_OD, mixture of diastereoisomers): *δ*=164.8, 164.7 (*C*OOBn(A), *C*OOBn(B)), 161.83, 161.77, 144.7 (C‐2′(A), C‐2′(B), C‐5′(A), C‐5′(B)), 137.7 (C_q_‐Ar), 129.6, 129.3, 129.2 (C‐Ar), 115.8 (C‐4′), 113.4, 113.2 (C‐3′(A), C‐3′(B)), 93.8 (C‐1(A)), 90.8 (C‐1(B)), 82.4, 82.3, 76.3, 76.1, 70.8, 70.3, 68.2, 66.5, (C‐2(A), C‐2(B), C‐3(A), C‐3(B), C‐4(A), C‐4(B), C‐5(A), C‐5(B)), 67.1 (*C*H_2_Ph), 62.9, 62.8 (C‐6(A), C‐6(B)), 46.6, 46.1 (*C*H_2_S(A), *C*H_2_S(B)), 13.9 ppm (Me); HRESIMS: *m*/*z* found: 463.1024; calcd for C_20_H_24_O_9_NaS (*M*+Na)^+^: 463.1033.

**((4‐Benzyloxycarbonyl‐5‐methylfuran‐2‐yl)methyl)‐1‐sulfinyl‐α‐d‐galactopyranoside (9)**: To a −78 °C solution of **15** (100 mg, 0.169 mmol) in CH_2_Cl_2_ (3.5 mL), MCPBA (42 mg, 0.17 mmol) was added and the mixture stirred at −78 °C→−30 °C for 2 h. Then, a sat. aq. solution of NaHCO_3_ was added and the mixture was extracted with CH_2_Cl_2_. The combined organic phases were washed with a sat. aq. solution of NaHCO_3_, water, and brine, dried over Na_2_SO_4_, filtered, and evaporated. The resulting residue was purified by column chromatography on silica gel (Et_2_O/cyclohexane 4:1→3:1) to give **19** (66 mg, 0.11 mmol, 65 %) as a white solid. To a solution of this compound (20 mg, 0.034 mmol) in anhydrous MeOH (0.7 mL), NaOMe (7 μL, 0.5 m in MeOH) was added and the mixture stirred at 0 °C for 1 h. Then, the mixture was neutralized with Amberlite IR‐120H^+^, filtered, and washed with MeOH. The filtered solution was concentrated and the residue was purified by column chromatography on silica gel (EtOAc/MeOH 5:1) to give **9** (11 mg, 0.025 mmol, 74 %) as a pale‐yellow oil. ${\left[\alpha \right]}_{D}^{27}$ =195.5 (*c*=0.67, MeOH); IR: ῡ=3369 (OH), 2923, 1710 (C=O), 1079 cm^−1^; ^1^H NMR (300 MHz, CD_3_OD, single diastereoisomer): *δ*=7.43–7.29 (m, 5 H, H‐Ar), 6.78 (s, 1 H, H‐3′), 5.27 (s, 2 H, C*H*_2_Ph), 4.75 (d, 1 H, *J*_1,2_=4.2 Hz, H‐1), 4.44 (d, 1 H, ^2^ *J*_H,H_=14.5 Hz, C*H*_2_SO), 4.28 (dd, 1 H, *J*_2,3_=7.7 Hz, H‐2), 4.24 (d, 1 H, C*H*_2_SO), 4.07–4.05 (m, 1 H, H‐5), 4.00 (dd, 1 H, *J*_3,4_=3.3 Hz, H‐3), 3.96–3.88 (m, 2 H, H‐4, H‐6a), 3.76–3.68 (m, 1 H, H‐6b), 2.56 ppm (s, 3 H, Me); ^13^C NMR (75.4 MHz, CD_3_OD, single diastereoisomer): *δ*=164.8 (*C*OOBn), 161.8, 144.6 (C‐2′, C‐5′), 137.7 (C_q_‐Ar), 129.6, 129.22, 129.19 (C‐Ar), 115.7 (C‐4′), 113.5 (C‐3′), 92.0 (C‐1), 80.3 (C‐4), 72.3 (C‐3), 69.9 (C‐2), 68.8 (C‐5), 67.1 (*C*H_2_Ph), 61.5 (C‐6), 47.8 (*C*H_2_SO), 13.9 ppm (Me); HRESIMS: *m*/*z* found: 463.1024; calcd For C_20_H_24_O_9_NaS (*M*+Na)^+^: 463.1033.

### Weak affinity chromatography

Recombinant CTB (SBL vaccines, Stockholm, Sweden) was immobilized onto Nucleosil silica (10 μm, 300 Å) and packed into a 50×2.1 mm column. The number of active groups of CTB was estimated to be 261 nmol. All chromatographic experiments were performed with an Agilent‐1100/Agilent‐1200 HPLC system. The mobile phase was 10 mm sodium phosphate, 0.15 mm sodium chloride, pH 7. The flow rate was 0.1 mL min^−1^ and the temperature 22 °C. The sample volume was 5 μL and the sample concentration 35 μm. Detection was performed at 220 nm. The retention factor (*k*′) and the affinity (*K*_D_) of the derivatives were calculated as described previously,^21^, ^30^ by using 3NPG as the reference compound (*K*_D_=1.06±0.04 mm).

### Nuclear magnetic resonance spectroscopy

Ligands **4**–**9** were assigned by performing COSY, TOCSY, and HSQC experiments on a 2 mm solution of the ligands dissolved in D_2_O, [D_6_]DMSO 5 %. For the STD NMR build‐up curves, sample containing 1 mm substrate (ligands **4**–**9**) and 5 μm CTB (each pentamer contains 5 equivalent binding sites), that is, protein/ligand ([P]/[L]) ratio of 1:40, were prepared. The build‐up curves were acquired with irradiation frequency 0.60 ppm, at 0.5, 1, 2, 3, 4, and 5 s, with a delay between experiment of 5 s and 256 scans. For the DEEP‐STD NMR fingerprinting approach and on‐resonance scanning of 3NPG and ligands **4**–**7**, STD NMR experiments at 2 s saturation time and 32 scans were performed at the irradiation frequencies of 0.60 ppm and 2.25 ppm.

The STD NMR competitions experiments were performed on samples constituted of 1 mm ligand and 5 μm CTB (each pentamer contains 5 equivalent binding sites), that is, protein/ligand ([P]/[L]) ratio of 1:40. First, the STD NMR experiment of the ligand under investigation (ligand **2** or **3**) was performed (saturation time 2 s, delay between experiments 5 s and 512 scans; the competitor ligand in equimolar concentration was freeze dried and added to the sample, then the experiment was repeated. Exchange‐transferred‐NOESY (tr‐NOESY) experiments were performed with [P]/[L]=1:10 on the ternary complex of ligand **3** and 3NPG with CTB (1 mm of each ligand and 20 μm CTB), with mixing time 1.2 s, delay 2 s, and 160 scans. As a control, the same experiment was performed on 3NPG/CTB, ligand **3**/CTB, and CTB alone in the same conditions. All the NMR experiments were performed at 278 K.

An STD pulse sequence that included 2.5 ms and 5 ms trim pulses and a 3 ms spoil gradient and water suppression by excitation sculpting with gradients was used (*stddiffesgp.3*). Saturation was achieved by applying a train of 50 ms Gaussian pulses (0.40 mW) on the f2 channel, at 0 ppm or 7.27 ppm (on‐resonance experiments) and 40 ppm (off‐resonance experiments). The broad protein signals were removed by using a 40 ms spinlock (*T*_1ρ_) filter (*stddiff.3*). Tr‐NOESY experiments were performed by using a phase‐sensitive pulse program with gradient pulses in the mixing time and a relaxation delay of 2 s, with water suppression using a 3–9–19 pulse sequence with gradients (*noesyfpgpph19*).^34^, ^35^, ^36^ All the experiments were recorded at ^1^H frequency of 800.23 MHz with a Bruker Avance III spectrometer equipped with a 5‐mmD probe TXI 800 MHz H‐C/N‐D‐05 Z BTO.

### Protein–ligand docking calculations

All molecular modeling was performed with the module Glide within Schrödinger's Maestro modeling suite.^24^, ^25^, ^26^ Coordinates for CTB were obtained from the Protein Data Bank (3CHB) for the closed conformation and from a representative frame of our HREMD trajectory of the GM1/CTB complex for the open conformation. Where necessary, coordinates for missing atoms were added according to known protein chemistry and side chain protonation was optimized for neutral pH. A short energy minimization was run by using the OPLS3 force field, converging heavy atoms to a RMSD of 0.3 Å. The receptor grid was then calculated, centering on the centroid of GM1 and with a length of 30 Å to encompass all subsites. Three‐dimensional structures of all ligands were generated by using a conformational search, implementing Monte‐Carlo torsional sampling, keeping only unique structures (RMSD>0.5 Å) and eliminating all structures with an energy 21 kJ mol^−1^ greater than the lowest energy structure. All resulting structures were then energy minimized by using conjugate gradient minimization, converging on a threshold of 0.05 kJ mol^−1^ Å^−1^. For each ligand, the ten lowest energy conformations were used to initiate docking. The docking consisted of further conformer sampling (generation of ring conformations switched off, to avoid potential generation of distorted galactopyranose ring conformations out of the well‐known low energy range around the ^4^C_1_ conformation), docking, and then energy minimization. Conformers were generated with 4× enhanced sampling and, during docking, the non‐bonded term of the potential energy function was softened for nonpolar ligand atoms (charge < |0.15|) by applying a scaling factor of 0.8. Finally, energy minimization was performed by using implicit solvent with a distance dependent dielectric constant of 4.

### Molecular dynamics: derivation of charges

The charges of all protein and carbohydrate atoms were obtained from AMBER ff14SB and GLYCAM 06j libraries, respectively. The charges of organics moieties were derived by using the RESP fitting method implemented on the RED server,^37^ following a similar protocol to previous publications.^38^, ^39^, ^40^ The non‐carbohydrate regions of both ligands **2** and **3** were each split into two fragments to increase computational efficiency, by disconnecting the bond between the sulfur and the anomeric carbon. In place of this bond, both ends were capped with a methyl group. Charges of individual fragments were computed by using the HF/6‐31G* level of theory with a weight factor of 0.01. All aliphatic protons were constrained to a charge of 0. During the fitting, the combined charge of the starred methyl and hydroxyl groups was set to zero, removing these groups in the final fitting and merging the fragments to give the full‐length non‐carbohydrate moiety. For ligand **2**, the charge of the sulfide methyl group was set to 0.194 throughout the fitting stages before removing to give a final fragment with net charge −0.194, in keeping with the modularity of the GLYCAM forcefield used for galactose charges.

### Molecular dynamics: initial coordinates

Initial coordinates for simulations of free CTB and the GM1/CTB complex were obtained from the Protein Data Bank (3CHB). Unresolved atoms were added to the coordinate files by using *tleap*, according to known protein chemistry and protonation state at physiological pH. Coordinates for simulations involving ligands **2** and **3** were generated by molecular docking as described above.

### Molecular dynamics: simulation protocol

All systems were parameterized by using AMBER ff14SB, GLYCAM_06j, and GAFF forcefields for the protein, carbohydrate, and organic moieties, respectively. Model systems were generated by solvating with explicit TIP4PEW water molecules within a truncated octahedral bounding box such that no solute atom was less than 10 Å from any face. An appropriate number of chloride ions were added to each system to neutralize the total charge. Conjugate gradient energy minimization was run with 100 kcal mol^−1^ Å^−2^ restraints on solute atoms, converging on a threshold of 1×10^−4^ kcal mol^−1^ Å^−1^, before repeating with no restraints. The system was then heated at constant volume to 300 K over a period of 100 ps by using a weak coupling algorithm with a time constant of 1 ps, employing periodic boundary conditions (PBC) with the particle mesh Ewald (PME) method. A constant temperature and pressure (1 atm) simulation was then run for 200 ps, using PBC with PME, to equilibrate the solvent density. A Langevin thermostat with a collision frequency of 2 ps^−1^ and a Berendsen barostat with a relaxation time of 2 ps were used. The SHAKE algorithm was used to restrain all bonds involving hydrogen, allowing a timestep of 2 fs to be used. A cutoff of 8 Å was used for non‐bonded interactions at all simulation stages. Using the same settings as for equilibration, classical molecular dynamics simulations were run for 100 ns, saving trajectory coordinates every 5 ps. For HREMD simulations, systems were prepared as above, differing only in the production dynamics stage. Eight replicas were generated from the same initial coordinates following equilibration and were each run for 12.5 ns. The non‐bonded potential between solvent atoms and non‐polar protein atoms (defined as carbon and sulfur) were scaled by a factor between 1 and 1.35 at intervals of 0.05 and exchanges between replicas was allowed every 2 ps. All trajectories were analyzed with *cpptraj*. All molecular graphics were generated with Schrodinger Maestro 11 version 2016‐4.

### CORCEMA‐ST predictions^28^, ^41^

Based on the experimental conditions, the concentration of ligand was 1 mm and the [P]/[L] ratio was kept fixed at 25:1. The cut distance around the binding pocket was 13 Å. *k*_on_ was set to 1×10^−8^ m ^−1^ and the irradiation frequency to the range −0.8 to 0.8 ppm. The bound ligand correlation time (*τ*) was 65 ns for both ligands, whereas the free correlation time was 1.2 ns, 1 ns, and 1 ns, respectively for ligands **2**, **1**, and **3**. The equilibrium constant used for ligand **2** was 2000 m ^−1^, 1000 m ^−1^ for ligand **1**, and 1500 m ^−1^ for ligand **3**, with *ρ* leak of 0.25. For ligands **2** and **3**, CORCEMA‐ST calculations were run in parallel on the High‐Performance Computing Unit, and then averaged to provide a representative build‐up curve for each ligand. The NOE *R*‐factor was calculated on the averaged simulated data obtained accordingly.

## Conflict of interest

The authors declare no conflict of interest.

## Biographical Information

*Jesús Angulo obtained his MSc and PhD in Chemistry from the University of Seville (US). He was a postdoc in the group of Prof. Thomas Peters at the University of Lübeck. He then was a “Juan de la Cierva” and “Ramón y Cajal” fellow at the CSIC in Seville (IIQ, 2006–2013). In 2013, he joined the School of Pharmacy at the University of East Anglia, becoming Associate Professor. Since 2020, he has been a Senior Distinguished Researcher at the US, leading the Biomolecular Interactions & Structural Glycobiology group, focusing on developing advanced NMR ligand‐observed techniques for protein–ligand interactions*.

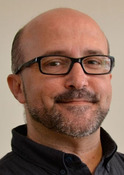

## Supporting information

As a service to our authors and readers, this journal provides supporting information supplied by the authors. Such materials are peer reviewed and may be re‐organized for online delivery, but are not copy‐edited or typeset. Technical support issues arising from supporting information (other than missing files) should be addressed to the authors.

Supplementary
