# Supplementary material for: Exploring Multi‐Subsite Binding Pockets in Proteins: DEEP‐STD NMR Fingerprinting and Molecular Dynamics Unveil a Cryptic Subsite at the GM1 Binding Pocket of Cholera Toxin B
Source: Chemistry. 2020 Jul 20;26(44):10024–34. doi: 10.1002/chem.202001723 (PMC7496166; doi:10.1002/chem.202001723)
Supplement: Supplementary file 1 — Supplementary [file CHEM-26-10024-s001.pdf]

Chemistry–A European Journal

Supporting Information

Exploring Multi-Subsite Binding Pockets in Proteins: DEEP-STD NMR Fingerprinting and Molecular Dynamics Unveil a Cryptic Subsite at the GM1 Binding Pocket of Cholera Toxin B**

Serena Monaco,[a] Samuel Walpole,[a] Hassan Doukani,[a] Ridvan Nepravishta,[a, b] Macarena Martínez-Bailén,[c] Ana T. Carmona,[c] Javier Ramos-Soriano,[c] Maria Bergström,[d] Inmaculada Robina,*[c] and Jesus Angulo*[a, c, e]

Author Contributions

S.M. Formal analysis: Lead; Investigation: Lead; Methodology: Equal; Validation: Lead; Visualization: Lead; Writing - Original Draft: Lead; Writing - Review & Editing: Equal
S.W. Formal analysis: Equal; Investigation: Equal; Methodology: Equal; Software: Equal; Validation: Equal; Visualization: Supporting; Writing - Original Draft: Supporting; Writing - Review & Editing: Supporting
H.D. Formal analysis: Equal; Investigation: Equal; Methodology: Supporting; Validation: Supporting; Visualization: Equal
R.N. Formal analysis: Supporting; Investigation: Supporting; Methodology: Equal; Supervision: Equal; Validation: Supporting; Visualization: Equal; Writing - Original Draft: Equal; Writing - Review & Editing: Equal
M.M. Investigation: Equal; Methodology: Equal; Validation: Equal; Visualization: Equal; Writing - Original Draft: Supporting; Writing - Review & Editing: Supporting
A.C. Formal analysis: Equal; Investigation: Equal; Supervision: Equal; Validation: Equal; Writing - Original Draft: Equal; Writing - Review & Editing: Equal
J.R. Formal analysis: Supporting; Investigation: Supporting; Validation: Equal; Visualization: Equal; Writing - Original Draft: Supporting; Writing - Review & Editing: Supporting
M.B. Methodology: Supporting; Validation: Supporting; Visualization: Supporting; Writing - Review & Editing: Equal
I.R. Formal analysis: Equal; Investigation: Equal; Methodology: Equal; Supervision: Lead; Validation: Equal; Visualization: Equal; Writing - Original Draft: Supporting; Writing - Review & Editing: Equal
J.A. Conceptualization: Lead; Formal analysis: Supporting; Funding acquisition: Lead; Investigation: Lead; Methodology: Lead; Project administration: Lead; Resources: Lead; Supervision: Lead; Validation: Equal; Writing - Original Draft: Equal; Writing - Review & Editing: Lead.

- Experimental procedures for the synthesis of intermediates 11b, 12b and 15-18 [Pag. 2-6]
- ^{1}H and ^{13}C NMR spectra of new compounds [Pag. 7-19]
- Binding epitopes of ligands 1, 2, and 3[1] [Pag. 20]
- ^{1}H assignment of 4-9, raw build-up curves data and normalized STD values [Pag. 20-23]
- DEEP-STD NMR experiments for ligands 4-7 and 3NPG in complex with CTB [Pag. 24]
- Competition experiments: STD NMR spectra [Pag. 25-26]
- STD NMR experiment on the 3'SL/CTB binary complex [Pag. 26]
- Control tr-NOESY spectra [Pag. 27-28]
- Rigid molecular docking of ligands 2 and 3. [Pag. 29]
- Ligand 1 docking solution and CORCEMA-ST validation [Pag. 30]
- Ligands 2 and 3 docking solutions [Pag. 31-32]
- HREMD Simulations of the CTB pentamer [Pag. 32]
- HREMD Simulations of the CTB pentamer bound GM1 [Pag. 33]
- MD Simulations of the CTB pentamer bound to ligand 3 [Pag. 34-35]
- CORCEMA-ST validation ligands 3 and 2 (from MD frames) [Pag. 36]
- References [Pag. 37]

Experimental procedures for the synthesis of intermediates 11b, 12b and 15-18.

General methods

Optical rotations were measured in a Jasco P-2000 spectropolarimeter in a 1.0 cm or 1.0 dm tube (Na, λ 598 nm). Infrared spectra were recorded with a Jasco FTIR-410 spectrophotometer. ^{1}H- and ^{13}C-NMR spectra were recorded with a Bruker AMX300 spectrometer for solutions in $CDCl_3$ or CD_3OD. δ are given in ppm and *J* in Hz. *J* are assigned and not repeated. All the assignments were confirmed by 2D spectra (COSY and HSCQ). High resolution mass spectra were recorded on a Q-Exactive spectrometer. TLC was performed on silica gel 60 F_{254} (Merck), with detection by UV light charring with H_2SO_4 or with reagent [$(NH_4)_6MoO_4$, $Ce(SO_4)_2$, H_2SO_4, H_2O]. Silica gel 60 (Merck, 40-60 and 63-200 μm) was used for preparative chromatography.

Benzyl 5-(chloromethyl)-2-methylfuran-3-carboxylate (11b)

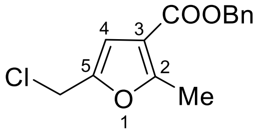

11b

Compound **10**[1] (8.14 g, 24.2 mmol) was dissolved in MeOH (65 mL) and the solution was cooled to 0 ºC. Then a solution of $NaIO_4$ (11.9 g, 55.7 mmol) in water (90 mL) was slowly added and the mixture stirred at r.t. for 2 h. Evaporation of the solvent and purification through column chromatography on silica gel (EtOAc:cyclohexane 1:10→1:5) afforded the corresponding aldehyde (3.08 g, 12.6 mmol, 52%) that was subsequently treated with $NaBH_4$ (954 mg, 25.2 mmol) in MeOH (30 mL) for 1 h at r.t. Then, sat. aq. soln. of citric acid was added and the mixture was diluted with CH_2Cl_2 and washed with water and brine. The organic phase was dried over Na_2SO_4, filtered and evaporated. Chromatography purification on silica gel (EtOAc: cyclohexane 1:3→1:2) afforded **11a** (2.65 g, 10.8 mmol, 86%) as a yellow solid. IR (ῡ) 3395 (OH), 2941, 1712 (C=O), 1214, 1072 cm^{-1}. ^{1}H NMR (300 MHz, CD_3OD) δ 7.42-7.27 (m, 5H, H-Ar), 6.52 (s, 1H, H-4), 5.24 (s, 2H, C*H*$_2$Ph), 4.44 (s, 2H, C*H*$_2$OH), 2.52 (s, 3H, Me). ^{13}C NMR (75.4 MHz, CD_3OD) δ 165.2 (*C*OOBn), 160.6, 154.4 (C-2, C-5), 137.7 (C_q-Ar), 129.6, 129.2, 129.1 (C-Ar), 114.9 (C-3), 109.0 (C-4), 67.0 (*C*H$_2$Ph), 57.0 (*C*H$_2$OH), 13.8 (Me). HRCIMS *m/z* found 246.0891, calcd. for $C_{14}H_{14}O_4$ $(M)^+$: 246.0892. To a solution of *N*-chlorosuccinimide (584 mg, 4.29 mmol) in anhydrous CH_2Cl_2 (6 mL), Me_2S (320 μL, 4.29 mmol) was added. A solution of **11a** (705 mg, 2.86 mmol) in anhydrous CH_2Cl_2 (8 mL) previously cooled to -20 ºC was subsequently added and the reaction mixture was stirred at 0 ºC for 1 hour. The solvent was evaporated and the resulting **11b** was directly used for the next reaction without further purification.

Benzyl-5-(thiomethyl)-2-methylfuran-3-carboxylate (12b)

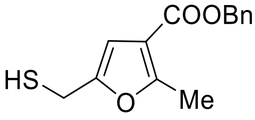

12b

To a solution of **11b** (757 mg, 2.86 mmol) in anhydrous DMF (16 mL) and KSAc (653 mg, 5.72 mmol) was added. After stirring at 50 °C for 2 h, the solvent was removed under reduced pressure and the obtained residue was dissolved in CH_2Cl_2, washed with water and brine, dried over Na_2SO_4, filtered and evaporated. The crude product was purified by column chromatography on silica gel (EtOAc:cyclohexane 1:20) to give **12a** (763 mg, 2.51 mmol, 88%) as a yellow oil. IR (ῡ) 1695 (C=O), 1211, 1067 cm^{-1}. ^1H NMR (300 MHz, $CDCl_3$) δ 7.42-7.29 (m, 5H, H-Ar), 6.49 (s, 1H, H-4), 5.25 (s, 2H, CH_2Ph), 4.06 (s, 2H, CH_2S), 2.54 (s, 3H, Me), 2.35 (s, 3H, CH_3CO). ^{13}C NMR (75.4 MHz, $CDCl_3$) δ 194.3 (CH_3*C*O), 163.7 (*C*OOBn), 159.4, 148.6 (C-2, C-5), 136.3 (C_q-Ar), 128.7, 128.3, 128.2 (C-Ar), 114.2 (C-3), 108.8 (C-4), 66.0 (*C*H_2Ph), 30.4 (*C*H_2S), 25.7 (*C*H_3CO), 13.9 (Me). HRESIMS *m/z* found 327.0650, calcd. for $C_{16}H_{16}O_4NaS$ $(M+Na)^+$: 327.0662. To a solution of **12a** (690 mg, 2.27 mmol) in anhydrous MeOH (25 mL), NaOMe (4.5 mL, 0.5 M in MeOH) was added and the mixture stirred at 0 °C for 1 h. Then the mixture was neutralized with Amberlite IR-120H^+, filtered, and washed with MeOH. Evaporation of the solvent gave a residue that was purified by column chromatography on silica gel (CH_2Cl_2:cyclohexane 2:3) to give **12b** (562 mg, 2.14 mmol, 94%) as a yellow liquid. IR (ῡ) 1707 (C=O), 1208, 1067, 693 cm^{-1}. ^1H NMR (300 MHz, $CDCl_3$) δ 7.43-7.30 (m, 5H, H-Ar), 6.45 (s, 1H, H-4), 5.27 (s, 2H, CH_2Ph), 3.66 (d, 2H, $J_{H,H}$ = 7.8, CH_2SH), 2.56 (s, 3H, Me), 1.89 (t, 1H, $J_{H,H}$ = 7.8, S*H*). ^{13}C NMR (75.4 MHz, $CDCl_3$) δ 163.8 (*C*OOBn), 159.2, 151.7 (C-2, C-5), 136.3 (C_q-Ar), 128.7, 128.3, 128.2 (C-Ar), 114.1 (C-3), 107.3 (C-4), 66.0 (*C*H_2Ph), 20.9 (*C*H_2SH), 14.0 (Me). HRESIMS *m/z* found 285.0546, calcd. for $C_{14}H_{14}O_3NaS$ $(M+Na)^+$: 285.0556.

((4-Benzyloxycarbonyl-5-methylfuran-2-yl)methyl)-2,3,4,6-tetra-*O*-acetyl-1-thio-α-D-galactopyranoside (15)

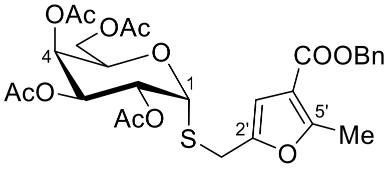

15

A solution of **13**[2] (282 mg, 0.694 mmol) in anhydrous DMF (6 mL) was added **11b** (276 mg, 1.04 mmol). Then, Et_2NH (590 µL, 5.68 mmol) was added and the reaction mixture was stirred at r.t. for 1 hour. After this time, the solvent was removed under vacuo and the resulting crude was purified by column

chromatography on silica gel (Toluene:acetone 18:1→15:1→12:1) to give **15** (198 mg, 0.334 mmol, 48%) as a colourless oil. $[\alpha]_D^{25}$ = 194.9 (*c* 0.86, CH_2Cl_2). IR (ū) 2945, 1743 (C=O), 1713 (C=O), 1213, 1067 cm^{-1}. ^1H NMR (300 MHz, $CDCl_3$) δ 7.43-7.32 (m, 5H, H-Ar), 6.45 (s, 1H, H-3'), 5.70 (d, 1H, $J_{1,2}$ = 5.6, H-1), 5.46 (dd, 1H, $J_{4,3}$ = 3.2, $J_{4,5}$ = 1.1, H-4), 5.33-5.26 (m, 3H, H-2, CH_2Ph), 5.20 (dd, 1H, $J_{3,2}$ = 10.8, H-3), 4.55 (ap. t, 1H, H-5), 4.17-4.05 (m, 2H, H-6), 3.73 (d, 1H, $^2J_{H,H}$ = 14.8, CH_2S), 3.60 (d, 1H, CH_2S), 2.55 (s, 3H, Me), 2.14 (s, 3H, CH_3CO), 2.03 (s, 3H, CH_3CO), 2.02 (s, 3H, CH_3CO), 1.98 (s, 3H, CH_3CO). ^{13}C NMR (75.4 MHz, $CDCl_3$) δ 170.4, 170.3, 170.05, 170.0 (CH_3*C*O), 163.7 (*C*OOBn), 159.6, 148.4 (C-2', C-5'), 136.3 (C_q-Ar), 128.7, 128.31, 128.29 (C-Ar), 114.2 (C-4'), 109.0 (C-3'), 81.4 (C-1), 68.4 (C-3), 67.9 (C-4), 67.7 (C-2), 67.0 (C-5), 66.1 (*C*H_2Ph), 61.8 (C-6), 25.2 (*C*H_2S), 20.9, 20.81, 20.75 (*C*H_3CO), 14.0 (Me). HRESIMS *m/z* found 615.1492, calcd. for $C_{28}H_{32}O_{12}NaS$ $(M+Na)^+$: 615.1507.

((4-Benzyloxycarbonyl-5-methylfuran-2-yl)methyl)-2,3,4,6-tetra-*O*-acetyl-1-thio-β-D-galactopyranoside (16)

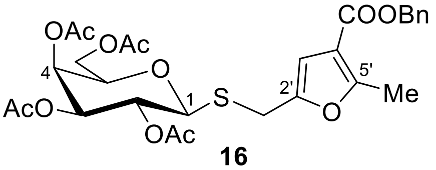

16

A solution of **14** (617 mg, 1.50 mmol) in EtOAc (6 mL) was added to a solution of **12b** (220 mg, 0.83 mmol) in a 10% aqueous solution of Na_2CO_3 (7 mL). Then, TBAHS (1.17 g, 3.33 mmol) was added and the reaction mixture was stirred vigorously at r.t. overnight. Then, the reaction mixture was diluted with EtOAc (20 mL) and washed with a saturated aqueous solution of $NaHCO_3$ and brine. The organic layer was dried over Na_2SO_4, filtered, and evaporated. The resulting crude was purified by column chromatography on silica gel (Et_2O:cyclohexane, 1:1) to give **16** (353 mg, 0.60 mmol, 72%) as a yellow oil. $[\alpha]_D^{25}$ = -40.9 (*c* 0.83, CH_2Cl_2). IR (ū) 1743 (C=O), 1710 (C=O), 1364, 1211, 1047 cm^{-1}. ^1H NMR (300 MHz, $CDCl_3$) δ 7.42-7.30 (m, 5H, H-Ar), 6.49 (s, 1H, H-3'), 5.41 (dd, 1H, $J_{4,3}$ = 3.4, $J_{4,5}$ = 1.0, H-4), 5.29-5.21 (m, 3H, CH_2Ph, H-2), 5.02 (dd, 1H, $J_{3,2}$ = 10.0, H-3), 4.45 (d, 1H, $J_{1,2}$ = 10.0, H-1), 4.13 (dd, 1H, $^2J_{6a,6b}$ = 11.3, $J_{6a,5}$ = 6.8, H-6a), 4.08 (dd, 1H, $J_{6b,5}$ = 6.3, H-6b), 3.95 (d, 1H, $^2J_{H,H}$ = 14.7, CH_2S), 3.88 (td, 1H, H-5), 3.74 (d, 1H, CH_2S), 2.57 (s, 3H, Me), 2.14 (s, 3H, CH_3CO), 2.02 (s, 6H, CH_3CO), 1.97 (s, 3H, CH_3CO). ^{13}C NMR (75.4 MHz, $CDCl_3$) δ 170.5, 170.4, 170.2, 169.7 (CH_3*C*O), 163.7 (*C*OOBn), 159.6, 148.5 (C-2', C-5'), 136.2 (C_q-Ar), 128.7, 128.4, 128.3 (C-Ar), 114.2 (C-4'), 109.1 (C-3'), 82.8 (C-1), 74.7 (C-5), 71.9 (C-3), 67.4 (C-4), 67.2 (C-2), 66.2 (*C*H_2Ph), 61.6 (C-6), 26.0 (*C*H_2S), 20.83, 20.75, 20.74, 20.69 (*C*H_3CO), 14.1 (Me). HRESIMS *m/z* found 615.1499, calcd. for $C_{28}H_{32}O_{12}NaS$ $(M+Na)^+$: 615.1507.

((4-Benzyloxycarbonyl-5-methylfuran-2-yl)methyl)-2,3,4,6-tetra-*O*-acetyl-1-sulfonyl-α-D-galactopyranoside (17)

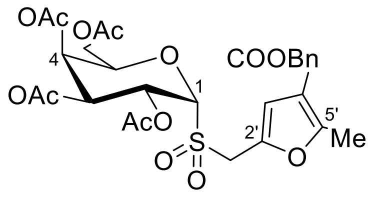

17

To a 0 ºC solution of **15** (110 mg, 0.186 mmol) in CH_2Cl_2 (5 mL), MCPBA (115 mg, 0.465 mmol) was added and the mixture stirred at r.t. for 2.5 h. Then, the crude was washed with sat. aq. soln. of $NaHCO_3$, water and brine, dried over Na_2SO_4, filtered and evaporated. The resulting residue was purified by column chromatography on silica gel (EtOAc:cyclohexane 1:3→1:2) to give **17** (74 mg, 0.12 mmol, 65%) as a white solid. $[\alpha]_D^{25}$ = 108.0 (*c* 0.85, CH_2Cl_2). IR (ῡ) 1748 (C=O), 1715 (C=O), 1216, 1074 cm^{-1}. ^1H NMR (300 MHz, $CDCl_3$) δ 7.42-7.33 (m, 5H, H-Ar), 6.78 (s, 1H, H-3'), 5.82 (dd, 1H, $J_{3,2}$ = 10.8, $J_{3,4}$ = 3.3, H-3), 5.58 (d, 1H, H-4), 5.49 (dd, 1H, $J_{2,1}$ = 6.9, H-2), 5.34-5.23 (m, 3H, H-1, C*H₂*Ph), 4.88-4.84 (m, 1H, H-5), 4.58 (d, 1H, $^2J_{H,H}$ = 15.0, CH_2S), 4.23-4.15 (m, 2H, CH_2S, H-6a), 4.09 (dd, 1H, $^2J_{6b,6a}$ = 11.7, $J_{6b,5}$ = 4.7, H-6b), 2.59 (s, 3H, Me), 2.16 (s, 3H, CH_3CO), 2.10 (s, 3H, CH_3CO), 2.05 (s, 3H, CH_3CO), 2.01 (s, 3H, CH_3CO). ^{13}C NMR (75.4 MHz, $CDCl_3$) δ 170.9, 170.2, 170.0, 169.6 (CH_3CO), 163.2 (*C*OOBn), 161.3, 139.5 (C-2', C-5'), 136.0 (C_q-Ar), 128.8, 128.43, 128.36 (C-Ar), 115.2 (C-4'), 113.7 (C-3'), 84.2 (C-1), 71.6 (C-5), 67.21, 67.19 (C-3, C-4), 66.3 (CH_2Ph), 65.8 (C-2), 62.5 (C-6), 50.7 (CH_2S), 20.8, 20.74, 20.70, 20.66 (CH_3CO), 14.2 (Me). HRESIMS *m/z* found 647.1401, calcd. for $C_{28}H_{32}O_{14}NaS$ $(M+Na)^+$: 647.1405.

((4-Benzyloxycarbonyl-5-methylfuran-2-yl)methyl)-2,3,4,6-tetra-*O*-acetyl-1-sulfonyl-β-D-galactopyranoside (18)

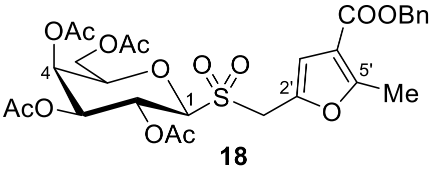

18

To a solution of **16** (140 mg, 0.236 mmol) in CH_2Cl_2 (6 mL), MCPBA (146 mg, 0.59 mmol) was added and the mixture stirred at r.t. for 3 h. Then, sat. aq. soln. of $NaHCO_3$ was added and the mixture was extracted with CH_2Cl_2. The combined organic phases were washed with sat. aq. soln. of $NaHCO_3$ and brine, dried over Na_2SO_4, filtered and evaporated. The resulting residue was purified by column chromatography on silica gel (EtOAc:cyclohexane 1:2) to give **18** (109 mg, 0.175 mmol, 74%) as a white solid. $[\alpha]_D^{25}$ = -18.3 (*c* 0.74, CH_2Cl_2). IR (ῡ) 1746 (C=O), 1713 (C=O), 1205, 1062 cm^{-1}. ^1H NMR (300

MHz, $CDCl_3$) δ 7.42-7.32 (m, 5H, H-Ar), 6.83 (s, 1H, H-3'), 5.73 (t, 1H, $J_{2,1}$ = $J_{2,3}$ = 9.9, H-2), 5.47 (dd, 1H, $J_{4,3}$ = 3.4, $J_{4,5}$ = 1.0, H-4), 5.27 (d, 2H, $^2J_{H,H}$ = 2.5, CH_2Ph), 5.11 (dd, 1H, H-3), 4.61 (d, 1H, $^2J_{H,H}$ = 15.0, CH_2S), 4.44 (d, 1H, H-1), 4.27-4.08 (m, 3H, CH_2S, H-6), 4.07-4.02 (m, 1H, H-5), 2.61 (s, 3H, Me), 2.18 (s, 3H, CH_3CO), 2.03 (s, 3H, CH_3CO), 2.02 (s, 3H, CH_3CO), 1.99 (s, 3H, CH_3CO). ^{13}C NMR (75.4 MHz, $CDCl_3$) δ 170.4, 170.2, 170.1, 169.2 (CH_3*C*O), 163.2 (*C*OOBn), 161.4, 139.5 (C-2', C-5'), 136.0 (C_q-Ar), 128.8, 128.5, 128.4 (C-Ar), 115.2 (C-4'), 113.8 (C-3'), 86.7 (C-1), 76.0 (C-5), 71.3 (C-3), 67.0 (C-4), 66.4 (*C*H_2Ph), 63.1 (C-2), 61.8 (C-6), 49.2 (*C*H_2S), 20.78, 20.74, 20.67, 20.62 (*C*H_3CO), 14.2 (Me). HRESIMS *m/z* found 647.1414, calcd. for $C_{28}H_{32}O_{14}NaS$ $(M+Na)^+$: 647.1405.

^{1}H and ^{13}C NMR spectra of new compounds

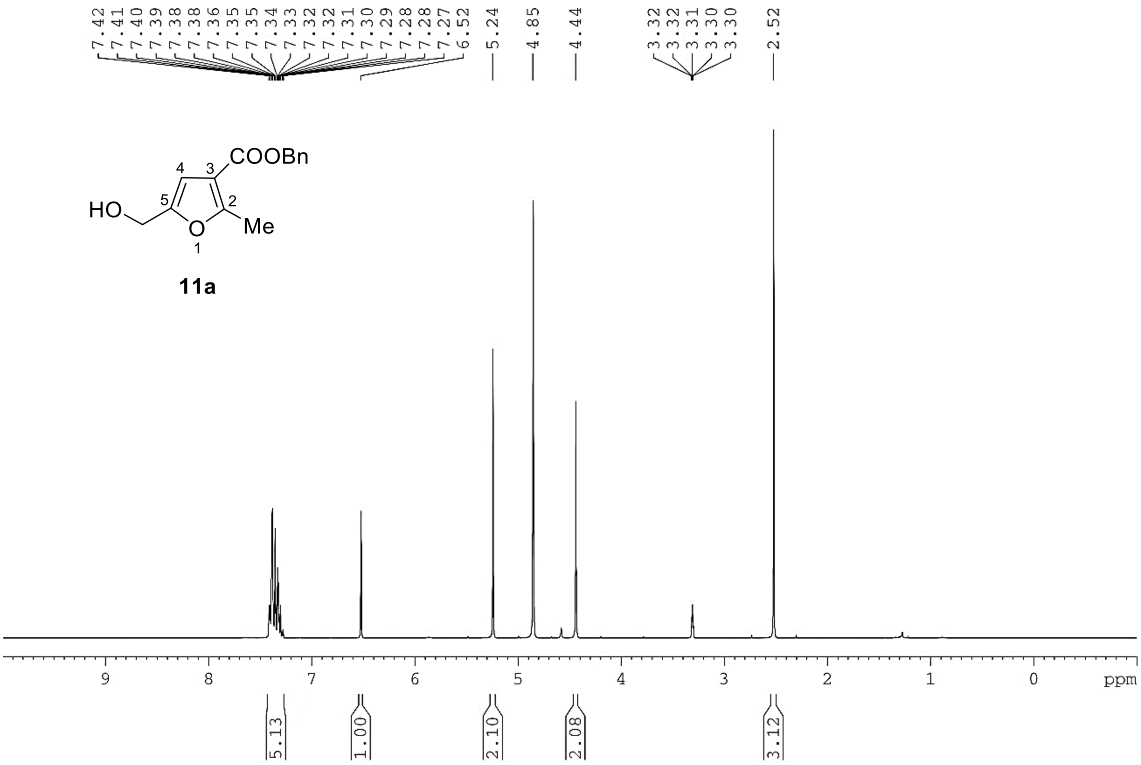

^{1}H NMR (300 MHz, CD_3OD)

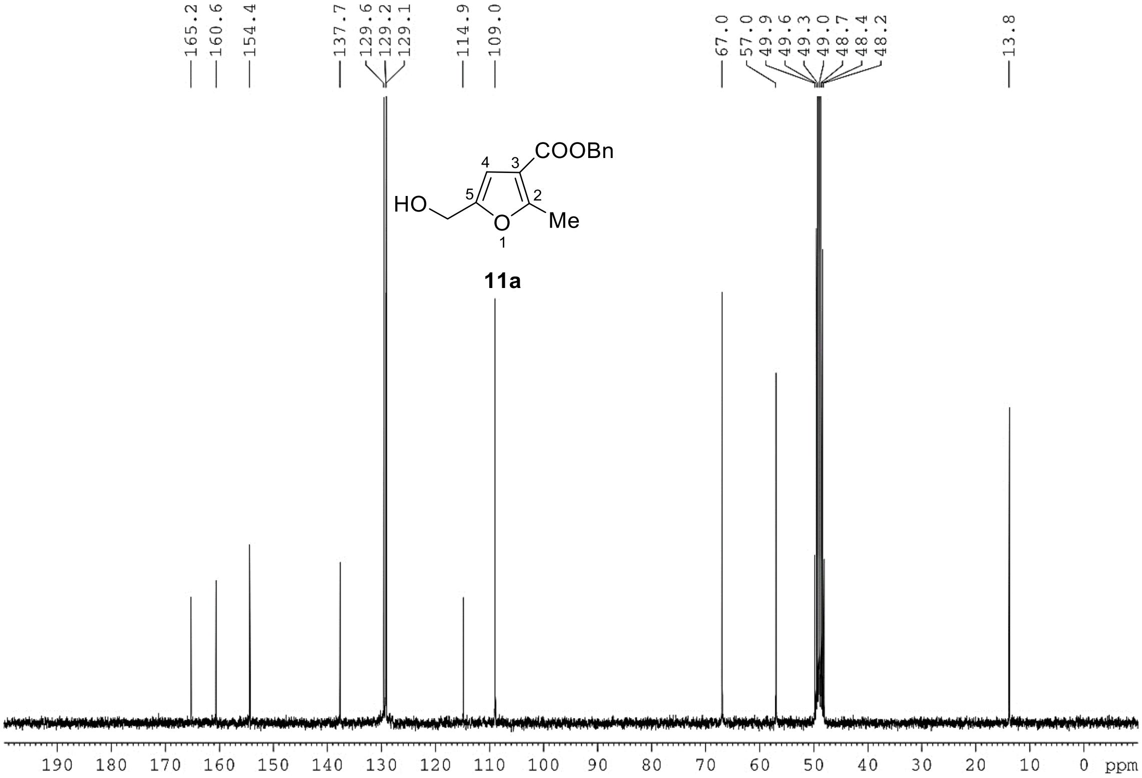

^{13}C NMR (75.4 MHz, CD_3OD)

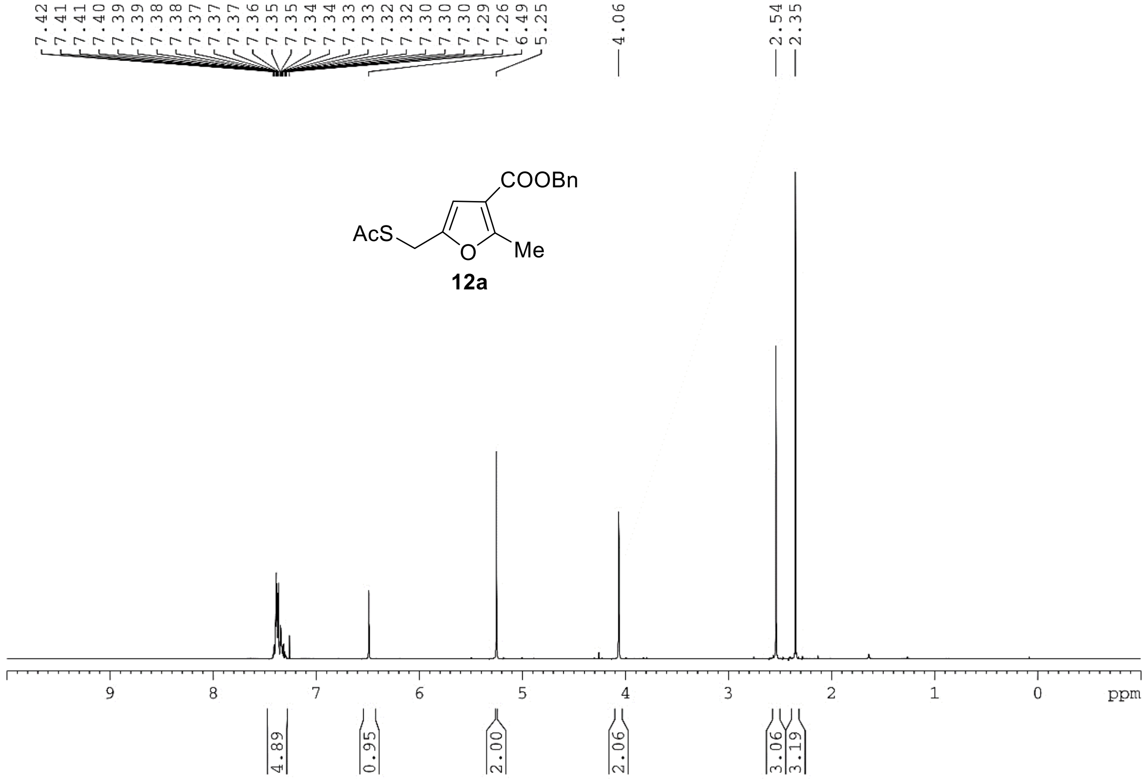

^{1}H NMR (300 MHz, CDCl$_3$)

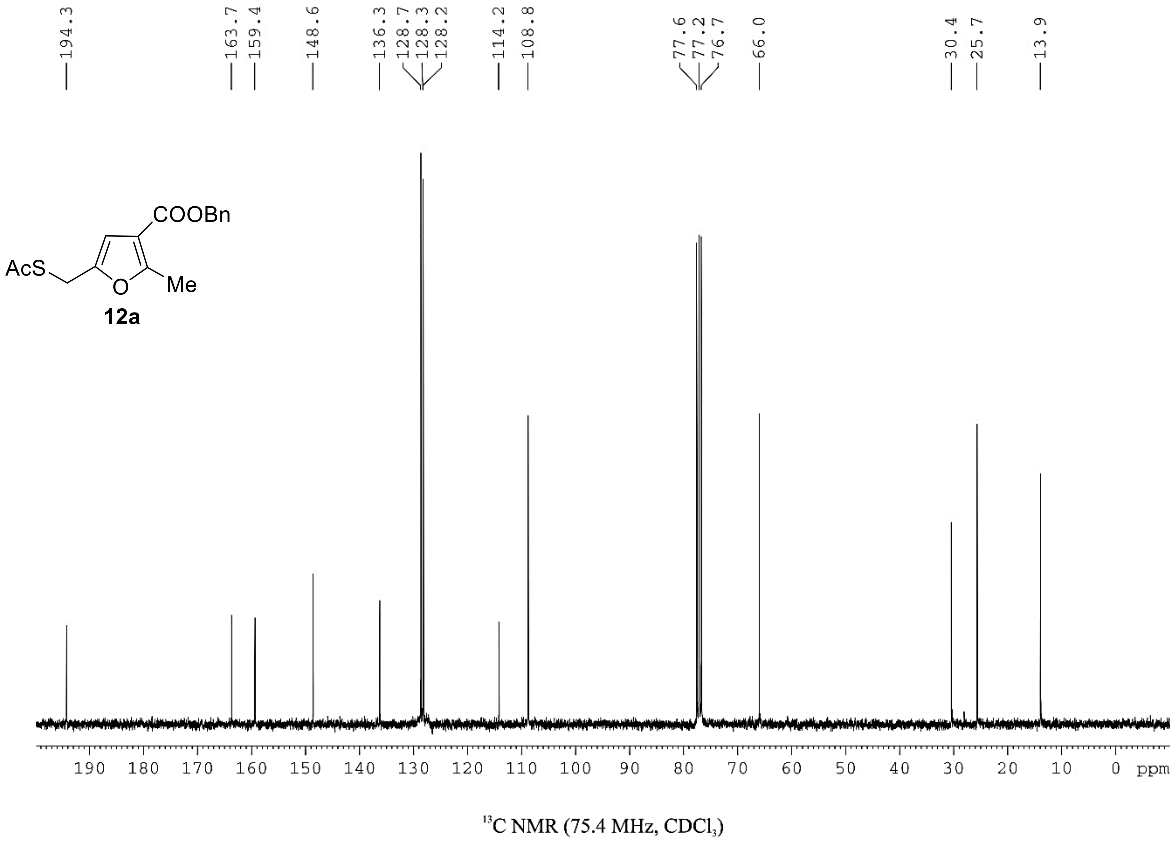

^{13}C NMR (75.4 MHz, CDCl$_3$)

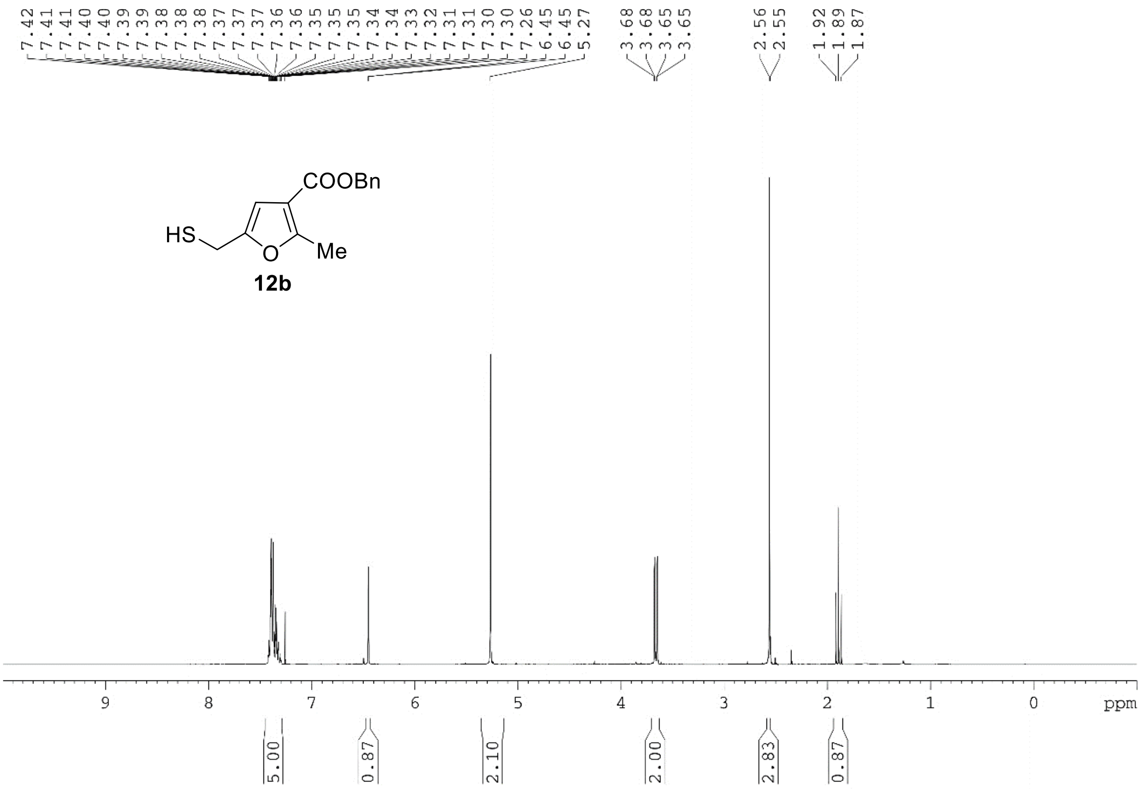

^{1}H NMR (300 MHz, $CDCl_3$)

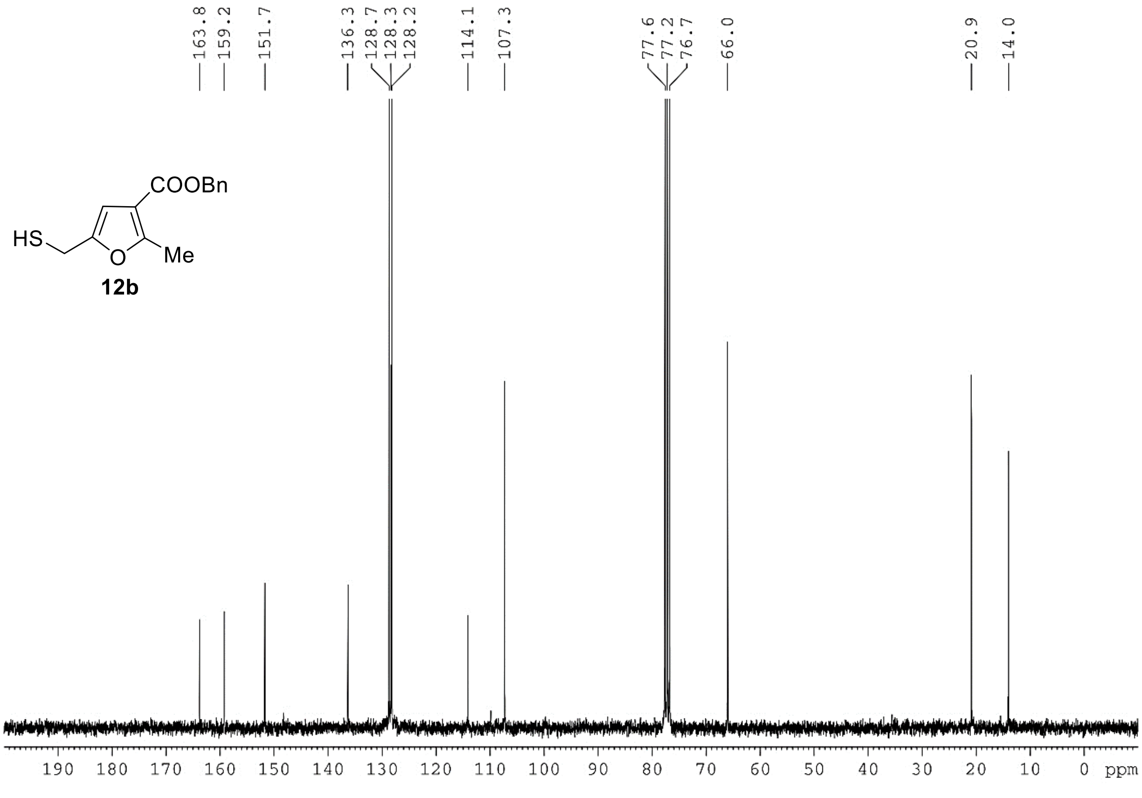

^{13}C NMR (75.4 MHz, $CDCl_3$)

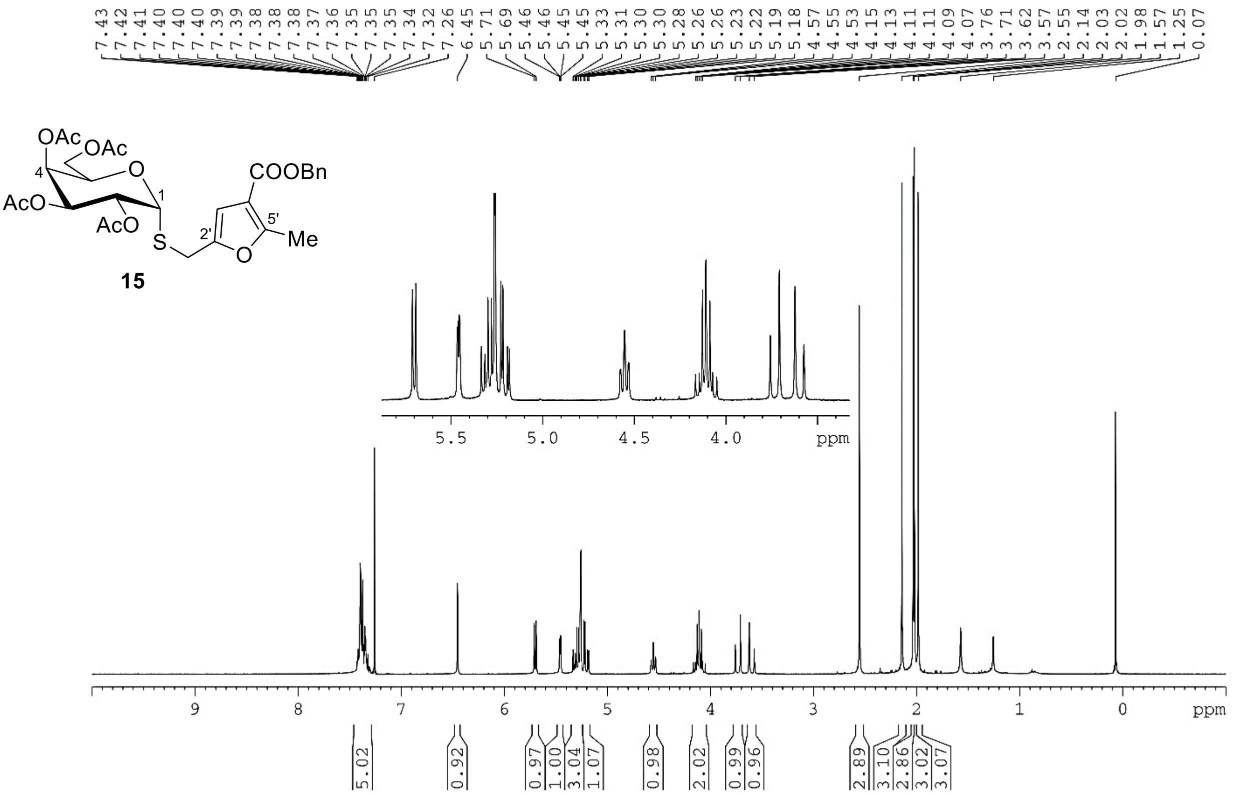

^{1}H NMR (300 MHz, $CDCl_3$)

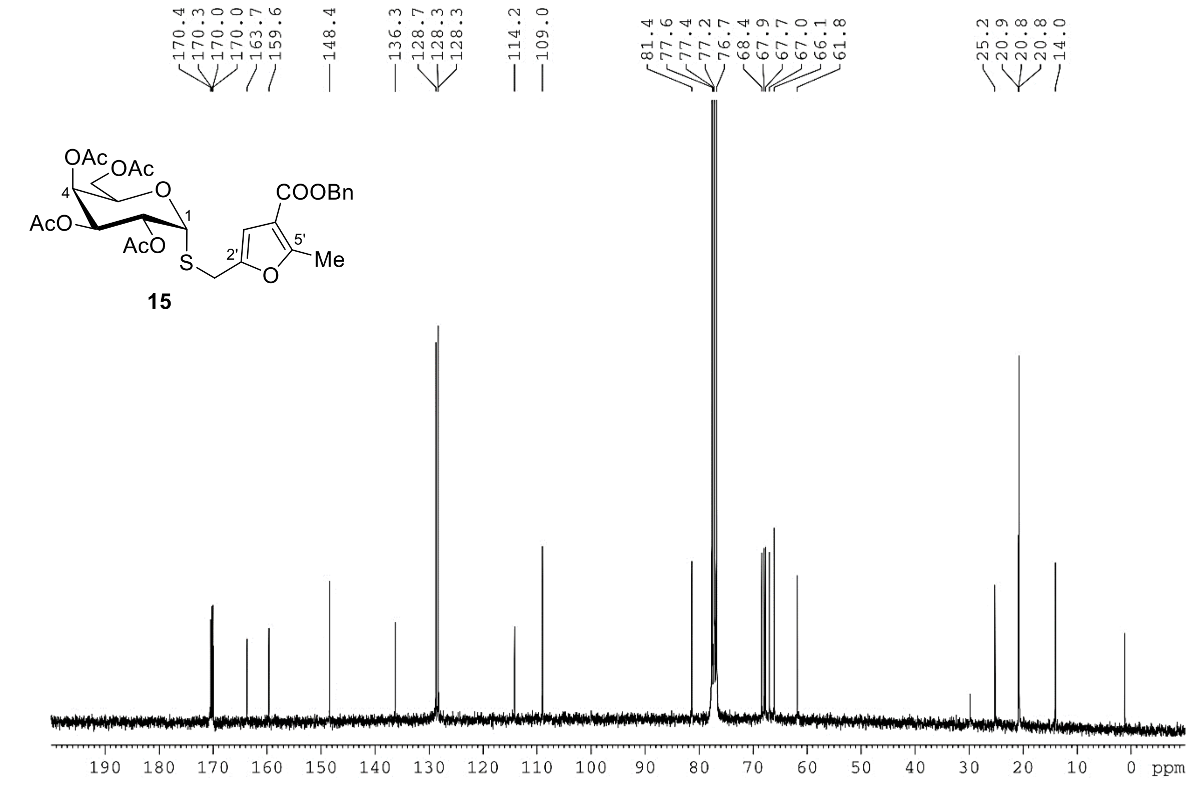

^{13}C NMR (75.4 MHz, $CDCl_3$)

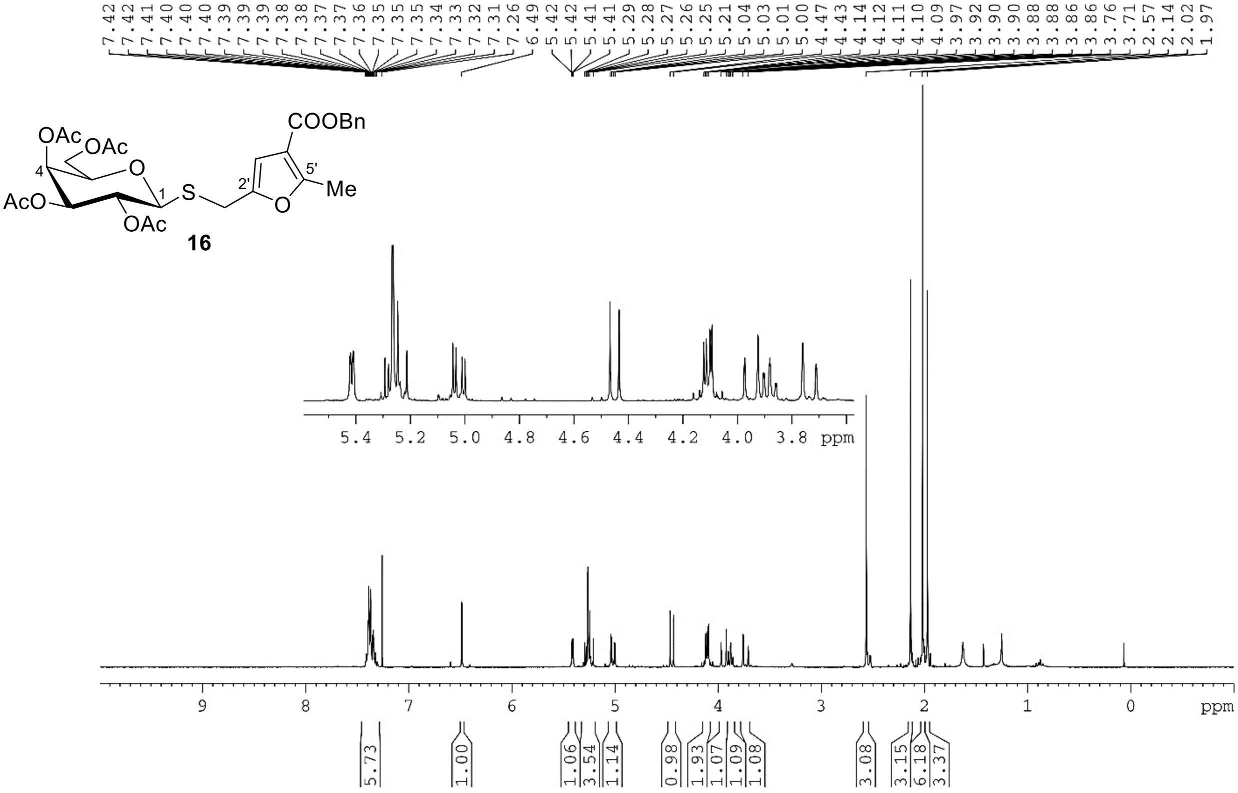

^{1}H NMR (300 MHz, $CDCl_3$)

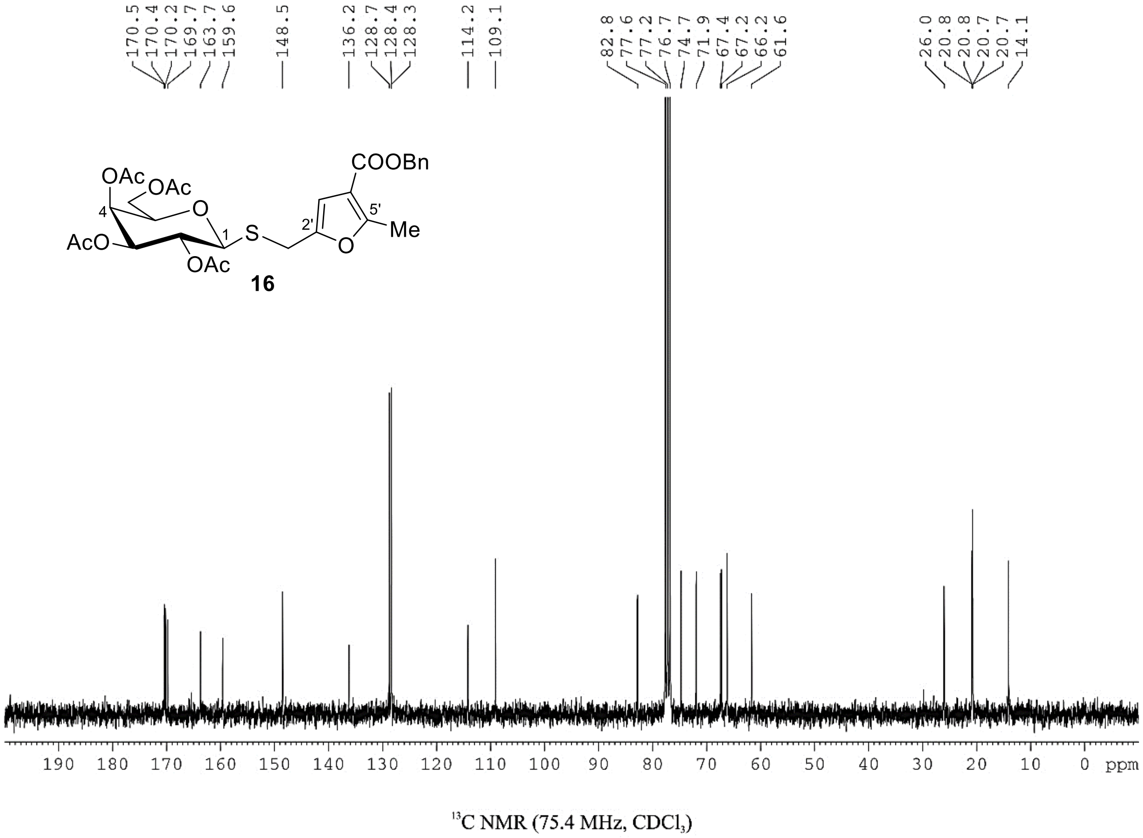

^{13}C NMR (75.4 MHz, $CDCl_3$)

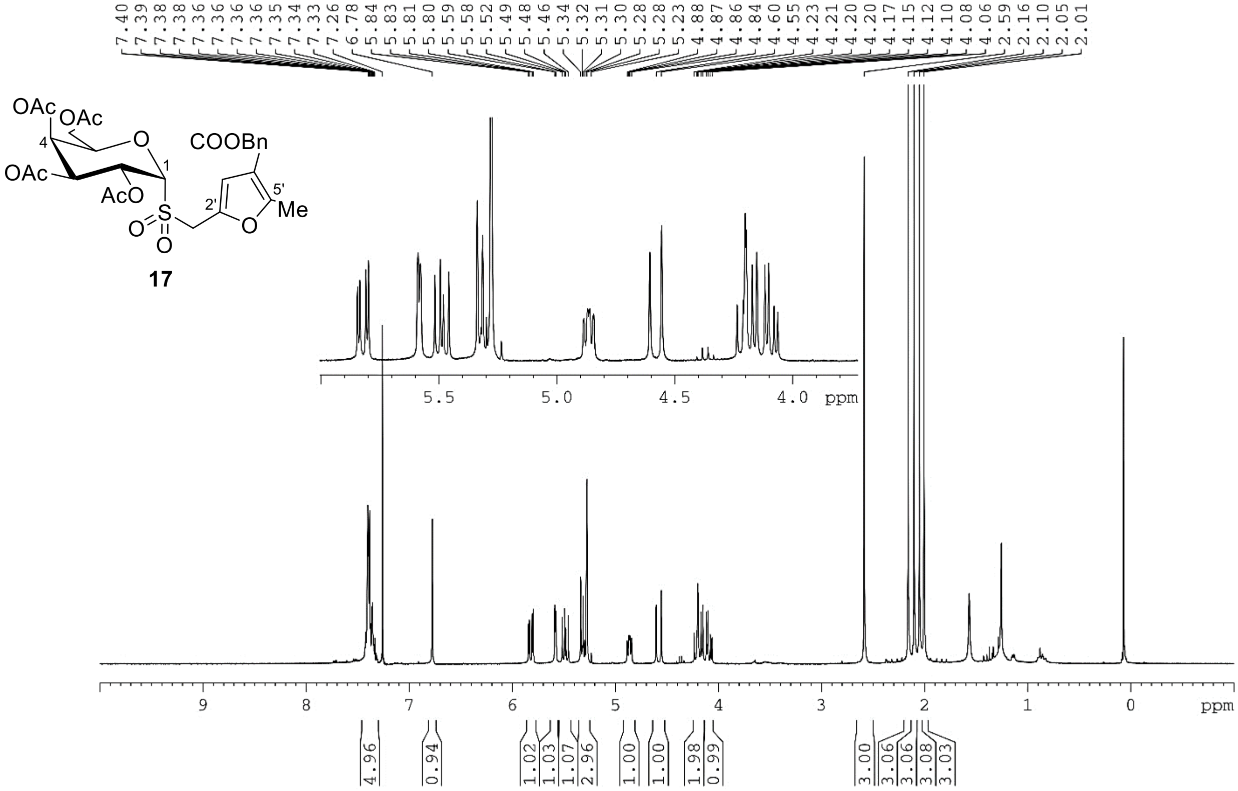

^{1}H NMR (300 MHz, $CDCl_3$)

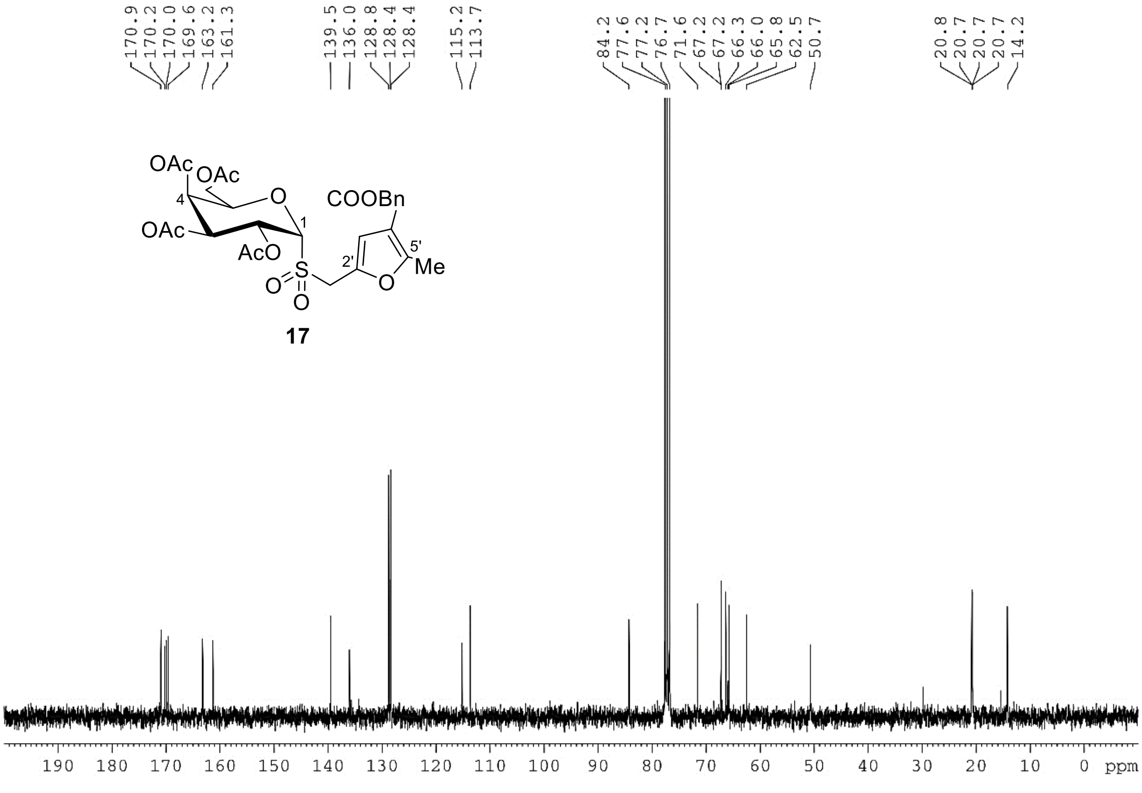

^{13}C NMR (75.4 MHz, $CDCl_3$)

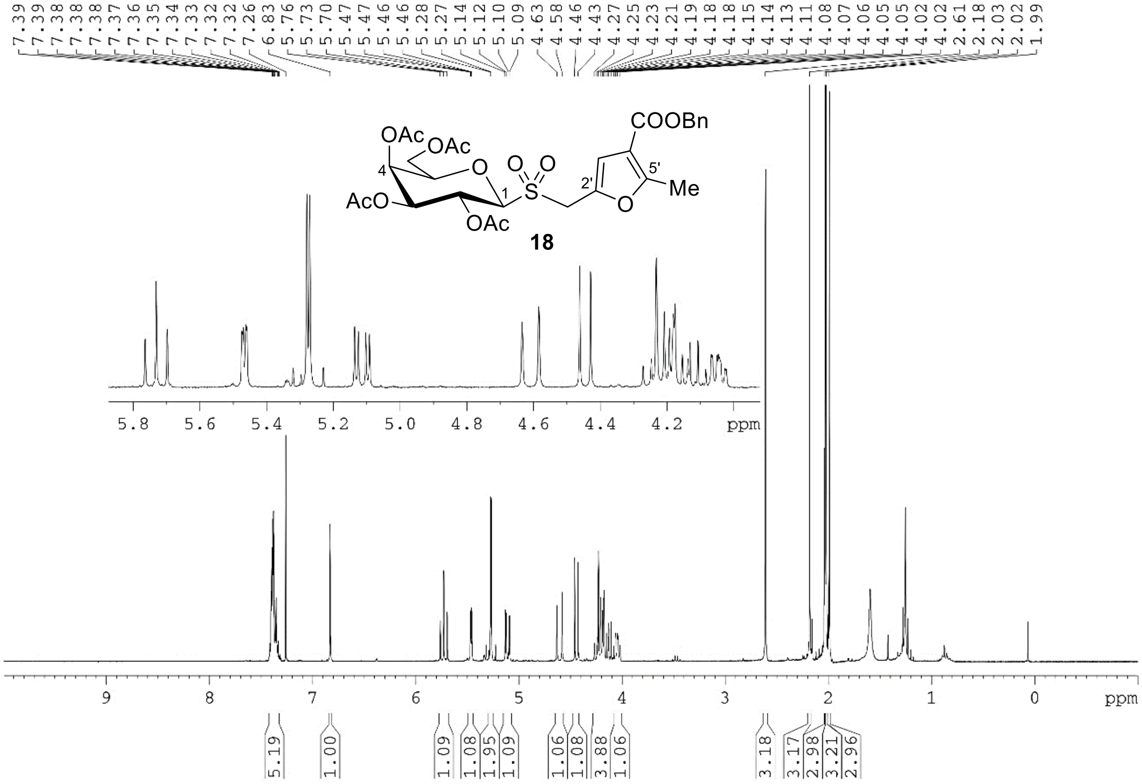

^{1}H NMR (300 MHz, $CDCl_3$)

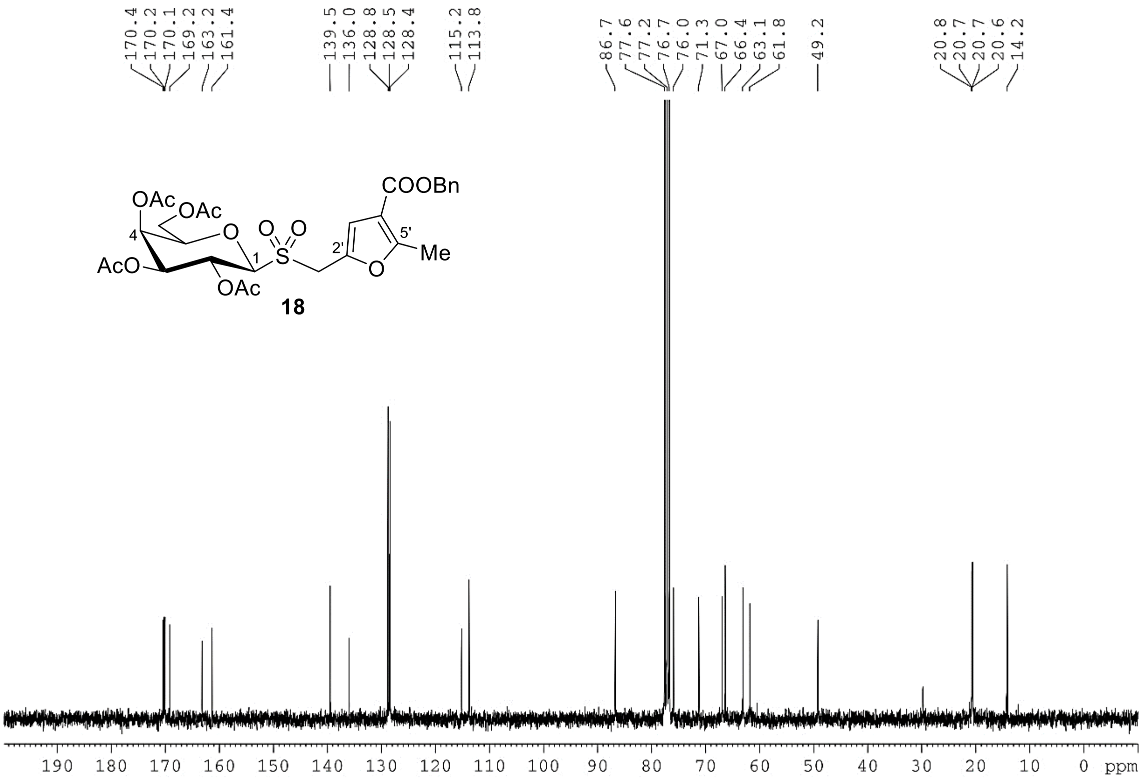

^{13}C NMR (75.4 MHz, $CDCl_3$)

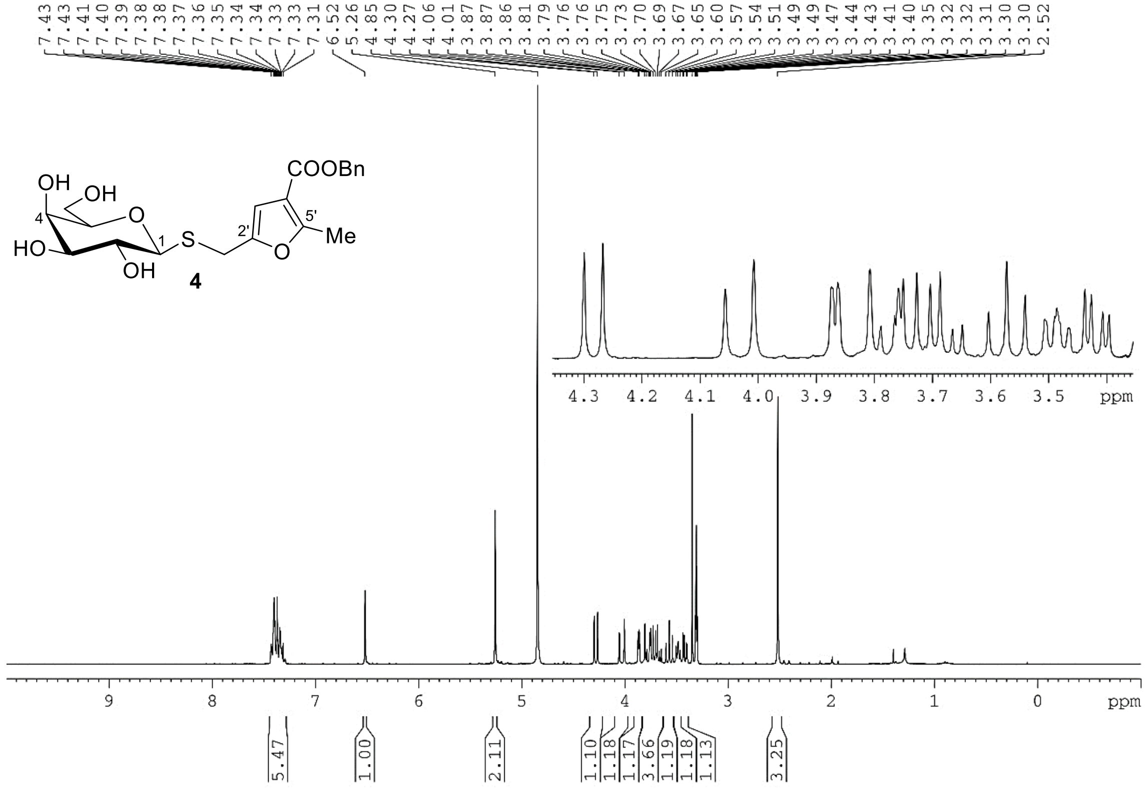

^{1}H NMR (300 MHz, CD_3OD)

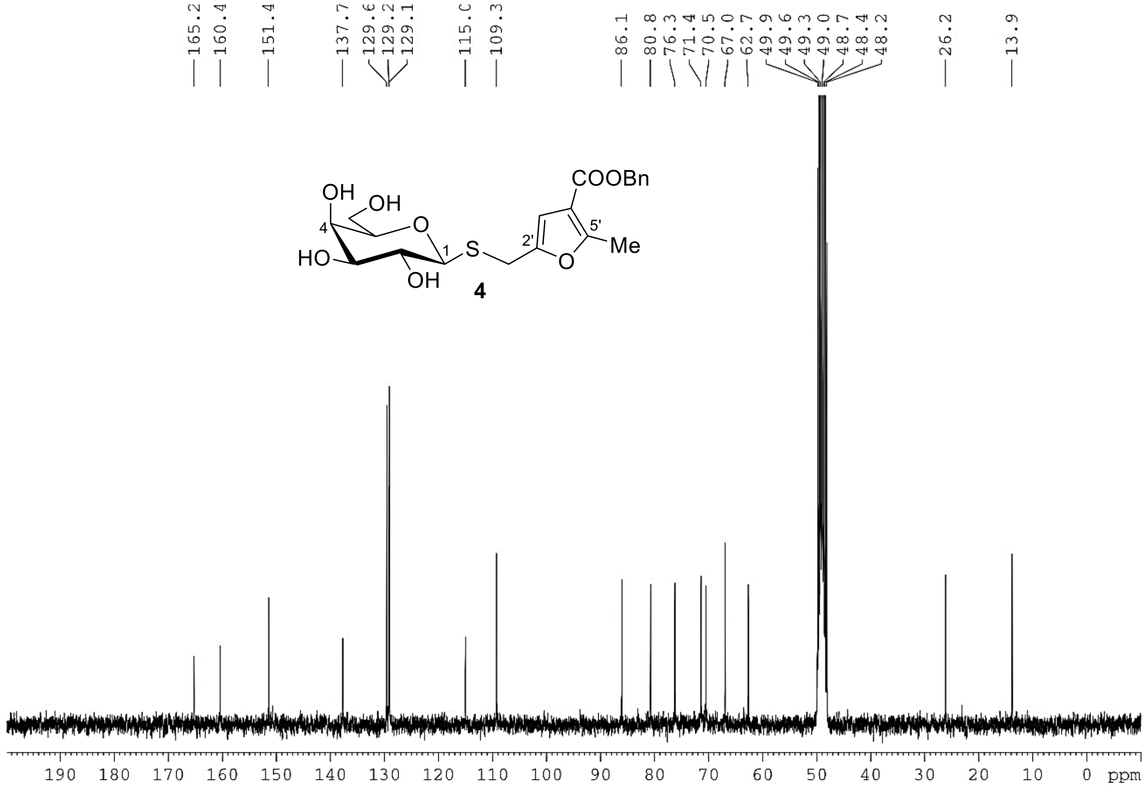

^{13}C NMR (75.4 MHz, CD_3OD)

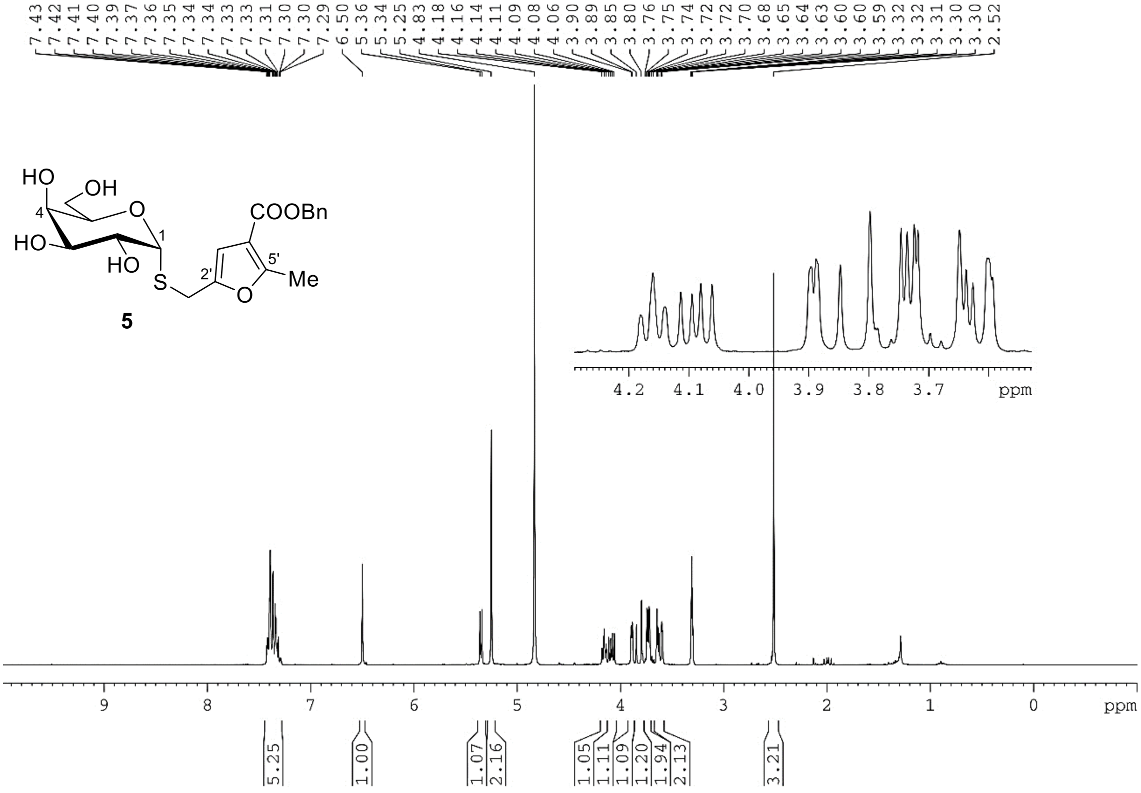

^{1}H NMR (300 MHz, CD_3OD)

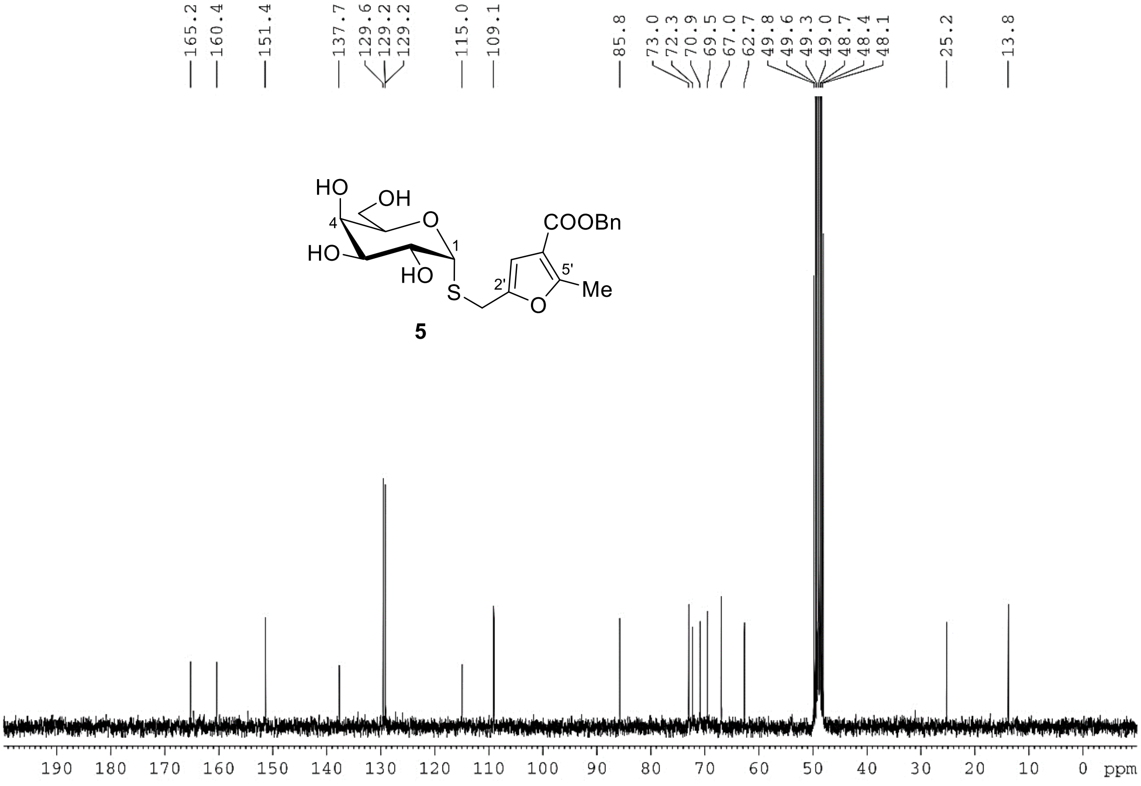

^{13}C NMR (75.4 MHz, CD_3OD)

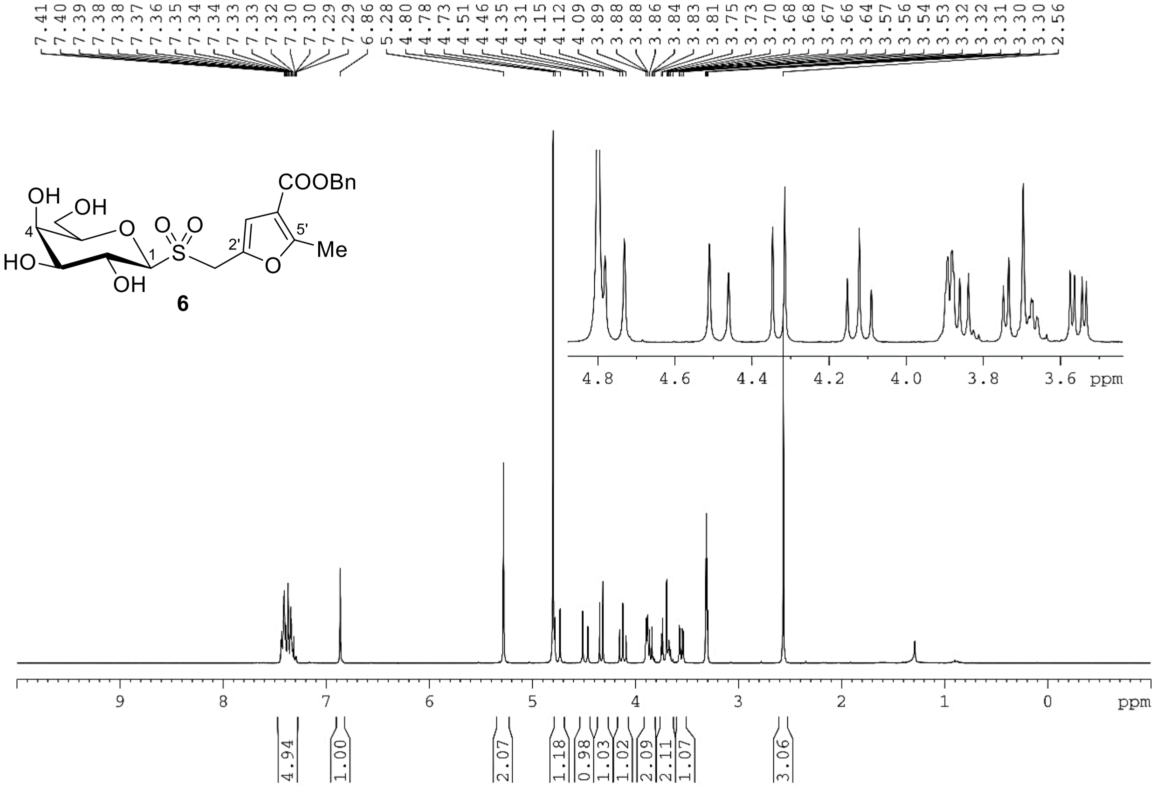

^{1}H NMR (300 MHz, CD_3OD)

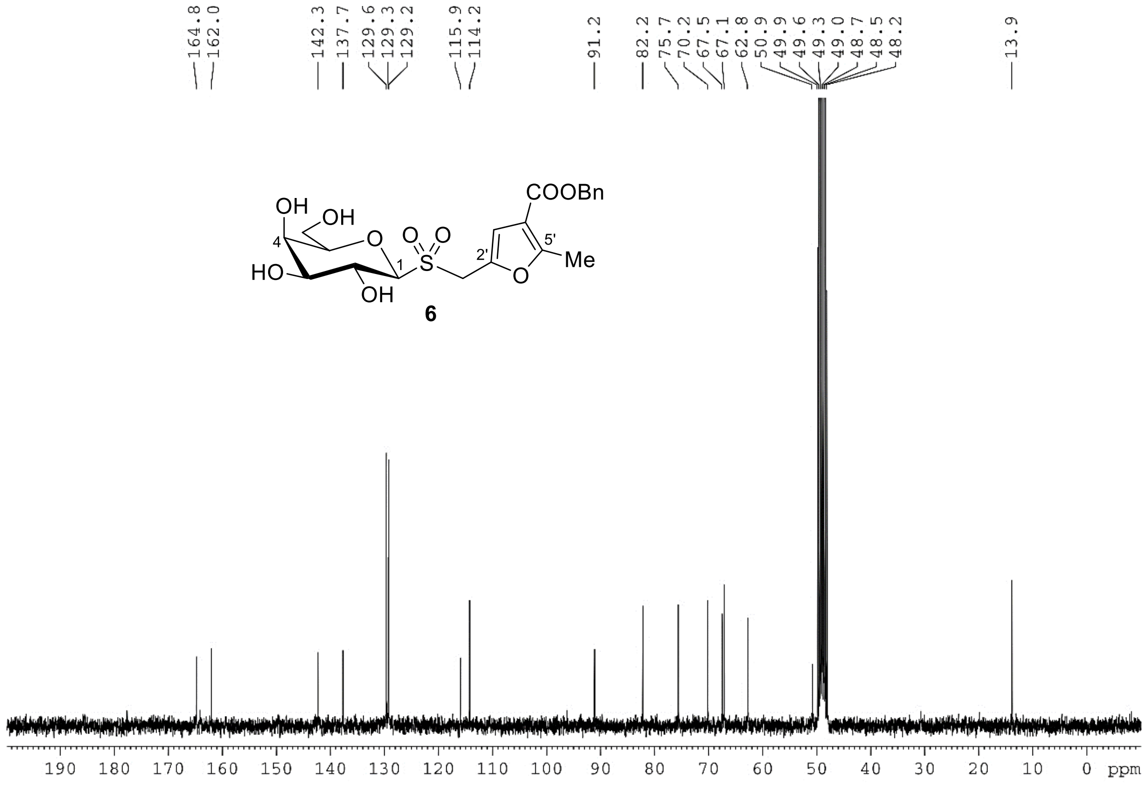

^{13}C NMR (75.4 MHz, CD_3OD)

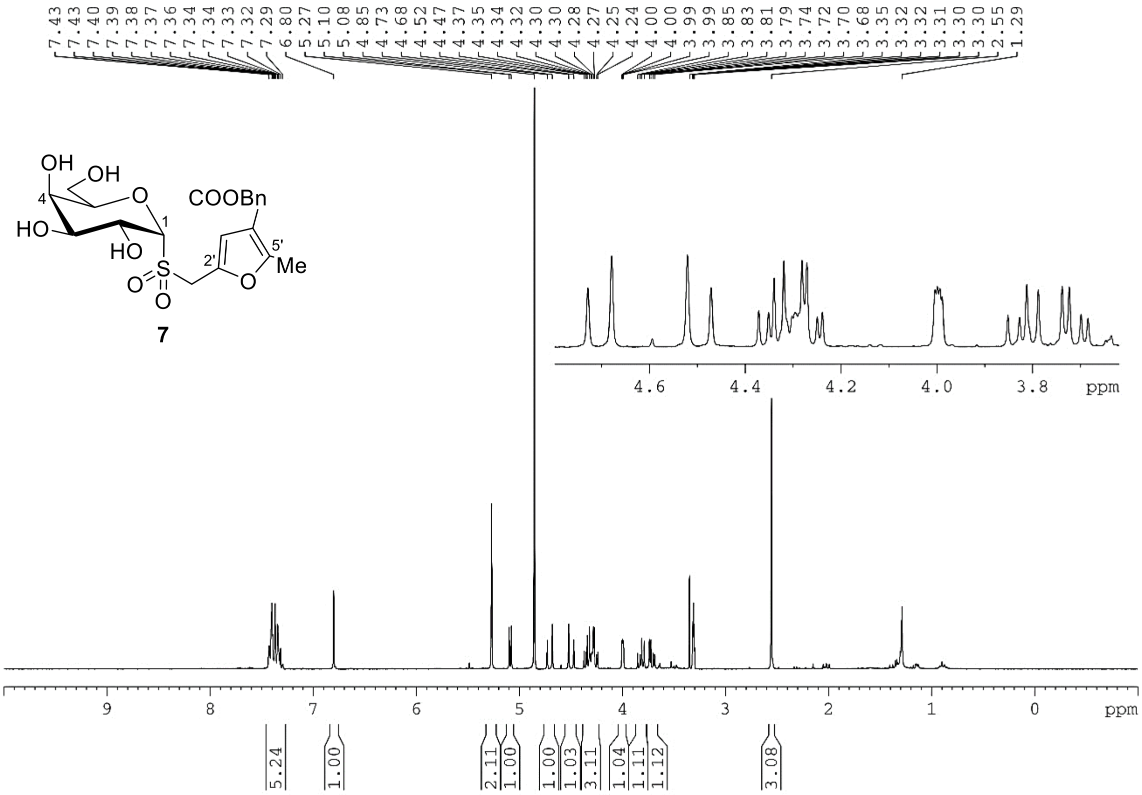

^{1}H NMR (300 MHz, CD_3OD)

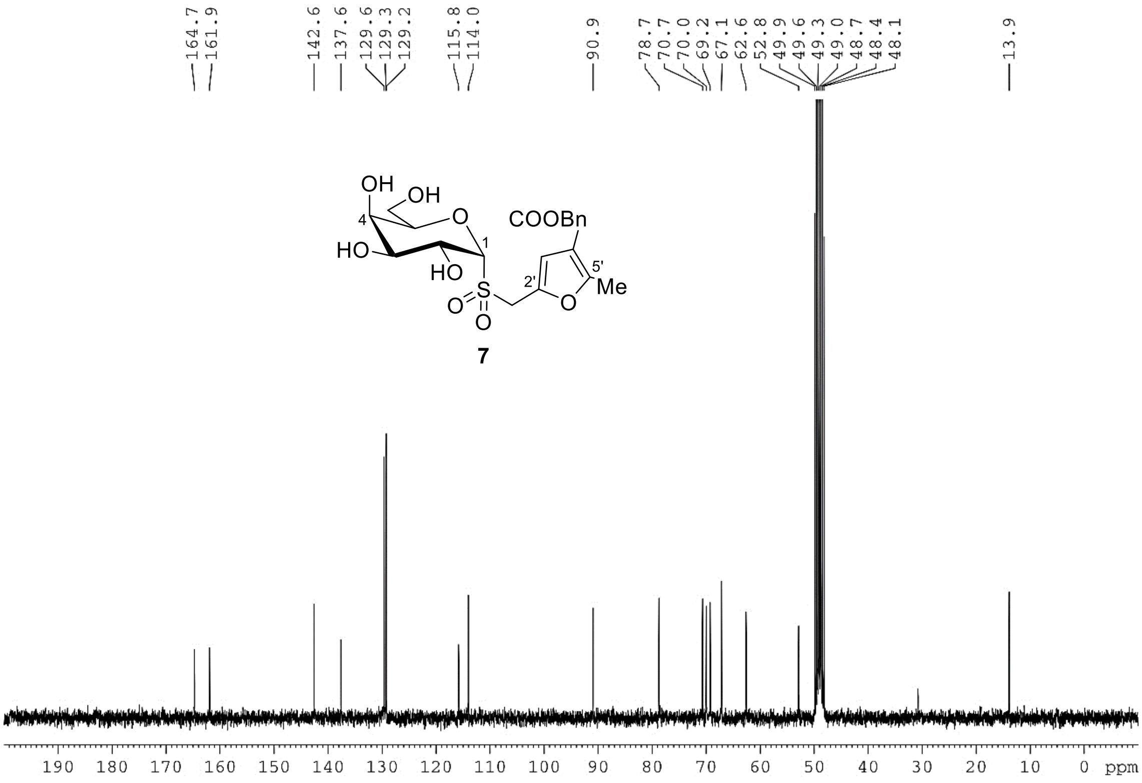

^{13}C NMR (75.4 MHz, CD_3OD)

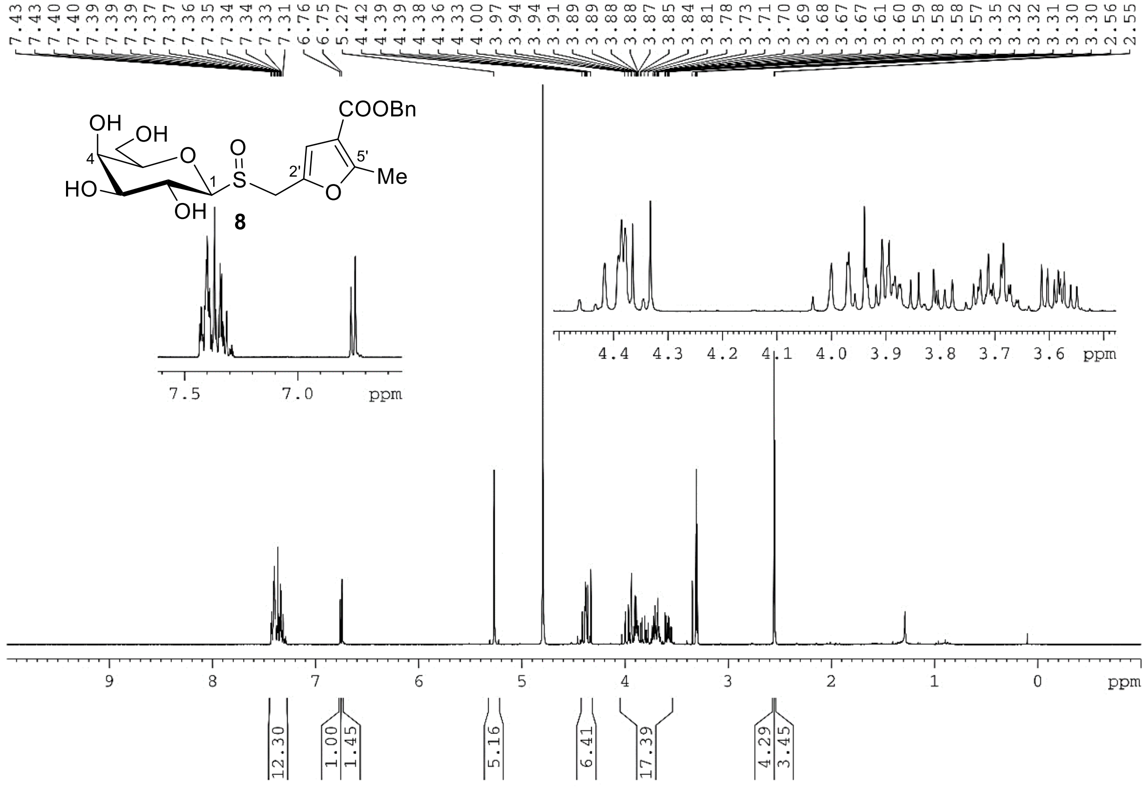

^{1}H NMR (300 MHz, CD_3OD)

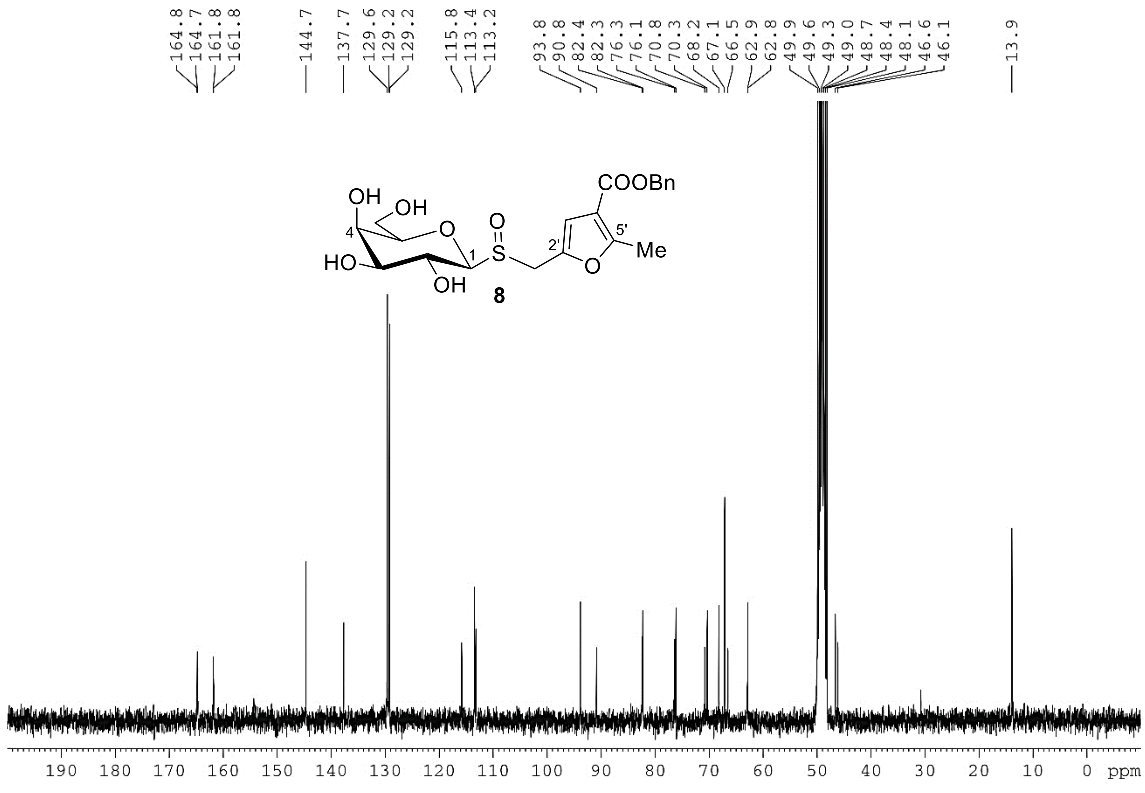

^{13}C NMR (75.4 MHz, CD_3OD)

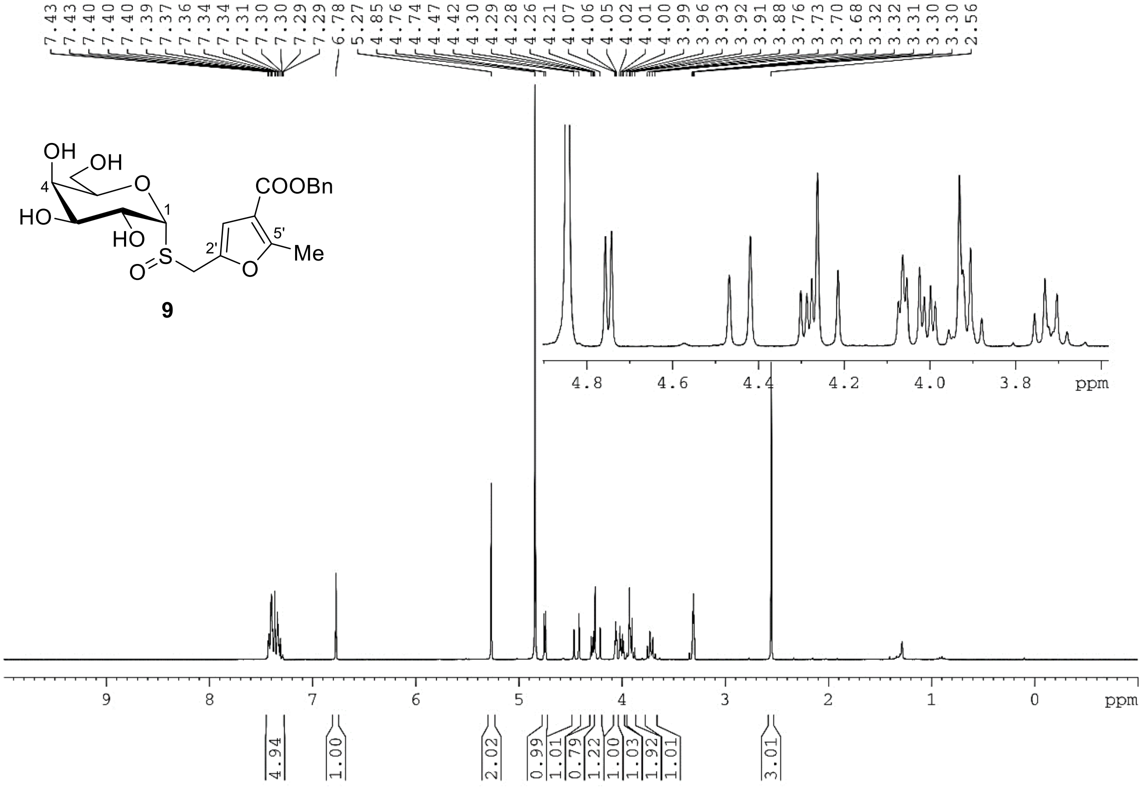

^{1}H NMR (300 MHz, CD_3OD)

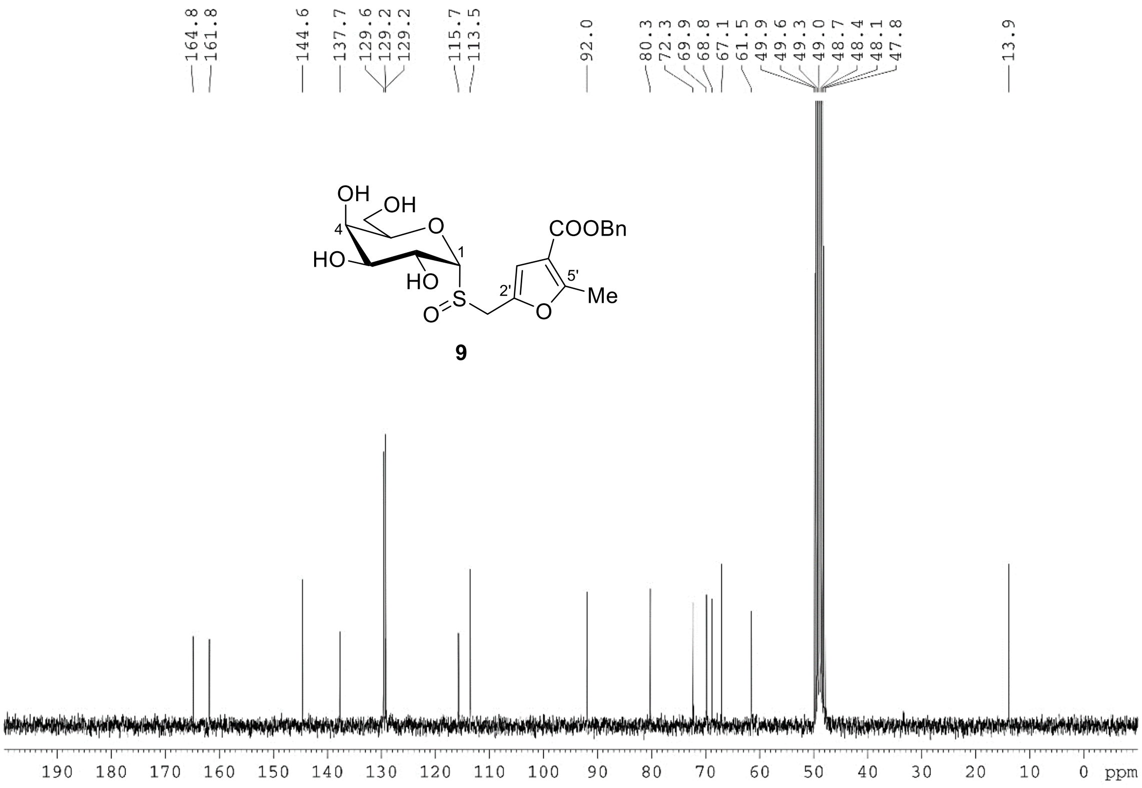

^{13}C NMR (75.4 MHz, CD_3OD)

Binding epitopes of ligands 1, 2, and 3[1]

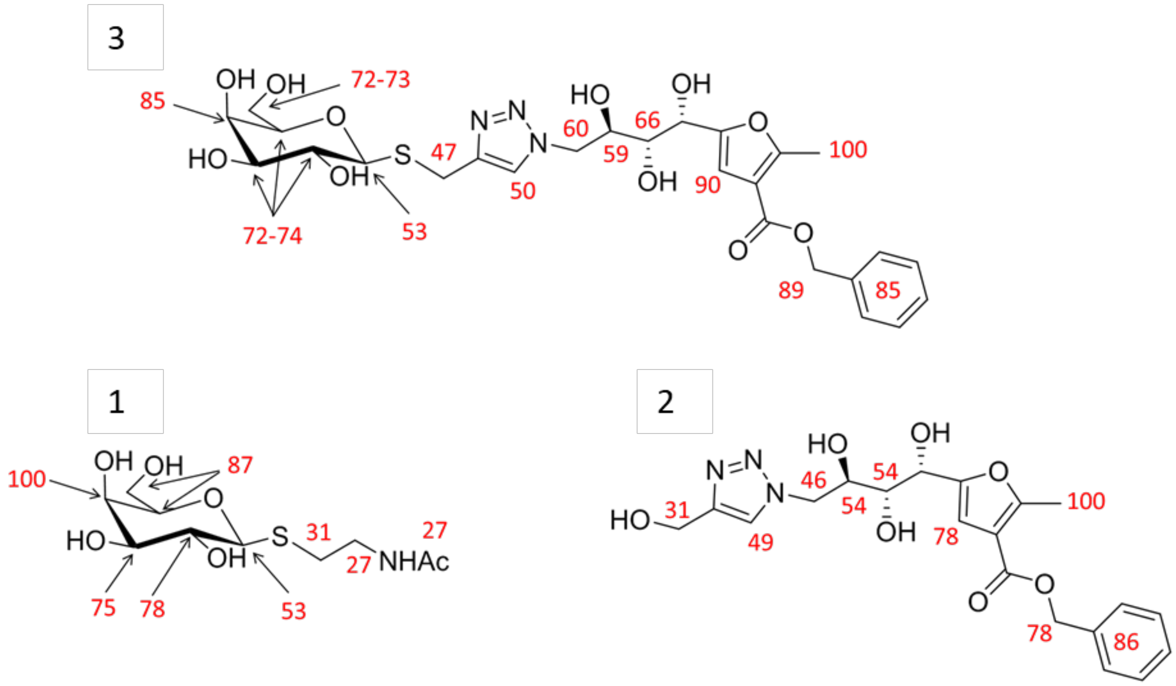

Figure S1. Binding epitopes of ligands **1, 2** and **3** as bound to CTB (from reference [1]). The numbers represent relative values of saturation after their normalization related to the most intense one (assigned 100%), obtained from STD initial slopes (protons nomenclature as in Figure 1 of main text).

Proton assignment of 4-9, raw build-up curves data and normalized STD values

Table S1. ^{1}H and ^{13}C assignment and the raw STD (%) at increasing saturation times. Irradiation frequency 0.0 ppm, 278 K.

			STD (%) Ligand 4							
**Proton ID**	**^{1}H δ (ppm)**	**^{13}C δ (ppm)**	**0.25 s**	**0.5 s**	**0.75 s**	**1 s**	**2 s**	**3 s**	**4 s**	**5 s**
CH_3(fur)	2.22	13.3	2.41	5.51	8.39	10.5	16.6	19.6	21.19	21.82
H2	3.248	68.68	1.92	3.92	7.32	8.98	14.63	18.13	19.22	19.6
H3	3.245	73.45	1.92	3.92	7.32	8.98	14.63	18.13	19.22	19.6
H4	3.277	78.36	1.69	4.54	7.46	8.39	13.4	15.36	16.13	16.77
H6/6'	3.319	60.27	1.71	4.49	6.9	8.39	11.57	13.01	14.07	14.07
CH_2(S)	3.554	25.7	1.83	3.92	8.07	6.08	8.64	10.5	10.91	11.92
H5	3.617	68.3	1.41	4.67	8.07	9.52	15.06	17.44	17.78	19.79
CH_2(S)	3.693	25.7	1.92	2.95	6.64	6.57	8.47	10.5	10.91	11.92
H1	4.039	84.53	1.61	2.41	4.9	6.14	10.81	13.14	14.2	14.91
CH_2(Bn)	5.01	66.01	2.29	4.49	6.44	8.81	12.39	13.27	14.48	13.93
H(fur)	6.279	108.1	1.71	4.54	7.32	9.34	17.1	21.19	22.91	23.83
H para	7.1	127.9	2.14	4.49	7.83	9.8	16.44	20.99	22.47	23.83
H meta	7.14	128.3	1.83	4.36	7.17	9.34	15.81	20.38	22.04	23.83
H orto	7.187	127.9	2.1	4.11	6.51	8.89	15.36	19.22	21.4	22.04

Table S2. ^{1}H and ^{13}C assignment and the raw STD (%) at increasing saturation times. Irradiation frequency 0.0 ppm, 278 K.

			STD (%) Ligand 6							
Proton ID	^{1}H δ (ppm)	^{13}C δ (ppm)	0.25 s	0.5 s	0.75 s	1 s	2 s	3 s	4 s	5 s
CH_3(fur)	2.23	13	6.44	14.48	20.58	25.76	36.6	40.75	42.37	42.79
H2	3.408	72.2	5.96	12.51	18.31	23.36	31.31	35.2	36.25	36.25
H3	3.408	72.2	5.96	12.51	18.31	23.36	31.31	35.2	36.25	36.25
H6	3.446	60.8	6.14	12.63	18.67	22.25	28.12	31.71	31.93	31.93
H6'	3.531	80.1	6.08	12.51	19.22	21.61	28.12	29.53	30.71	30.11
CH_2(S)	3.531	60.8	6.38	13.15	18.13	21.81	27.58	29.81	30.41	30.41
H5	3.692	68.1	6.26	12.51	19.79	23.59	32.88	36.25	34.18	36.6
H4	3.82	64.9	5.04	13.14	18.85	23.59	38.43	46.26	44.06	46.71
H1	4.236	88.3	3.84	9.9	14.91	19.6	29.82	33.85	35.5	35.5
CH_2(Bn)	4.984	66.3	6.2	13.14	17.95	22.35	29.53	31.62	32.24	33.52
H(fur)	6.607	112.9	5.79	12.63	19.6	25.51	41.15	48.1	51	52.2
H para	7.085	128.15	5.9	13.14	20.58	25.76	39.57	45.37	47.17	48.57
H meta	7.119	128.4	5.73	13.66	20.58	25.76	39.57	45.37	47.17	48.57
H orto	7.168	127.8	5.62	12.88	19.79	24.74	38.43	44.06	46.26	47.63

Table S3. ^{1}H and ^{13}C assignment and the raw STD (%) at increasing saturation times. Irradiation frequency 0.0 ppm, 278 K.

			STD (%) Ligand 8							
Proton ID	^{1}H δ (ppm)	^{13}C δ (ppm)	0.25 s	0.5 s	0.75 s	1 s	2 s	3 s	4 s	5 s
CH_3(fur)	2.24	12.9	4.4	9.52	13.53	17.27	25.76	29.24	30.41	31.31
H6	3.44	60.83	3.04	7.53	11.35	14.07	20.18	21.61	22.47	22.25
H6'	3.541	60.83	3.32	6.57	9.61	13.14	16.13	16.77	17.61	17.95
H5	3.545	80.2	3.32	6.57	9.61	13.14	16.13	16.77	17.61	17.95
H2	3.682	66.27	3.01	6.57	10	13.66	20.99	23.59	26.27	26.27
H3	3.682	64.4	3.01	6.57	10	13.66	20.99	23.59	26.27	26.27
H4	3.829	87.9	4.11	7.91	14.77	17.27	25.26	28.12	29.1	29.82
CH_2(S)	4.14	44.09	3.13	6.2	6.9	8.98	13.93	14.34	15.36	15.36
H1	4.19	90.7	2.58	5.79	8.14	10	15.21	17.61	17.78	17.78
CH_2(S)	4.21	44.09	2.58	5.79	8.14	10	15.21	17.61	17.78	17.78
CH_2(Bn)	5.105	66.3	3.8	7.32	10.7	13.53	18.67	19.41	20.99	20.58
H(fur)	6.51	112.2	3.96	8.31	13.1	16.28	27.05	31.93	34.18	34.86
H para	7.103	128.13	3.99	7.83	11.69	15.06	25.76	31.01	32.88	34.18
H meta	7.14	128.39	3.38	7.53	11.69	15.51	25.76	31.01	32.88	34.18
H orto	7.189	127.83	3.32	7.32	11.24	14.48	24.29	29.53	31.31	32.24

Table S4. ^{1}H and ^{13}C assignment and the raw STD (%) at increasing saturation times. Irradiation frequency 0.0 ppm, 278 K.

			STD (%) Ligand 5							
**Proton ID**	**^{1}H δ (ppm)**	**^{13}C δ (ppm)**	**0.25 s**	**0.5 s**	**0.75 s**	**1 s**	**2 s**	**3 s**	**4 s**	**5 s**
CH_3(fur)	2.217	13	5.3	11.02	15.97	20.99	31.01	34.52	35.89	35.2
H6	3.379	60.5	5.05	8.39	14.63	16.28	19.79	20.69	21.4	21.4
H3	3.394	69.9	5.05	8.39	14.63	16.28	19.79	20.69	21.4	21.4
H6	3.417	60.5	3.77	7.68	12.63	15.06	21.19	21.19	21.4	21.82
CH_2(S)	3.433	24.3	3.77	7.68	12.63	15.06	21.19	21.19	21.4	21.82
CH_2(S)	3.526	24.3	4.19	5.41	9.25	11.24	15.97	16.77	17.2	17.95
H5	3.654	68.6	4.54	8.07	13.53	17.27	24.77	27.58	28.96	28.96
H2	3.767	67.3	4.32	8.07	12.15	13.66	25.26	31.31	30.41	32.28
H4	3.925	71.2	5.84	8.31	13.14	18.13	26.01	28.12	29.4	30.11
CH_2(Bn)	5.006	66.2	3.45	8.31	10.7	13.66	22.04	23.14	24.29	23.59
H1	5.09	83.9	3.66	7.56	9.9	13.66	22.47	26.52	26.52	27.85
H(fur)	6.27	107.2	3.99	9.25	15.06	18.85	29.82	35.55	37.32	37.69
H para	7.107	128.14	2.87	7.39	11.57	14.48	23.83	30.41	30.71	33.2
H meta	7.143	128.32	2.76	7.24	12.03	14.91	24.53	30.41	32.88	33.85
H orto	7.182	128.32	3.07	7.39	11.69	14.2	24.77	30.41	32.56	33.2

Table S5. ^{1}H and ^{13}C assignment and the raw STD (%) at increasing saturation times. Irradiation frequency 0.0 ppm, 278 K.

			STD (%) Ligand 7							
**Proton ID**	**^{1}H δ (ppm)**	**^{13}C δ (ppm)**	**0.25 s**	**0.5 s**	**0.75 s**	**1 s**	**2 s**	**3 s**	**4 s**	**5 s**
CH_3(fur)	2.23	13.2	2.81	6.2	9.52	12.3	19.41	22.69	24.53	25.02
H6	3.408	60.6	4.15	7.83	10.5	14.2	18.67	17.27	18.85	18.85
H6'	3.492	60.6	4.49	8.89	12.27	15.06	19.22	18.13	19.99	19.99
H4	3.574	77.9	3.59	7.53	11.13	13.27	18.85	21.19	21.19	21.19
H5	3.764	67.6	2.48	6.44	9.71	13.27	20.58	23.83	24.77	26.01
H3	3.764	70.3	2.48	6.44	9.71	13.27	20.58	23.82	24.77	26.01
CH_2(S)	4.12	44.6	2.68	4.36	7.1	8.14	12.15	13.86	13.4	13.52
H2	4.064	67.3	3.07	5.31	10.5	11.27	19.99	23.83	24.77	25.01
CH_2(S)	4.126	44.6	2.68	4.4	7.1	8.39	11.92	12.35	13.01	11.35
H1	4.468	92.3	3.35	2.89	6.77	9.71	15.21	17.61	19.22	19.41
CH_2(Bn)	5.006	66.3	2.12	5.68	7.99	10.09	14.77	16.44	16.6	17.1
H(fur)	6.528	112.67	2.45	6.64	9.8	13.14	22.91	28.4	30.71	31.93
H para	7.104	128.1	2.68	6.2	9.43	12.39	21.4	26.27	28.68	30.41
H meta	7.14	128.37	2.68	5.9	9.16	12.03	20.78	26.27	28.68	30.41
H orto	7.195	127.7	2.38	5.57	8.81	11.46	19.6	24.53	27.05	28.4

Table S6. ^{1}H and ^{13}C assignment and the raw STD (%) at increasing saturation times. Irradiation frequency 0.0 ppm, 278 K.

			STD (%) Ligand 9							
Proton ID	^{1}H δ (ppm)	^{13}C δ (ppm)	0.25 s	0.5 s	0.75 s	1 s	2 s	3 s	4 s	5 s
CH_3(fur)	2.2	12.8	3.99	6.08	7.68	12.39	14.77	16.44	17.1	17.27
H4	3.37	74.9	1.96	2.65	3.62	5.3	5.79	7.04	7.24	6.83
H6/6'	3.422	60.8	3.59	4.32	5.57	7.99	8.64	9.25	9.34	8.89
H5	3.735	67.9	3.73	5.73	7.1	12.51	13.93	13.53	15.06	13.93
H3	3.945	76.7	3.29	4.49	4.76	10.81	10.7	11.8	13.01	11.8
H2	4.05	66.6	3.79	4.28	4.76	10.19	10.5	14.2	14.07	14.34
CH_2(S)	4.309	51.5	0.75	1.45	1.85	2.6	3.22	4.49	4.11	4.95
CH_2(S)	4.43	51.5	-	-	-	-	-	-	-	-
CH_2(Bn)	4.97	66.4	3.29	4.49	6.2	9.43	10.91	11.46	11.92	11.24
H1	4.97	89.2	3.29	4.49	6.2	9.43	10.91	11.46	11.92	11.24
H(fur)	6.53	113.27	4.28	6.2	8.31	15.66	20.39	23.14	23.83	23.59
H para	7.066	128.11	3.32	4.76	6.83	11.46	15.06	17.1	17.95	17.44
H meta	7.098	128.55	3.45	5.41	7.39	12.51	15.66	17.95	18.85	19.41
H orto	7.14	127.7	3.1	5.1	6.7	12.03	15.66	17.1	18.13	18.31

Table S7. STD NMR binding epitope mapping ligands **4-9**. Normalised initial slope values for ligands **4-9**, obtained from the fitting of the raw data in the Tables S1 to S6. For each ligand, the epitope is normalised against the proton with the strongest initial slope, arbitrarily.

	Normalised initial slopes %					
	Ligand 4	Ligand 5	Ligand 6	Ligand 7	Ligand 8	Ligand 9
**H1**	57	62	71	54	60	76
**H2**	85	67	90	73	72	67
**H3**	85	91	90	79	72	73
**H4**	85	84	90	90	100	41
**H5**	90	80	96	79	77	94
**H6**	83	91	94	100	83	76
**H6'**		78	96		77	
**CH_2(S)**	59	78	96	56	59	17
**CH_2(S)**	60	56		62	60	
**H(fur)**	90	86	91	77	91	100
**CH_3 (fur)**	100	100	100	73	99	89
**CH_2(Bn)**	87	70	93	65	82	76
**H orto**	81	66	91	65	79	78
**H meta**	84	66	95	71	84	81
**H para**	90	65	95	73	84	76

DEEP-STD NMR experiments for ligands 4-7 and 3NPG in complex with CTB

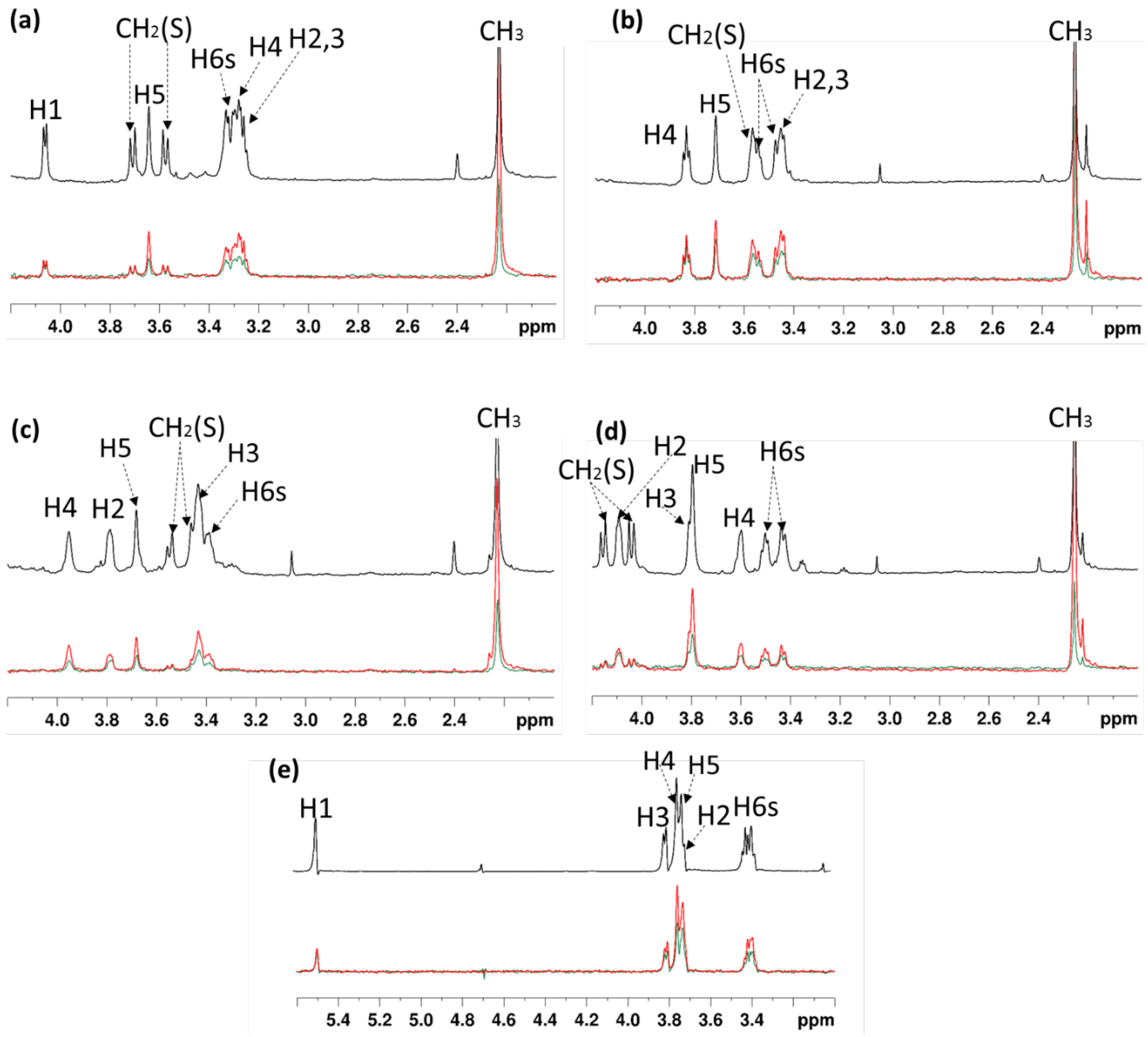

Figure S2. DEEP-STD NMR (2.25ppm/0.60ppm) spectra of ligand (a) **4**, (b) **5**, (c) **6**, (d) **7** and (e) 3NPG in complex with CTB, at 2 s saturation time. In each panel, the reference spectrum (x1) is in black, and the difference spectra (x2) are in red (irradiation at 2.25 ppm) and green (irradiation at 0.60 ppm). Only the galactose spectral region is shown, and the assignment of each peak is shown on top.

Competition experiments: STD NMR spectra

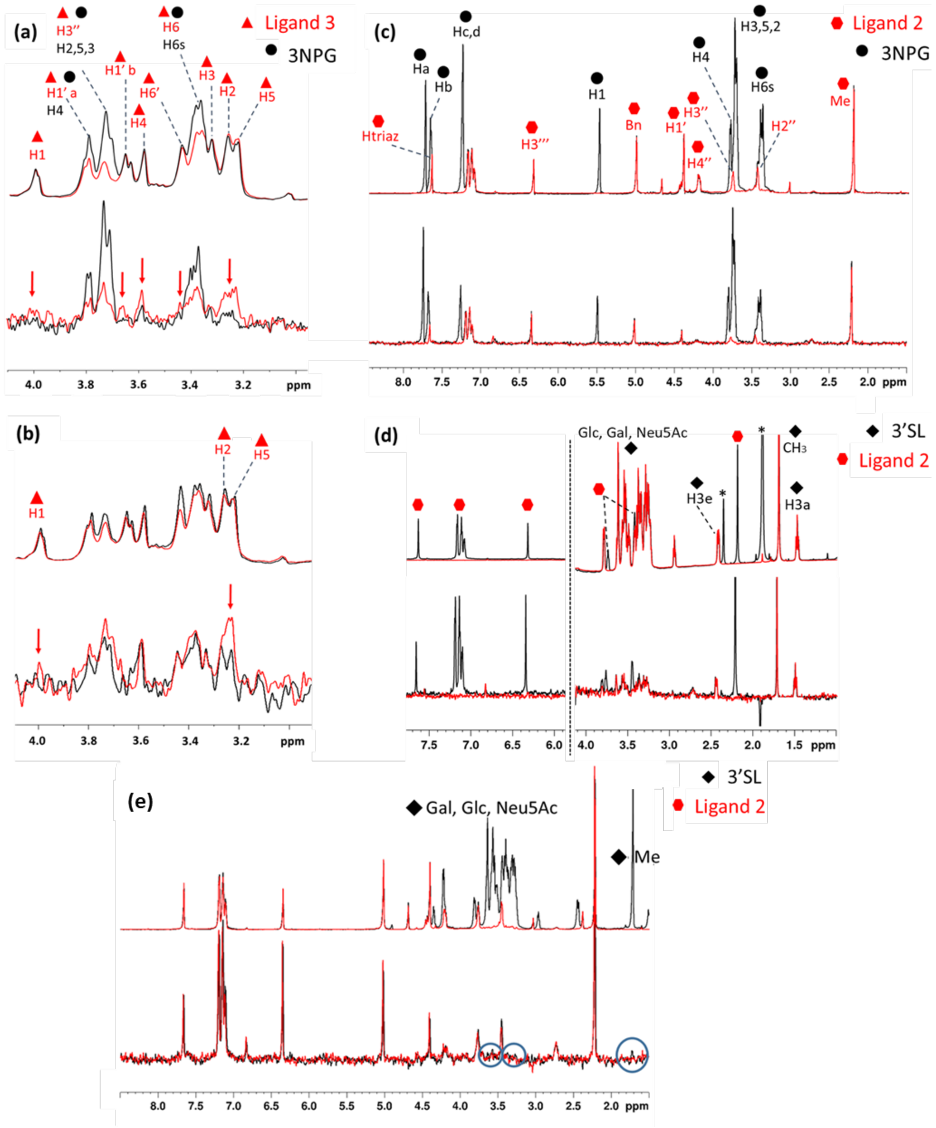

Figure S3. STD NMR competition experiments (a), (b), (c) and (d) (see also Table 2), and control experiment (e). For each panel a superposition of reference spectra (top) and a superposition of STD spectra (bottom) are shown (reporter ligands alone in red and upon addition of the competitor in black). The assignment (see Figures 1 and 2 in the main text for nomenclature) is given when needed for the signals of ligand **3** (red triangle), 3NPG (black circle), ligand **2** (red hexagon) and 3'SL (black diamond). Red arrows indicate decrease of STD signals upon addition of the competitor. **a)** Galactose STD signals of ligand **3** decrease upon addition of 3NPG (complete spectra with assignments in Figure S4). b) STD signals of ligand **3** decrease upon addition of ligand **2**. Only protons H5, H2 and H1 can be monitored due to overlapping of the non-galactose peaks of two ligands (see panel a) for assignment and Figure S4 for complete reference spectra). c) STD signals of ligand **2** are not affected upon addition of 3NPG. d) STD signals of 3'SL are not affected upon addition of ligand **2**. e) STD signals of ligand **2** are not affected upon addition of 3'SL. The STD signals of 3'SL can be seen in the baseline of the difference (see panel c) for assignment of Ligand **2** signals; see also Figure S5).

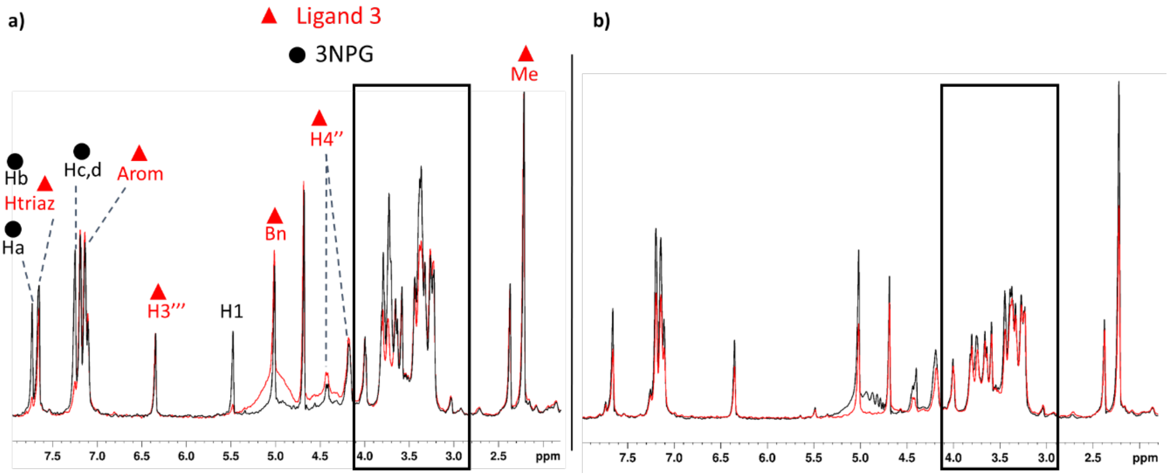

Figure S4. Full width reference spectra of the STD competition experiments **a)** ligand **3** + 3NPG and **b)** ligand **3** + ligand **2** with CTB. In red the spectra of the first binder alone and in black the spectra upon addition of the competitor. The assignment of 3NPG and ligand **3** is given in **a)** (except for the 3.0 ppm to 4.0 ppm region squared in black, assigned in the Figure 3 of main text), with signals belonging to ligand **3** and 3NPG marked with a red triangle and a black circle, respectively. The peaks of ligand **2** perfectly overlap to those of ligand **3**, therefore in b) the assignment is not repeated. A line broadening factor of 3 Hz was applied to the FID before FT.

STD NMR experiment on the 3'SL/CTB binary complex

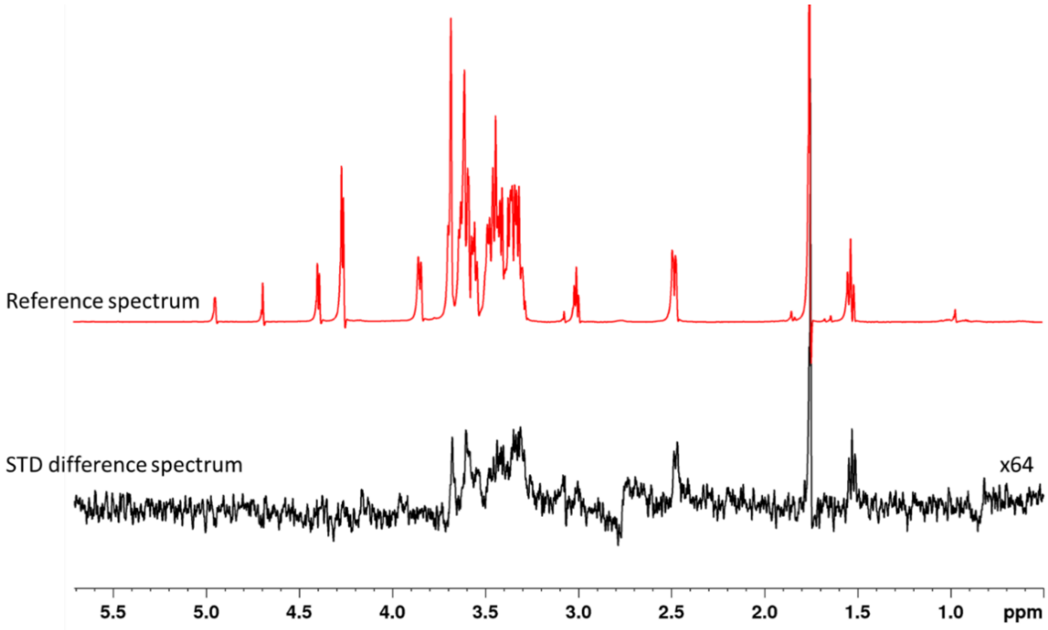

Figure S5. 25 μM CTB in the presence of 1 mM of 3'SL, 278 K. The reference and the difference spectra are shown and labelled; the magnification of the difference spectra relative to the reference spectra is reported. Spectra are acquired at 2 s saturation time and 0.0 ppm saturation frequency. A line broadening factor of 3 Hz was applied to the FID before FT.

Control tr-NOESY spectra

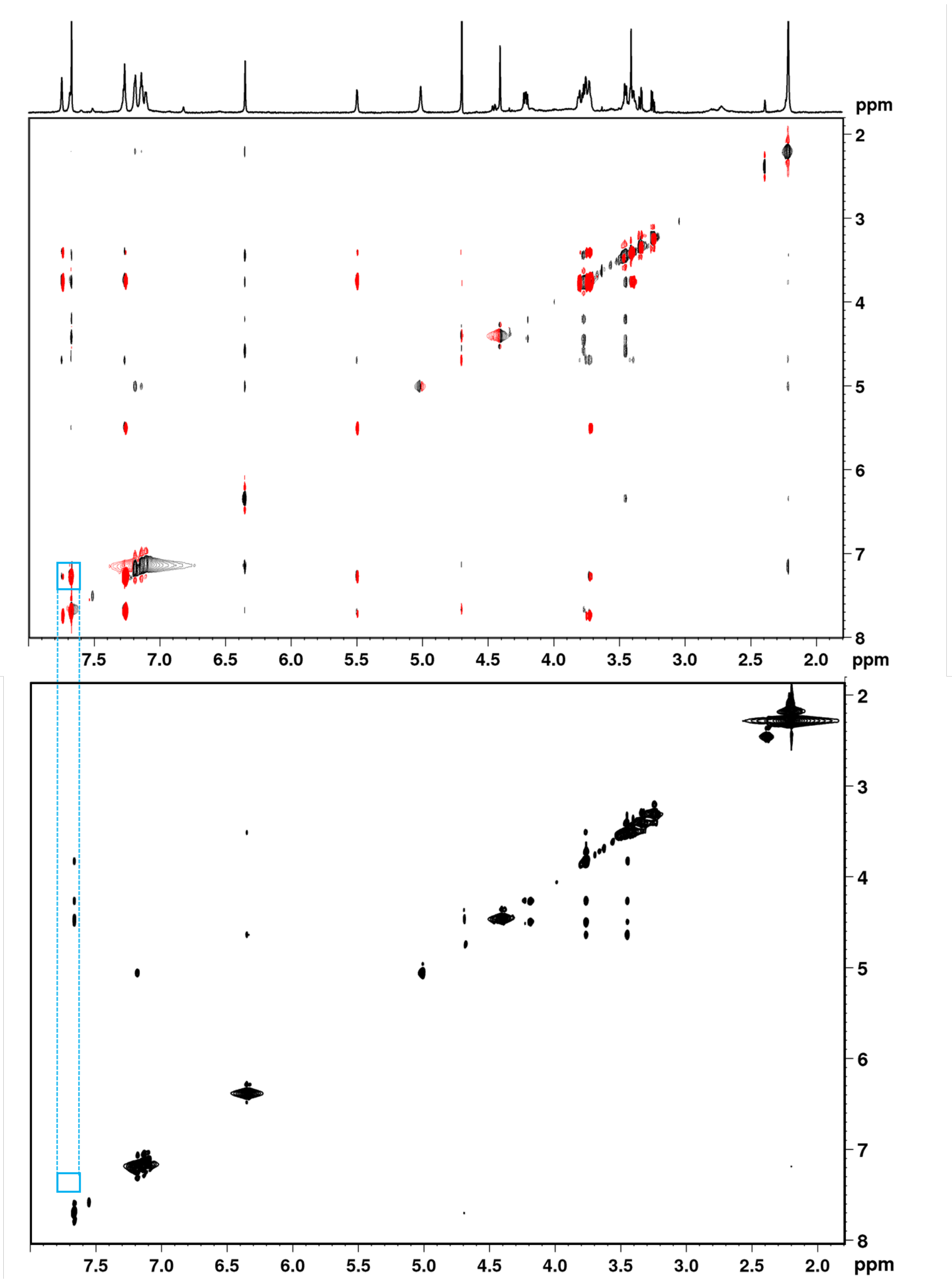

Figure S6. Full spectral width tr-NOESY experiments (mixing time 1.2 s). Top, **2**/CTB/3NPG complex (ternary complex, in black) and 3NPG/CTB complex (binary complex, in red), as in Figure 7 of main text. Bottom, **2**/CTB complex (binary complex): the spectral area containing the ILOE spectra is empty when the protein is alone, confirming that the ILOE is due to the spatial proximity of the ligands in the ternary complex.

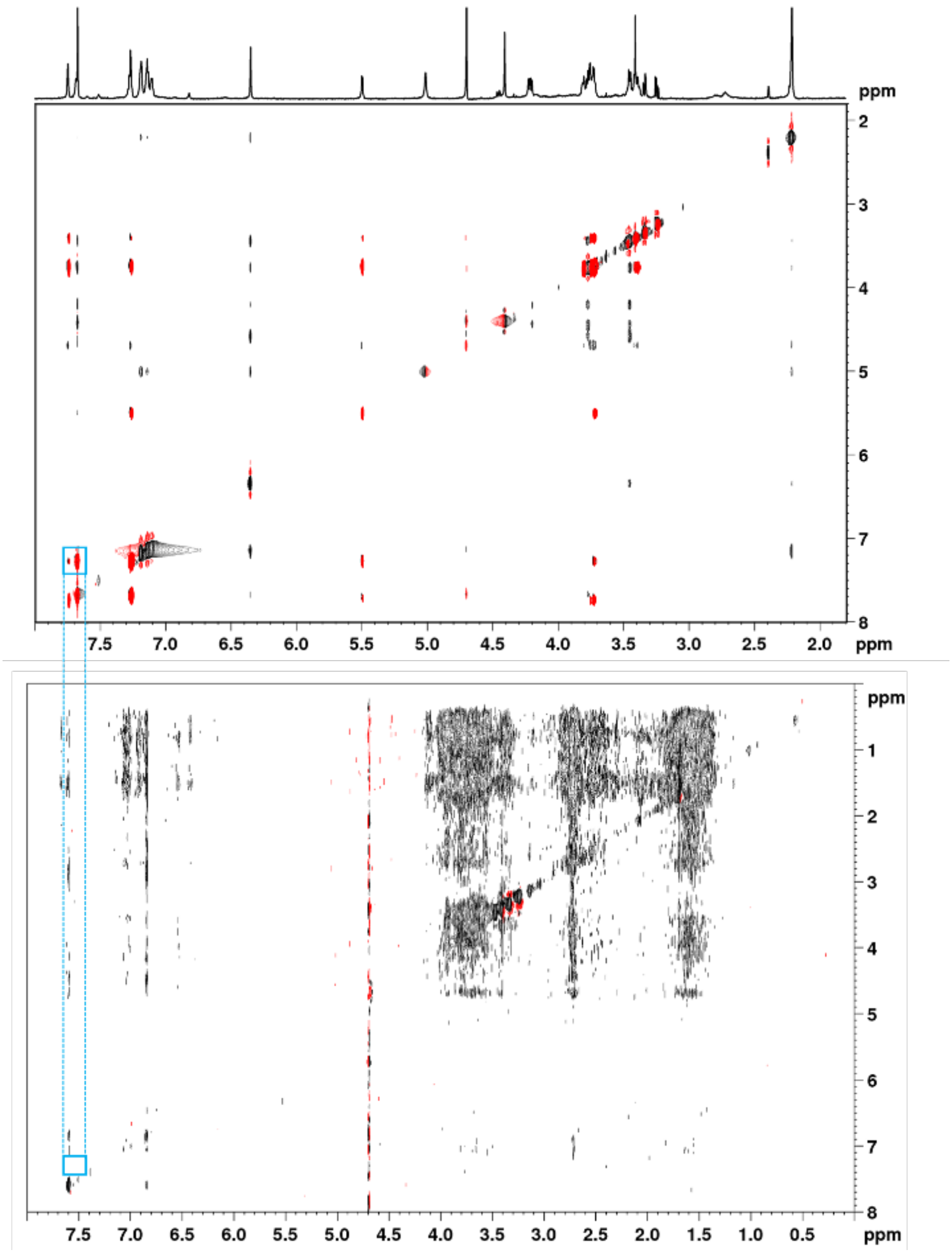

Figure S7. Full width tr-NOESY experiments (Mixing time 1.2 s). Top, **2**/3NPG/CTB complex (ternary complex, in black) and 3NPG/CTB (binary complex, in red), as in Figure 7 of main text. Bottom, CTB alone: the spectral area containing the ILOE spectra is empty when the protein is alone, confirming that the ILOE is due to the ligands**.**

Rigid molecular docking of ligand 1.

Based on the available experimental data, molecular docking of ligands **1**, **2** and **3** was undertaken to provide a 3D model for binding. The coordinates of CTB from the X-ray structure in complex with GM1 (PDB ID: 3CHB) were used to generate the receptor grid and the docking parameters were optimized by re-docking GM1 to reproduce the crystal structure (Figure S8a).

With this setting, docking of ligand **1** showed the thio-galactoside moiety fitting in the galactose subsite with similar orientation as the galactose ring of GM1 (Figure S8b). The docking model was validated against the available STD NMR build-up curves by predicting STD intensities using CORCEMA-ST[3]. Predicted STD intensities are compared to experimental NMR data through the so-called NOE R-factor, with a low value indicating a good matching.

$$NOE\ R-Factor = \sqrt{\frac{\sum_k \left(STD_k^{exp} - STD_k^{cal}\right)^2}{\sum_k \left(STD_k^{exp}\right)^2}}$$

In our experience, values ≤0.3 report on good agreement between the 3D model and the experimental data. Docking model of ligand **1** showed an excellent NOE R-factor of 0.22 (Figure S9)

Ligand 1 docking solution and CORCEMA-ST validation

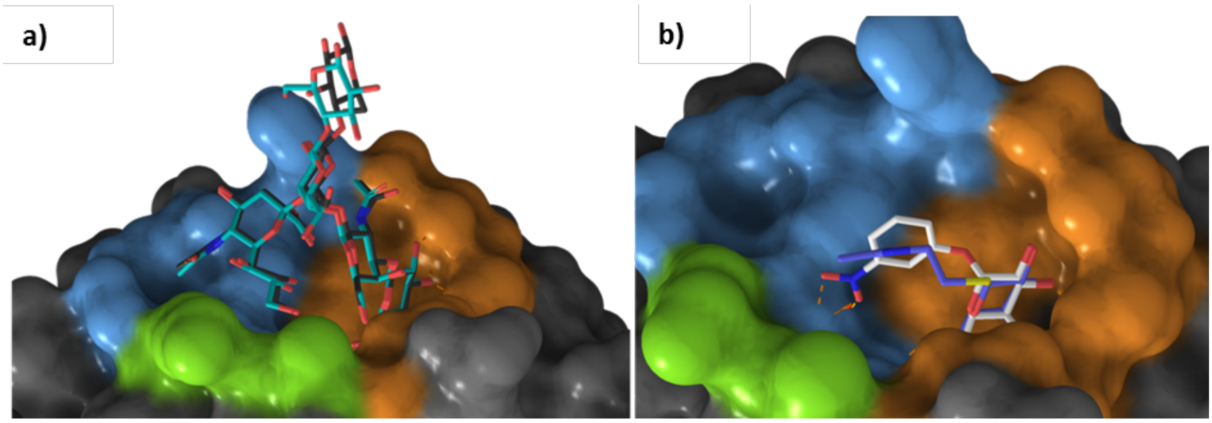

Figure S8. a) Lowest energy docking solution for GM1 (carbon atoms in turquoise) on CTB superimposed to the XRD structure of the same complex (carbon atoms in dark grey, PDB ID: 3CHB): with optimised docking conditions, very good convergence is observed. **b)** Lowest energy docking solution for ligand **1** (purple) on CTB superimposed to the XRD structure of 3NPG/CTB complex. All ligand protons are omitted for clarity.

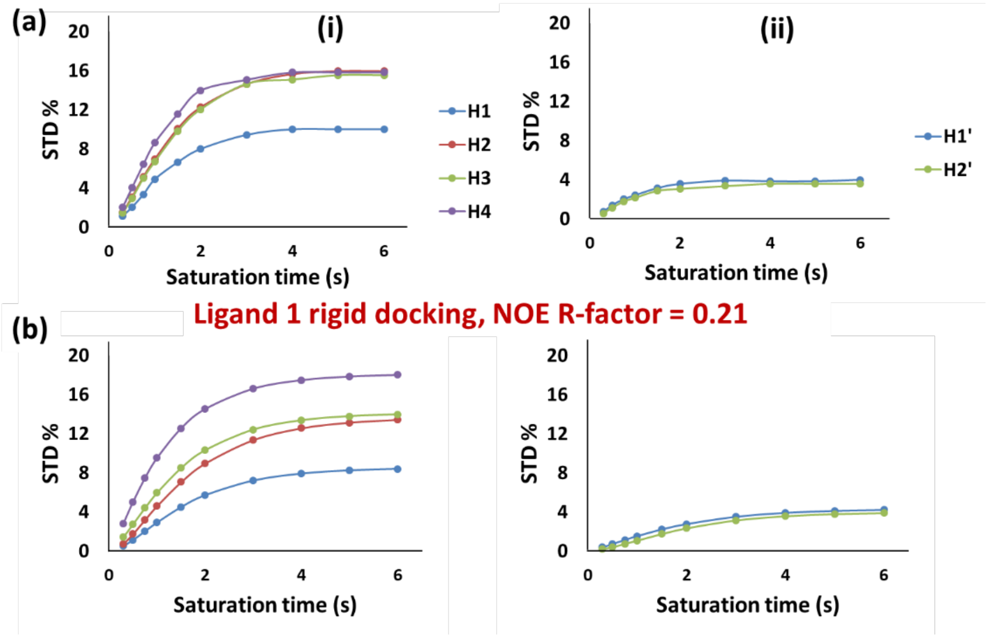

Figure S9. Ligand **1**, CORCEMA-ST calculations on rigid docking solutions. a) Experimental STD intensities at increasing saturation time (build-up curves) for ligand **1** in complex with CTB[1]. b) CORCEMA-ST predicted STD intensities using the coordinates of the 3D model from the lowest energy docking solution of the complex (see Figure 1 in the main text for protons nomenclature).

Ligands 2 and 3 rigid docking solutions

Ligand **3** docked consistently fitting the thio-galactose in the corresponding galactose subsite. Superposition of the galactose moiety of the lowest energy solution with the validated structure of ligand **1** showed convergence for the orientation of the thio-galactosidic linkage, with triazole of ligand **3** oriented similarly to the acetamide group of ligand **1** (Figure S10a). On the contrary, the furoate-benzyl moiety at the other end of **3** failed to converge to a well-defined solution, sampling the whole surface around the galactose subsite and mostly hindering the sialic acid subsite (Figure S10b), in disagreement with the competition experiments (Main Text Table 2).

CORCEMA-ST model validation was possible only for atoms up to the triazole, while validation for the furoate-benzyl moiety failed for all the docking solutions generated (Figure S11).

The docking solutions for ligand **2** failed to converge at all, neither with other ligand **2** solutions nor with the PHF moiety of ligand **3** (Figures S10c and S10d). Most solutions occupied both the galactose and sialic acid subsites, showing the inability of rigid docking to describe this system (Figure S12).

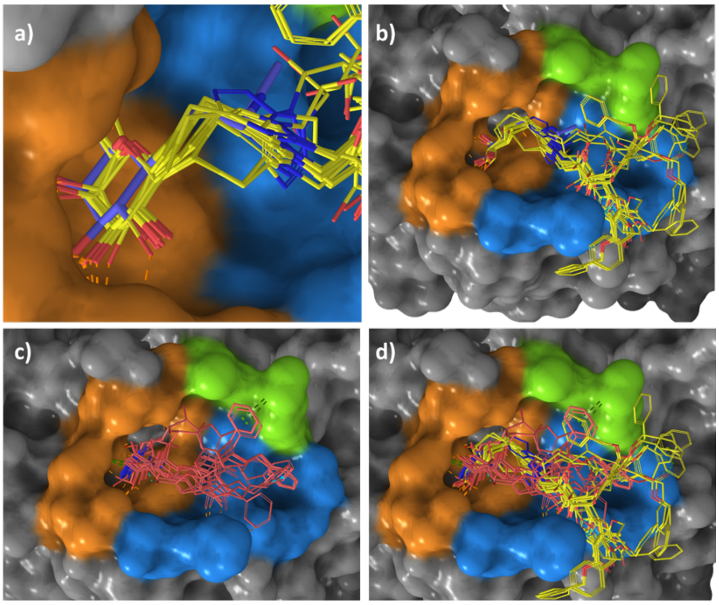

Figure S10. a) Zoom on the galactose sub-site: the 15 lowest energy docking solutions for ligand **3** (yellow) as superimposed to the lowest energy docking solution of ligand **1** (purple); b) similar zoom as in a) now covering the entire GM1 binding pocket. c) The 15 lowest energy docking solution for ligand **2** (light red); d) superimposition of b) and c). In this docking models, occupation of the sialic binding subsite (blue) by both ligands disagrees with the experimental competition data.

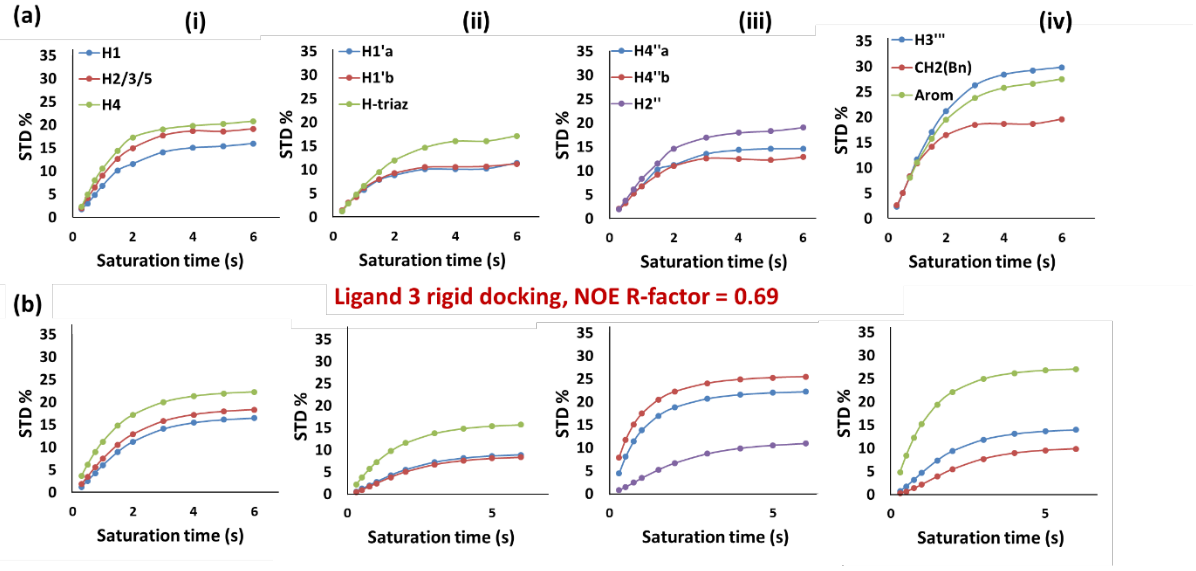

Figure S11. Ligand **3**, CORCEMA-ST calculations on rigid docking solutions. a) Experimental STD intensities at increasing saturation time (build-up curves) for ligand **3** in complex with CTB[1]. b) CORCEMA-ST calculated STD intensities for the lowest energy docking solution of the complex. Remarkably, the calculated data for the thio-galactose moiety (occupying the galactose sub-site in the docking solution, see Figures S10a and S10b) in (i) and (ii) fit well the experimental data, whereas the calculated data for the rest of the molecule in (iii) and (iv) did not fit the experimental results; this part of the molecule occupies the sialic acid binding sub-site in the docking solution (see Figure S10c and Figure 1 in the main text for protons nomenclature).

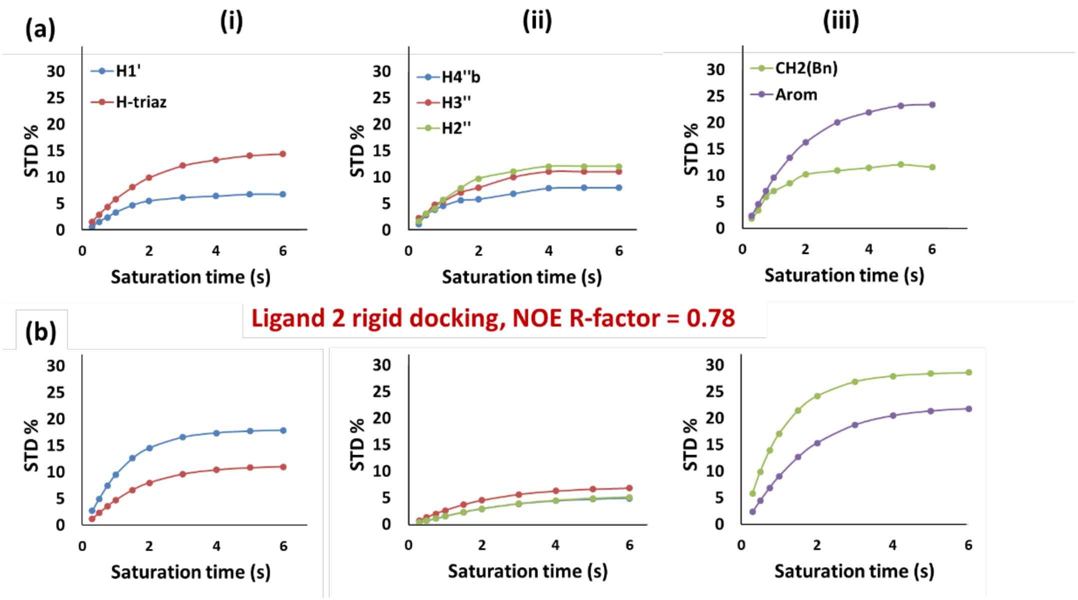

Figure S12. Ligand **2**, CORCEMA-ST calculations on rigid docking solutions. a) Experimental STD intensities at increasing saturation time (build-up curves) for ligand **2** in complex with CTB[1]. b) CORCEMA-ST calculated STD intensities for the lowest energy docking solution of the complex (occupying both the galactose and sialic binding sub-sites; see Figure 1 in the main text for protons nomenclature).

HREMD Simulations of the CTB pentamer

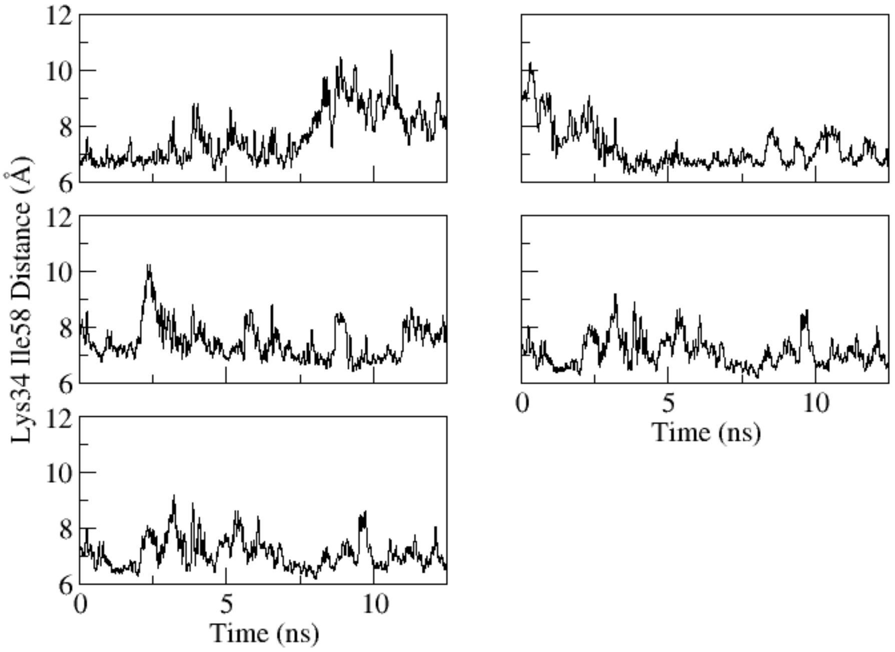

Figure S13. Distance between Lys34 and Ile58 sidechains of CTB over the course of a 12.5 ns HREMD simulation. Distances calculated from geometric center of heavy atoms. Each of the five plots represents a different subunit of the CTB homo-pentamer.

HREMD Simulations of the CTB pentamer bound to GM1

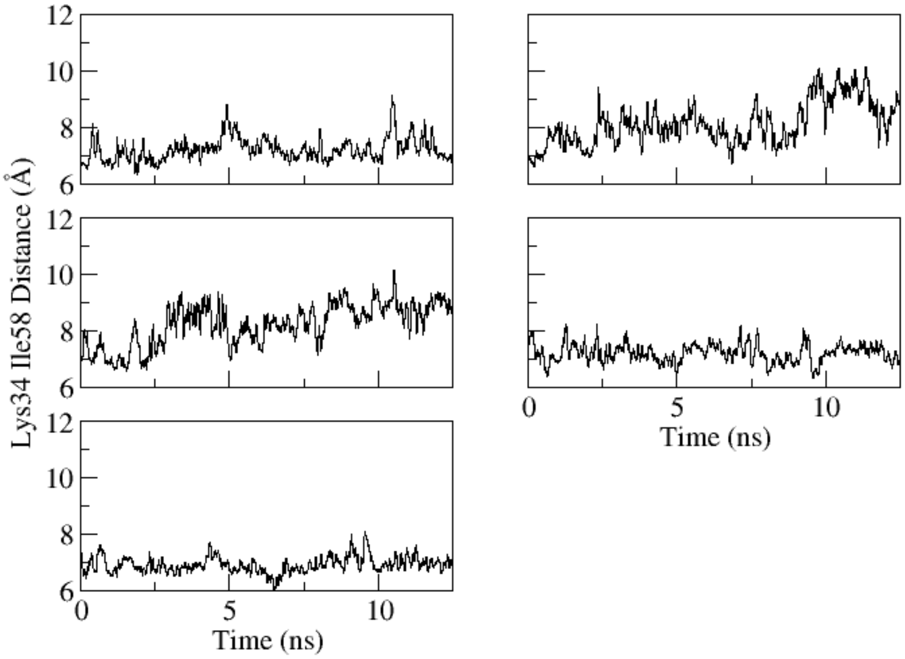

Figure S14. Distance between Lys34 and Ile58 sidechains of CTB over the course of a 12.5 ns HREMD simulation in which CTB is in complex with GM1. Distances calculated from geometric center of heavy atoms. Each of the five plots represents a different subunit of the CTB homopentamer.

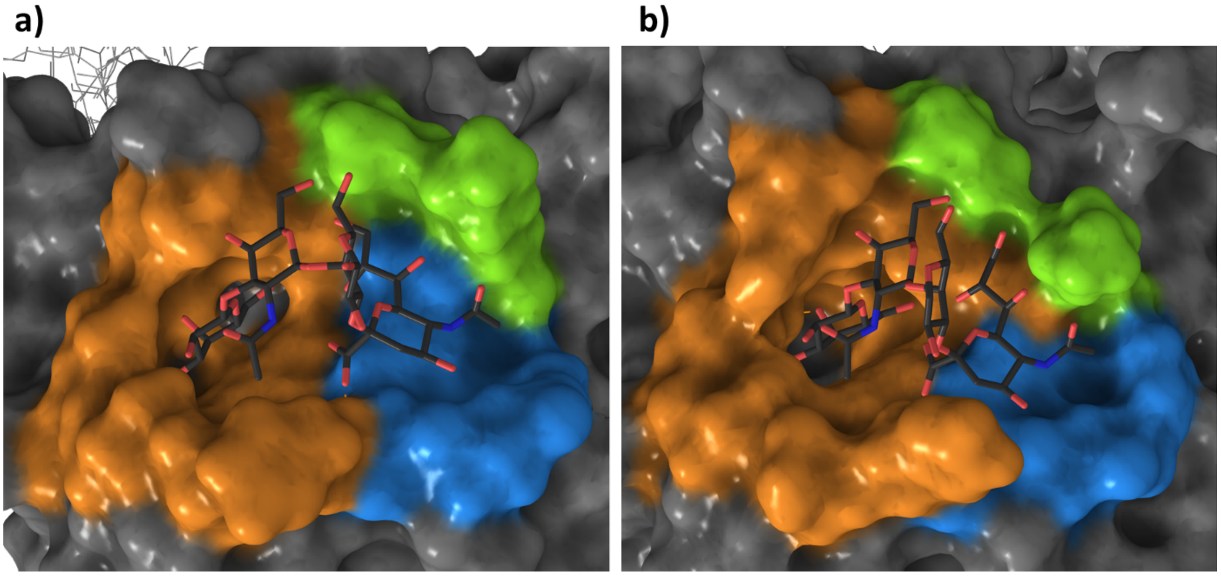

Figure S15. a) Top view of the XRD structure of the GM1/CTB complex (PDB ID: 3CHB[4]); **b)** Top view of the main frame from the MD simulation of the GM1/CTB complex. The reducing glucose has been omitted for clarity. Lys34 and Ile58, which define the novel binding subsite are shown in green**.**

MD Simulations of the CTB pentamer bound to ligand 3

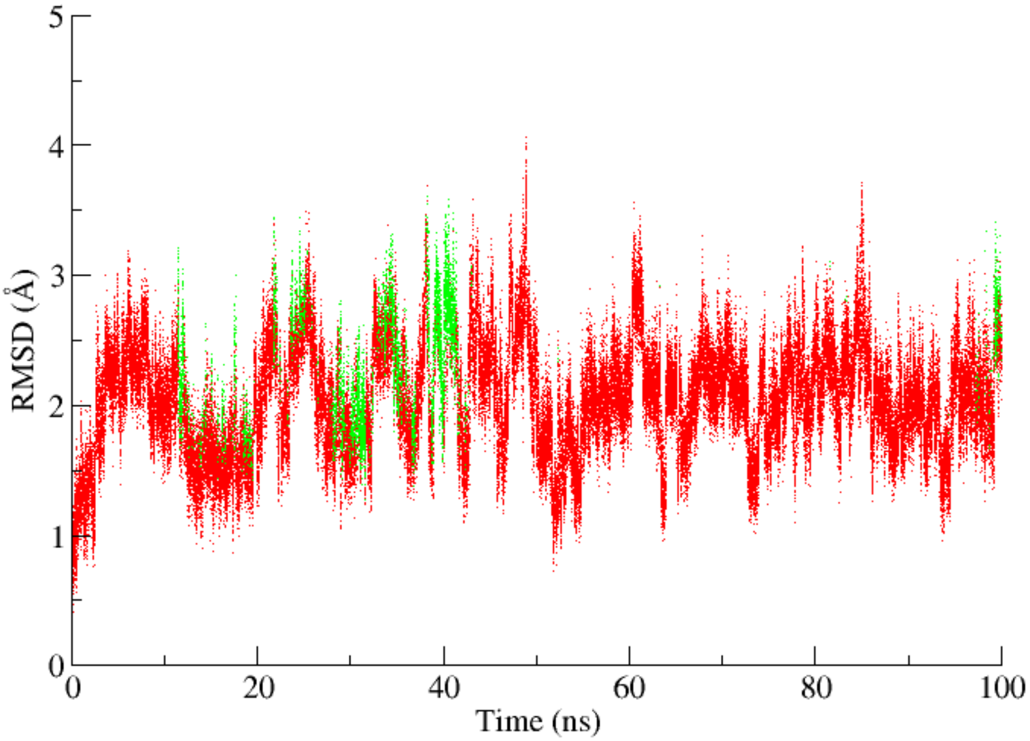

#Cluster	Frames	Frac	AvgDist (Å)	Stdev (Å)
0	93229	0.932	1.632	0.528
1	6771	0.068	1.002	0

Figure S16. Top: Root mean squared deviation (RMSD) of ligand **3** over the course of a 100 ns simulation in which the ligand is bound to CTB. RMSD determined by first performing a best fit to CTB backbone heavy atoms, before performing a no-fit calculation on the ligand heavy atoms. First frame used as reference. Cluster 0 and 1 shown as red and green points respectively. **Bottom**: Clustering statistics for the simulation, based on an average linkage hierarchical agglomerative algorithm with a cutoff of 2.5 Å fitting the heavy atoms of ligand **3**. Table shows fraction of frames in each cluster, average distance from the centroid and standard deviation. Cluster 0 shown in main text (Figure 9).

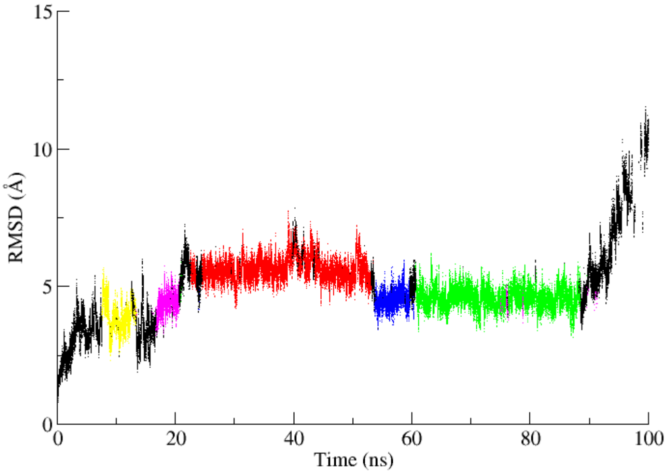

#Cluster	Frames	Frac	AvgDist	Stdev
0	29304	0.293	1.805	0.512
1	27726	0.277	1.574	0.438
2	6157	0.062	1.602	0.434
3	5527	0.055	2.072	0.609
4	4449	0.044	1.774	0.438
5	3920	0.039	2.026	0.57
6	3565	0.036	2.008	0.517
7	2783	0.028	1.931	0.492
8	2367	0.024	2.006	0.541
9	2341	0.023	1.866	0.488
10	2279	0.023	2.243	0.475
11	1916	0.019	1.626	0.403
12	1681	0.017	1.698	0.458

Figure S17. Top: Root mean squared deviation (RMSD) of ligand **3** over the course of a 100 ns simulation in which the ligand is bound to CTB. RMSD determined by first performing a best fit to CTB backbone heavy atoms, before performing a no-fit calculation on the ligand heavy atoms. First frame used as reference. Cluster 0 to 4 shown as red, green, blue, yellow, magenta points respectively. All other clusters shown in black. **Bottom**: Clustering statistics for the simulation, based on an average linkage hierarchical agglomerative algorithm with a cutoff of 2.5 Å fitting the heavy atoms of ligand **3**. Clustering yielded 26 clusters; those representing less than 1% population are omitted for clarity. Table shows fraction of frames in each cluster, average distance from the centroid and standard deviation. Cluster 0 shown in main text (Figure 9).

CORCEMA-ST validation ligands 3 and 2 (from MD frames)

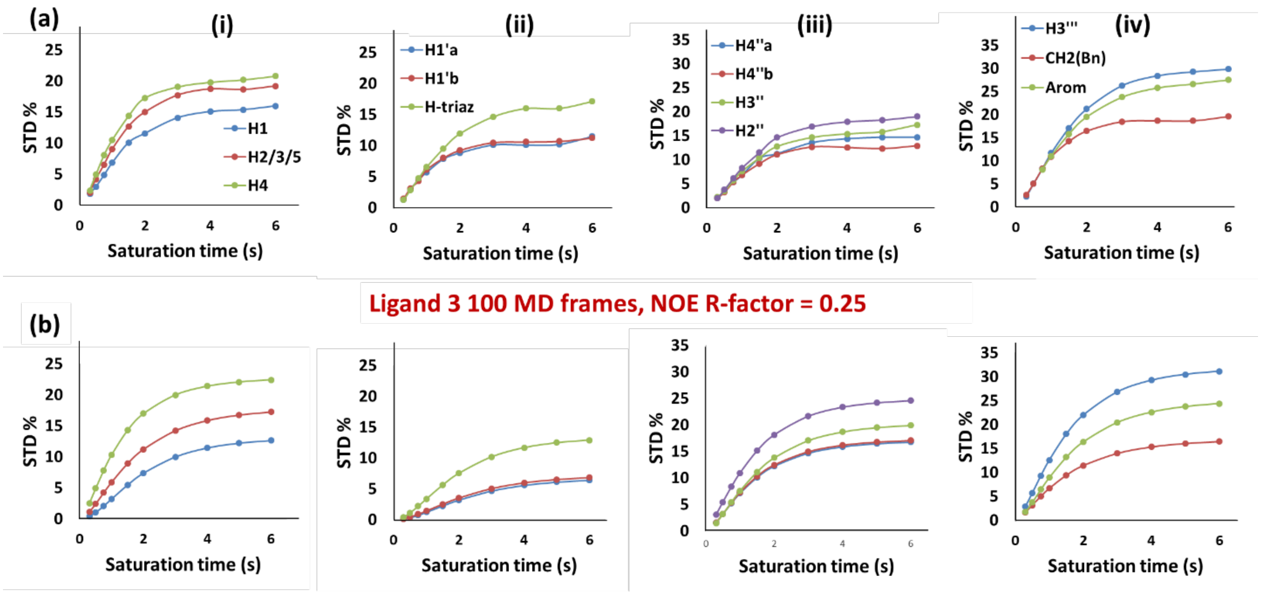

Figure S18. Ligand **3**, CORCEMA-ST calculations on MD frames. a) Experimental STD intensities at increasing saturation time (build-up curves) for ligand 3 in complex with CTB[1]. b) CORCEMA-ST calculated STD intensities for 100 averaged frames from MD of the complex (see Figure 1 in the main text for protons nomenclature).

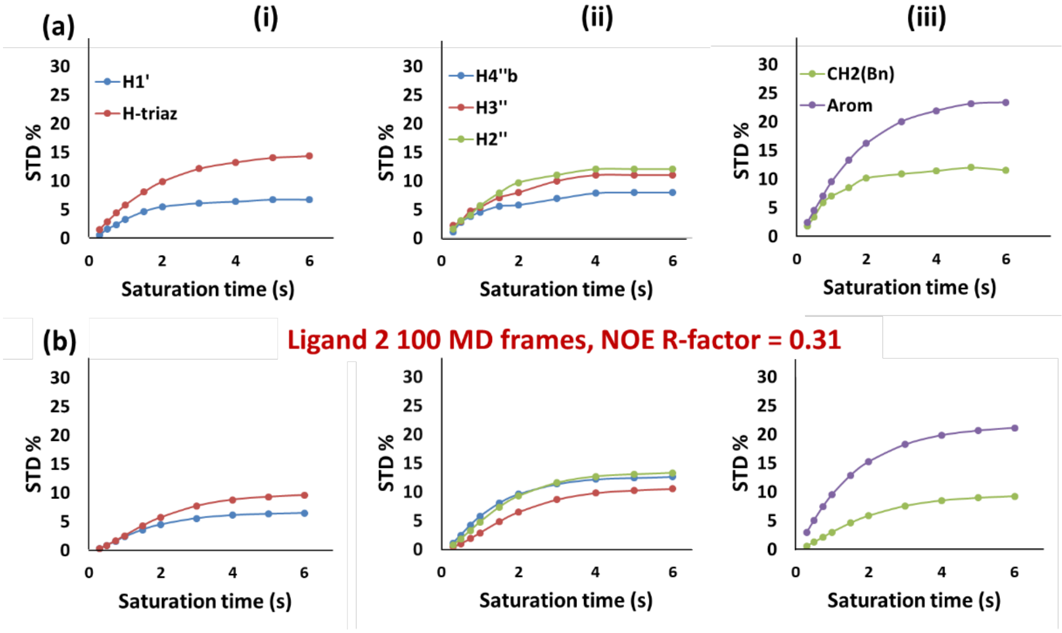

Figure S19. Ligand **2**, CORCEMA-ST calculations on MD frames. a) Experimental STD intensities at increasing saturation time (build-up curves) for ligand **2** in complex with CTB[1]. b) CORCEMA-ST calculated STD intensities for 100 averaged frames from MD of the complex (see Figure 1 in the main text for protons nomenclature).

References

1. Ramos-Soriano, J., et al., *Synthesis, Biological Evaluation, WAC and NMR Studies of S-Galactosides and Non-Carbohydrate Ligands of Cholera Toxin Based on Polyhydroxyalkylfuroate Moieties.* Chem. Eur. J., 2013. **19**(52): p. 17989-18003.
2. Yamamoto, K., et al., *Design, synthesis, and enzymatic property of a sulfur-substituted analogue of trigalacturonic acid.* Bioorg. Med. Chem. Lett., 2005. **15**(22): p. 4932-4935.
3. Jayalakshmi, V. and N.R. Krishna, *Complete relaxation and conformational exchange matrix (CORCEMA) analysis of intermolecular saturation transfer effects in reversibly forming ligand–receptor complexes.* J. Magn. Reson., 2002. **155**(1): p. 106-118.
4. Merritt, E.A., et al., *Crystal structure of cholera toxin B-pentamer bound to receptor GM1 pentasaccharide.* Protein Sci., 1994. **3**(2): p. 166-175.
